# The Western Diet Regulates Hippocampal Microvascular Gene Expression: An Integrated Genomic Analyses in Female Mice

**DOI:** 10.1038/s41598-019-55533-9

**Published:** 2019-12-13

**Authors:** Saivageethi Nuthikattu, Dragan Milenkovic, John Rutledge, Amparo Villablanca

**Affiliations:** 1 Division of Cardiovascular Medicine, University of California, Davis, Davis, California USA; 2 Université Clermont Auvergne, INRA, UNH, CRNH Auvergne, F-63000 Clermont-Ferrand, France

**Keywords:** Gene expression, Transcriptomics

## Abstract

Hyperlipidemia is a risk factor for dementia, and chronic consumption of a Western Diet (WD) is associated with cognitive impairment. However, the molecular mechanisms underlying the development of microvascular disease in the memory centers of the brain are poorly understood. This pilot study investigated the nutrigenomic pathways by which the WD regulates gene expression in hippocampal brain microvessels of female mice. Five-week-old female low-density lipoprotein receptor deficient (LDL-R−/−) and C57BL/6J wild type (WT) mice were fed a chow or WD for 8 weeks. Metabolics for lipids, glucose and insulin were determined. Differential gene expression, gene networks and pathways, transcription factors, and non-protein coding RNAs were evaluated by genome-wide microarray and bioinformatics analysis of laser captured hippocampal microvessels. The WD resulted in differential expression of 2,412 genes. The majority of differential gene expression was attributable to differential regulation of cell signaling proteins and their transcription factors, approximately 7% was attributable to differential expression of miRNAs, and a lesser proportion was due to other non-protein coding RNAs, primarily long non-coding RNAs (lncRNAs) and small nucleolar RNAs (snoRNAs) not previously described to be modified by the WD in females. Our findings revealed that chronic consumption of the WD resulted in integrated multilevel molecular regulation of the hippocampal microvasculature of female mice and may provide one of the mechanisms underlying vascular dementia.

## Introduction

Alzheimer’s Disease (AD) and vascular dementia (VaD), the second most common cause of dementia, are characterized by continual reduction in cognitive function and deterioration of memory, with the etiology likely due to environmental and genetic factors^1^, with the strongest genetic risk factor for AD being the ε4 variant of apolipoprotein E (ApoE). Amyloid plaques, neurofibrillary tangles, and large-scale loss of neurons and synapses are key AD pathologies^1^, whereas cerebral small vessel disease is the most common cause of VaD^2^. Interestingly, cardiovascular disease (CVD) risk factors are now also understood as contributing to the risk of AD and VaD^1^, and studies have shown significant overlap between risk factors for dementia and CVD, although the mechanistic link has not been clearly investigated^3^. Therefore, understanding the role of CVD risk factors is important as vascular risk factors are potential important targets for dementia prevention.

The association and overlap between CVD risk factors implicated in dementia, cognitive impairment, and small vessel disease in the brain are complex and not fully understood. One vascular factor involved in the development of neurodegenerative disorder is aging. Another is obesity^4^. Thus, increasing age, together with the metabolic effects of obesity, likely contribute to cognitive decline and incidence of dementia. The increase in obesity worldwide has been attributed to a shift in dietary habits towards diets with a high proportion of fat and refined sugars, reduced complex carbohydrate and fiber intake, and reduced fruit and vegetable consumption–diets known as a Western type diet (WD). One sequela of the WD is hyperlipidemia. In this context, high concentrations of low-density lipoprotein cholesterol (LDL-C) and total cholesterol (TC) are associated with an increased risk of AD^5^, and higher intake of saturated fat is implicated in impaired cognitive function, working memory, and attention and inhibitory control^6^. In addition, several clinical trials have shown that consumption of a high-fat diet significantly deteriorates attention capacity and processing speed^7^. We have also recently shown that the WD results in cognitive dysfunction in low density lipoprotein receptor (LDL-R) deficient male mice^8^.

Although lipids have a key role to play in the pathogenesis of dementia, the mechanisms by which they contribute to cognitive dysfunction in the brain are poorly understood and are likely multifactorial. It has been suggested that the blood-brain-barrier (BBB) is critical in most, if not all, cognitive dysfunction and neurodegenerative disorders since BBB dysfunction leads to inflammatory changes which contribute to the process of neuroinflammation and neurodegeneration^9,10^. The blood-brain barrier (BBB) is an interface between the peripheral circulation and the central nervous system. Anatomically, the BBB is the cerebral microvascular endothelium, which, together with astrocytes, pericytes, neurons, and the extracellular matrix, constitute the “neurovascular unit”. Disruption of endothelial cell function in wild type C57BL/6J mice given the WD for 12 weeks results in a significant increase in BBB permeability and decrease in cognitive function and memory^8^. In addition, exposure of endothelial cells to triglyceride-rich lipoprotein (TGRL) lipolysis products induces significant endothelial cell injury related to BBB dysfunction^11,12^. Furthermore, we have shown that in ApoE-deficient male mice the WD leads to ATF3-mediated pro-apoptotic, inflammatory and oxidative stress related neurovascular inflammation in brain microvessels, shedding light on one operative molecular mechanism^13^.

Using a targeted gene approach, lipids and high fat diets have also been shown to exert their harmful effects on cellular functions by modulating the expression of protein coding genes. In addition, protein non-coding RNA, such as miRNAs, have been implicated in regulation of BBB function^14^, lipid metabolism and the development of vascular disease^15^. Furthermore, transcription factors, including ATF3 and cyclic AMP-response element-binding protein (CREB) also play a role^13,16^. Other than gene regulation via transcription factors, additional complexity of regulation can proceed via post-transcriptional regulation by microRNAs (miRNA), a family of small non-coding RNAs that regulate gene function by inhibiting the expression of their target mRNAs. miRNAs play important roles in regulation of different cellular and subcellular functions and are recognized as modulators of dendritic and synaptic maturation and synaptic activity, which in turn modify cognitive performance^17^. Interestingly, a high fat diet can modulate expression of miRNAs, such as miR-690, miR-30e, miR-10a-5p, miR-21a-3p, miR-511-3p, miR-690 or miR-8112, in mouse brain elucidating the contribution of microRNAs in cognitive dysfunction induced by diet^17^. However, how these molecular pathways interact with female sex and diet remains largely unexplored in experimental models, and remains of interest to our work.

LDL-R deficient mice have been commonly used as models for studying atherosclerosis and dietary lipid stress because the LDL receptor plays a crucial role in clearing ApoE-containing lipoproteins^18^. The absence of LDL receptors prolongs the life of VLDL and LDL in the blood, making LDL-R deficient mice a particularly useful model for studying the relationship between lipid metabolism and inflammatory processes^18^, and this is of relevance to understanding neurovascular inflammation and the vascular determinants of dementia. Therefore, the aim of this study was to evaluate the mechanisms whereby the WD differentially regulates gene expression in the vasculature of the hippocampus as a key memory center. Specifically, we investigated the molecular regulation in brain hippocampal microvessels by performing global transcriptomic analyses on laser-capture isolated microvessels from brains of LDL-receptor deficient female mice. In this study, we hypothesized that the WD would lead to complex genomic effects that would result in differential gene expression of previously unreported protein coding *and* non-protein coding genes in the female brain hippocampal microvasculature.

## Methods

### Experimental animals

Reproductively intact five week old female low-density lipoprotein receptor deficient (LDL-R−/−) mice (strain B6.129S7-Ldlr tm1Her/J, Jackson Laboratories, Bar Harbor, ME) and C57BL/6J wild type (WT) mice (Jackson Laboratories, stock 000664) were fed either a standard chow control diet (CD = Chow, Nestlé Purina PetCare Co., St. Louis, MO) or a Western Diet (WD, catalog no. 88137, Harlan Laboratories, Madison, WI) composed of 21% fat and 0.2% cholesterol (w/w) for 8 weeks. There were four experimental treatment groups randomly assigned to the diets: WT fed CD, WT fed WD, LDL-R −/− fed CD, and LDL-R fed WD; with 7 mice/group. Animals were housed 2–3/cage in a temperature- and humidity-controlled environment with a 12 h light/dark cycle in the University of California, Davis Mouse Biology Program. Body weight was measure at baseline and at the completion of the dietary intervention period, and activity and food intake monitored daily by vivarium staff. Research was conducted in conformity with the Public Health Service Policy on Humane Care and Use of Laboratory Animals, and all protocols approved by the Institutional Animal Care and Use Committee of the University of California, Davis.

### Blood metabolic and hormone assays

Fasting lipid levels were measured in serum samples that were stored at −80C until assayed. Triglyceride (TG), total cholesterol (TC), high-density lipoprotein cholesterol (HDL), and low-density lipoprotein cholesterol (LDL) were measured using enzymatic assays from Fisher Diagnostics (Middleton VA), and precipitation separation from AbCam (Cambridge, MA) adapted to a microplate format. Fasting glucose and insulin levels were also measured on serum samples. Glucose was measured using enzymatic assays from Fisher Diagnostics (Middleton VA), and insulin was determined by electrochemiluminescence from Meso Scale Discovery (Rockville, MD) according to the manufacturer’s instructions. Estradiol was measured using enzyme-linked immune sorbent assay (ELISA) from CalBiotech (El Cajon, CA). All assays were performed by the UC Davis Mouse Metabolic Phenotyping Center (MMPC) in triplicate, on non-pooled plasma samples.

### Isolation and cryosection of murine brain hippocampus

Following completion of the dietary feeding period, mice were anesthetized by intraperitoneal xylazine/ketamine and euthanized by exsanguination during the light phase of their light/dark cycle, then intravascularly perfused with DEPC-treated PBS. Intact brains were rapidly removed under RNAse free conditions, cut into regions including the temporal lobe segment, and embedded using HistoPrep Frozen Tissue Embedding Media (Fisher Scientific, Pittsburgh, PA). To identify the hippocampus and hippocampal neurons, brain sections in the medial aspect of the temporal lobe were stained with hematoxylin and visualized with microscopy by a histopathology expert at UC Davis (Dr. Dennis Wilson). The hippocampus was then coronally cryosectioned (8 µm, Leica Frigocut 2800n Cryostat, Leica Biosystems, Buffalo Grove, IL). Hippocampal cryosections were placed on charged RNA-free PEN Membrane Glass slides, treated with RNA*later*®-ICE (Life Technologies, Grand Island, NY) to prevent RNA degradation, and stored at −80 °C until use. When ready for use, cryosections from the hippocampal segments were submerged in nuclease-free water and dehydrated in desiccant.

### Laser capture microdissection (LCM) of hippocampal microvessels

**Figure 1:**
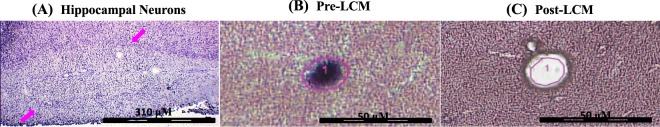
Representative images of hippocampal neurons and microvessels dissected by laser capture microdissection. Neurons in the hippocampus of Western diet (WD)-fed and control diet (CD)-fed low density lipoprotein receptor (LDL-R) −/− and C57BL/6J (WT) female mice were identified by hematoxylin staining (panel A, pink arrows). Microvessels in the hippocampus were identified by alkaline phosphatase staining and subjected to laser capture microdissection (LCM). The middle panel (panel B) shows the outline of an entire microvessel pre-LCM, and the right panel (panel C) shows the same microvessel post-LCM. Scale bar = 310 um panel A, and 50 μm panels B and C.

For analysis of gene transcriptome of hippocampal brain microvessels, endothelial microvessels (<20 um) were first identified in the hippocampal brain cryosections by alkaline phosphatase staining utilizing 5-bromo-4-chloro-3-indolyl phosphate/nitro blue tetrazolium chloride (BCIP/NBT) substrate as previously described^19^. Laser capture microdissection (LCM) was then used to isolate the endothelium of the microvessels within the hippocampal sections by capture of the entire vessel wall under direct microscopic visualization using a Leica LMD6000 Laser Microdissection Microscope (Leica Microsystems, Wetzlar, Germany), Fig. 1. In all 150 microvessels were captured per animal. Microvessels were not categorized by hippocampal region or subregion, although they primarily corresponded to endothelial enriched sections in hippocampus dorsal segments that would have included CA1 and CA3 regions.

### RNA extraction from laser captured brain microvessels

Total RNA was extracted from the laser-captured hippocampal brain microvessels (100 microvessels/sample) from each of the four experimental animal groups using an Arcturus PicoPure™ RNA Isolation Kit (Thermo Fisher Scientific, Santa Clara, CA) according to the manufacturer’s instructions. The quality of the RNA from the LCM-derived vessels was assessed by Nanodrop, and RNA integrity verified by qRT-PCR of control gene transcription (GAPDH). RNA quantification was performed according to Affymetrix RNA quantification kit with SYBR Green I and ROX™ Passive Reference Dye protocol (Affymetrix, Santa Clara, CA).

### Microarray hybridization and transcriptome analysis

For transcriptomics analysis, we used Affymetrix GeneChip Mouse Gene 2.0 ST Array (~28,000 coding transcripts and ~7,000 non-coding transcripts, Affymetrix, Santa Clara, CA). RNA (125 pg) was used to prepare cRNA and sscDNA using Affymetrix GeneChip® WT Pico Kit. SscDNA (5.5 ug) was fragmented by uracil-DNA glycosylase (UDG) and apurinic/apyrimidinic endonuclease 1 (APE 1) and labeled by terminal deoxynucleotidyl transferase (TdT) using the DNA Labeling Reagent that is covalently linked to biotin. Fragmented and labelled ssCDNA samples in triplicate were then submitted to the UC Davis Genomic shared resource core for hybridization, staining, and scanning using Affymetrix WT array hybridization protocol following the manufacturer’s protocol. Hybridization of fragmented and labelled ssCDNA samples was done using GeneChip™Hybridization Oven 645, and samples then washed and stained using GeneChip™ Fluidics Station 450. The arrays were scanned using GeneChip™ Scanner 3000 7G (Thermo Fisher Scientific, Santa Clara, CA). Quality control of the microarrays was done using Affymetrix Expression Console software version 1.4.1 and data analysis performed using Affymetrix Transcriptome Analysis Console software version 3.1.0.5.

### qRT-PCR analysis of gene expression in murine hippocampal *microvesselzs*

To corroborate the microarray analysis results, we performed qRT-PCR on 9 randomly selected differentially expressed RNA transcripts. For these experiments, RNA (75 ng) from the laser-captured brain microvessels was reverse transcribed into cDNA using iScript Reverse Transcription Supermix for RT-Qpcr (Biorad, Hercules, CA). qRT-PCR for selected genes was performed in ABI Vii7 Sequence detection system (PE Applied Biosystems, Foster City, CA). Reactions were carried out in 384-well optical plates containing 25 ng RNA/well and SsoAdvanced™ Universal SYBR® Green Supermix as fluorescent reporter (Biorad, Hercules, CA). Specific primers were designed with Primer3 software^20^ using the gene sequences obtained from Affymetrix transcript IDs. The sequences of the primers used are listed in the Supplement, Table S1. The PCR amplification parameters were: initial denaturation step at 95 °C for 10 minutes followed by 40 cycles, each at 95 °C for 15 seconds (melting) and 60 °C for 1 minute (annealing and extension). For protein coding genes, gene expression was normalized to glyceraldehyde-3-phosphate dehydrogenase (GAPDH) transcription, and for non-coding genes, gene expression was normalized to small nucleolar RNA 68 (SNORNA68) transcription. Relative gene expression was calculated using the delta-delta comparative threshold cycle (Ct) method and expressed as fold-change compared to wild type (WT) mice fed with control diet (CD).

### Bioinformatic analysis

Bioinformatics analysis of differentially expressed genes was performed by two of the study investigators (SN and DM) using multiple software tools. We compared each study group (LDL-R −/− WD, LDL-R−/− CD, and WT WD) to the control (WT CD). It was not possible to completely blind the analysis to study groups since study groups were compared to the WT CD group and it was necessary to identify the groups prior to analyses. For fold-change calculations it was also necessary to input experimental group data and compare it to control group data. This information is required by the microarray software (Affymetrix Transcriptome Analysis Console, version 3.1.0.5) used in the project.

Canonical pathway analysis was conducted using GeneTrial2 online database^21,22^ and Metacore software package^23^ to identify significantly over represented pathways. Enrichment statistics were calculated for these data sets assuming a hypergeometric distribution to identify significantly over represented pathways. Gene network and transcription factor analyses were performed using Metacore™. Protein-protein interactions were searched using String online tool^24,25^. Although protein interactome complexity analysis will depend on the number of genes inputted into the database and the size of the network, our analysis allowed for an additional up to 5 level gene interaction. MicroRNA validated targets were searched using the miRWalk database^26^ that enables retrieval of experimentally verified miRNA-gene target interactions. Hierarchical clustering and heat map representations of miRNA profiles were performed using PermutMatrix software^27,28^. Venn diagrams were generated using Venny^29,30^. Partial least squares discrimination analysis (PLSDA) was used to predict either continuous or discrete/categorical variables using MetaboAnalyst^31,32^.

### Statistical methods

For microarray, two-way ANOVA (Affymetrix Transcriptome Analysis Console software, Santa Clara, CA) was used for statistical analysis of microvessel transcriptome of WD fed WT mice, CD fed LDL-R −/− mice, and WD fed LDL-R −/− mice, each compared to CD fed WT mice. All genes from microarray with p < 0.05 and ±2.0-fold change were considered as differentially expressed. Mean body weight and plasma lipid levels of all 4 diet/genotype groups (CD fed WT, WD fed WT, CD fed LDL-R −/−, WD fed LDL-R −/−) were expressed as means ± standard error of the mean (SEM), and significance determined at p ≤ 0.05 using unpaired student’s t-tests (GraphPad software, La Jolla, CA). qRT-PCR determined gene expression in hippocampal microvessels of experimental mice, compared to CD fed WT mice, was expressed as log2-fold change, and statistical significance determined by unpaired student’s t***-***tests (GraphPad software, La Jolla, CA).

## Results

### Model of hyperlipidemia

We were able to attain the desired lipid stress in our animal model. After 8 weeks on the experimental diets, mean total cholesterol levels in the CD and WD fed WT mice were 73.9 mg/dL and 119.8 mg/dL (p < 0.05), respectively, and 225.7.0 mg/dL and 1259.6 mg/dL (p < 0.05) for the CD and WD fed LDL-R −/− mice, respectively; Supplemental Table S2(A). We also determined blood glucose and insulin levels in our study mice. Compared to CD fed WT mice, glucose levels were highest and significantly greater (p < 0.05) in the WD fed groups **(**Supplemental Table S2(B). Insulin levels were highest and significantly greater (p < 0.05) in the LDL-R −/− genotype compared to the WT mice. These results are consistent with what has been published previously for these experimental models^33,34^.

In addition, the dietary treatment resulted in the expected weight gain in the study mice as follows: mean weight for WT mice at baseline was 16.3 g and increased by an average of 16% when fed with CD and 42% when fed with WD; mean weight of LDL-R −/− mice at baseline was 14 g and increased by an average of 36% when fed with CD and 68% when fed with WD, (p < 0.05 respectively for all group comparisons); Supplemental Fig. S1.

### Effect of the western diet on brain hippocampal microvessel gene expression

To define molecular mechanisms in brain hippocampal microvessels in response to the WD, we began by assessing the effect of the WD on the global expression of genes in hippocampal microvessels of CD fed and WD fed WT and LDL-R −/− female mice. These studies showed that among the 34,472 genes studied in the microarrays, 2,412 genes (7%) were differentially expressed (DE). Volcano plots of the significantly up- or down-regulated genes in brain microvessels of WD fed WT mice show up-regulation of 168 genes and down-regulation of 143 genes compared to microvessels of CD fed WT mice (Supplemental Fig. 2A, Table S3 for a complete listing of the DE genes). In contrast, in microvessels of WD fed LDL-R −/− mice, there was up-regulation of 299 genes and down-regulation of 260 genes compared to CD fed WT mice (Supplemental Fig. 2C, see Supplemental Table S5, for a complete listing of the DE genes). The effect of the WD was contrasted to the effect of genotype by comparing differential gene expression in CD fed LDL-R −/− mice to CD fed WT mice and revealed up-regulation of 1,394 genes and down regulation of 148 genes (Supplemental Fig. 2B, see Supplemental Table S4 for a complete listing of the DE genes). This data suggests a strong effect of the WD on differential gene expression in brain microvessels of female mice, as well as an important contribution of the LDL-R −/− genotype.

A random sample of nine differentially expressed protein coding and non-coding genes, representative of each of the experimental genotype/diet groups, were tested by qRT-PCR and confirmed to have the same direction of change in gene expression (up- or down-regulation) as observed with the microarrays (Supplemental Fig. S3).

**Figure 2:**
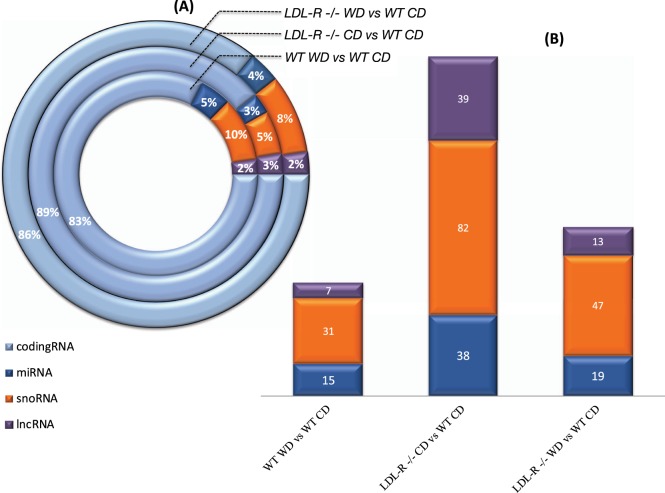
Distribution of differentially expressed RNAs in hippocampal microvessels. (**A)** percentage of differentially expressed protein-coding (light blue) and non-coding RNAs, and (**B)** number of differentially expressed non-protein coding RNAs (microRNAs = miRNAs, dark blue; small nucleolar RNA = snoRNAs, orange; and long non-coding RNA = lncRNAs, purple) in microvessels from C57BL/6J western diet (WD) fed (WT) mice compared to microvessels from control diet (CD) fed WT mice, CD fed LDL-R −/− mice compared to control diet (CD) fed WT mice, and WD fed LDL-R −/− mice compared to control diet (CD) fed WT mice.

To our knowledge, we show for the first time that the WD regulates expression of protein non-coding genes in female brain microvessels (Fig. 2A,B), including long non-coding RNAs (lncRNA), microRNAs (miRNAs), and small nucleolar RNAs (snoRNAs). The number of differentially expressed non-protein coding genes was greatest for snoRNAs (160 total) compared to the other non-coding RNAs (72miRNAs and 59 lncRNAs), and greatest (82 total) for CD fed LDL-R −/− mice, Fig. 2B.

### Effect of the western diet on expression of protein-coding genes in brain hippocampal microvessels

**Figure 3:**
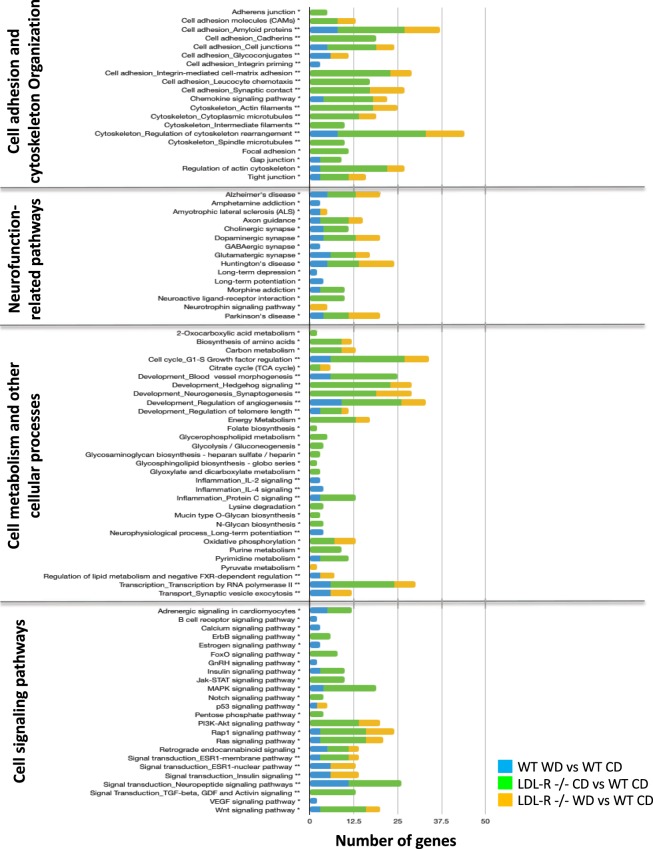
Histogram of subset of significant gene pathways and gene networks of differentially expressed protein-coding genes in hippocampal microvessels. Pathways and gene networks were identified using differentially expressed genes in microvessels from Western diet (WD) fed C57BL/6J (WT) mice compared to microvessels from control diet (CD) fed WT mice, CD fed LDL-R −/− mice compared to CD fed WT mice, and WD fed LDL-R −/− mice compared to CD fed WT mice. KEGG pathways (*) were identified using Genetrial2 online database, and gene networks (**) using MEtaCore software. Pathways are grouped by cellular function.

KEGG and MetaCore bioinformatics analysis of significantly differentially expressed protein-coding genes was performed to identify the cellular processes in which they were involved. We observed differential regulation of a number of important cellular pathways including those for cellular adhesion, junctional and cytoskeletal organization, neurological function, cellular metabolism, and cell signaling (Fig. 3). In general, when compared to CD fed WT mice, the greatest number of genes involved in differential expression of cellular pathways was observed for LDL-R −/− mice fed CD. Gene network analyses were in agreement with pathway analyses, and identified networks involved in the regulation of the same cellular processes, data not shown.

### Effect of the western diet on brain hippocampal microvascular protein-protein interactions

The bioinformatics analyses of gene expression data for each of the experimental diet/genotype group comparisons permitted identifying proteins encoded by the differentially expressed genes, as well as protein interactome complexity (Supplemental Fig. 4A–C, Table 1). This analysis revealed distinct patterns of protein-protein interactions in each experimental group, as well as proteins with a strong functional interactive pattern that represented key genes in the *in vivo* response of female brain hippocampal microvessels to the WD. When compared to CD fed WT mice, we observed the greatest number of nodes, over 40, having more than 15 protein-protein interactions for WD fed WT mice (Supplemental Fig. 4A) and CD fed LDL-R −/− mice (Supplemental Fig. 4B**)**, while a smaller number of nodes was observed for WD fed LDL-R −/− mice (Supplemental Fig. 4C). Among the complex protein nodes identified were those involved in the regulation of chemotaxis, ribosomal or focal adhesion, and leukocyte trans-endothelial migration.

**Table 1:** List of Proteins with more than 15 predicted protein-protein interactions (STRING database).

WT WD vs WT CD	LDLR KO CD vs WT CD	LDLR KO WD vs WT CD
Gm5239	Gm8730	Gm8730
Gm8730	Gm3839	Rpl9-ps6
Polr2a	Eif4a1	Mrps10
Rab11a	Gm10260	Gm17541
Mrpl16	Mrpl16	Psmb3
Cct4	Polr1c	Psma2
Rpl3	Gm5428	
Actr1b	Mapk3	
Stat3	Rps15a	
Mocs3	Rab11a	
Cnot6l	Rpl3	
Rpl9-ps6	Cct4	
Sec. 61a2	Gm6139	
Gm8225	Nhp2l1	
Nhp2l1	Mapk8	
Nop56	Nop56	
Rps15a	Eno1	
Srsf1	Eno3	
Jun	Rps24	
Psma3	Arrb2	
Pik3cd	Gm5039	
Rps10	Rps10	
Eno3	Taf1	
Gm9396	Actr1b	
Hdac5	Cnot6l	
Rps24	Hdac5	
Uba3	Stat3	
Gm7536	Arrb1	
Psmb3	Eef2	
Camk2d	Fgfr3	
Eef2	Gm9396	
Ptk2	Gm7536	
Sumo1	Ptk2	
Tceb1	Acta1	
Fyn	Gm6316	
Gm8394	Rpl17	
Psma1	Tcf7l2	
Eif3d	Ube2d2a	
Psma4	Eif3d	
Rab9	Ppp2r2a	
Sgk1	Aldh5a1	
Snrpa	Apc	
	Nsa2	
	Btf3l4	
	Eif4h	
	Gm4968	
	Hist1h3i	

### Potential transcription factors involved in the genomic effects of the western diet on brain hippocampal microvessels

**Figure 4:**
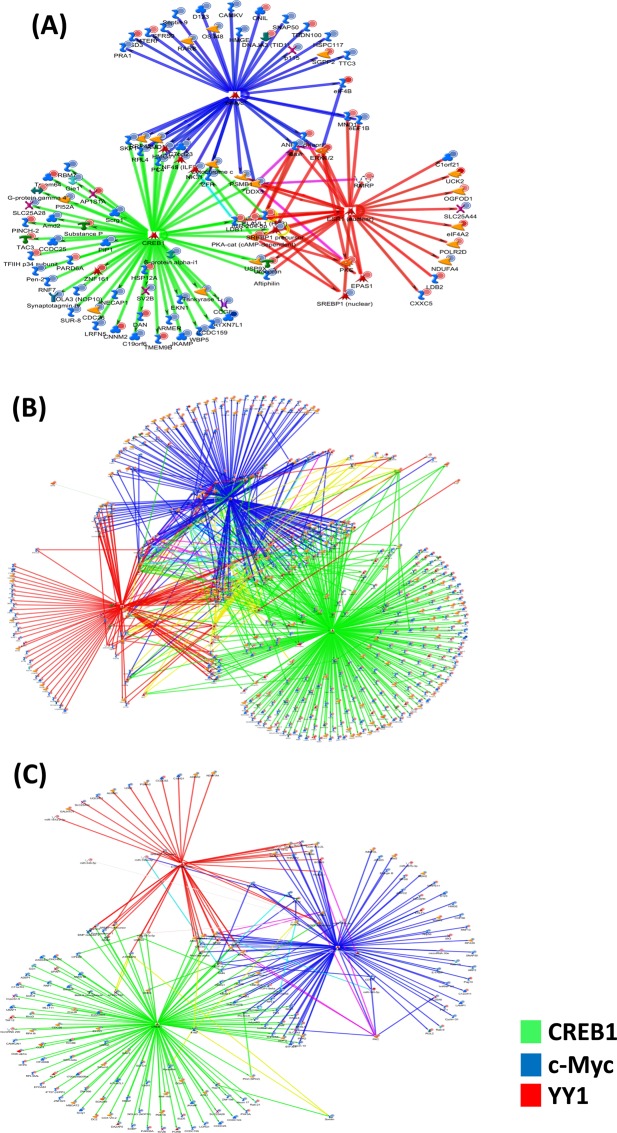
Transcription factors affected by the Western diet in hippocampal microvessels. Transcription factors potentially modulated by the Western diet were identified using MetaCore Transcription Regulation algorithm. Gene networks for the three most significant transcription factors: (**A)** CREB1 (green), c-Myc (blue) and ESR1 (red) for Western diet (WD) fed C57BL/6J (WT) mice compared to control diet (CD) fed WT mice; (**B)** CREB1 (green), c-Myc (blue) and YY1 (red) for CD fed LDL-R −/− mice compared to CD fed WT mice; and (**C)** CREB1 (green), c-Myc (blue) and ESR1 (red) for WD fed LDL-R −/− mice compared to CD fed WT mice.

We also performed bioinformatics analyses of gene expression data to identify potential transcription factors that could be involved in mediating the observed genomic effects following the WD. The top 30 transcription factors in our study groups are shown in Fig. 4 (and detailed in Supplemental Table S6**)**. The gene network targets of the top 3 potential transcription factors are also shown in Fig. 4A–C for each experimental diet/genotype comparison. The most statistically significant differentially expressed transcription factors were CREB1 (cAMP Responsive Element Binding Protein), c-Myc (cellular myelocytomatosis), ESR1 (estrogen receptor 1), and YY1 (Yin Yang 1). Figure 4A–C, Supplemental Table S6 depicts the gene networks for the three most differentially expressed transcription factors for WD fed WT mice (Fig. 4A; CREB1, c-Myc, and ESR1), CD fed LDL-R −/− mice (Fig. 4B; CREB1, c-Myc, and YY1), and WD fed LDL-R −/− mice (Fig. 4C; CREB1, c-Myc, and ESR1). Venn diagram comparison of the top 30 transcription factors among all of the study groups revealed 16 transcription factors in common (see listing in Supplemental Fig. S5).

### Impact of the western diet on expression of miRNA in brain microvessels

**Figure 5:**
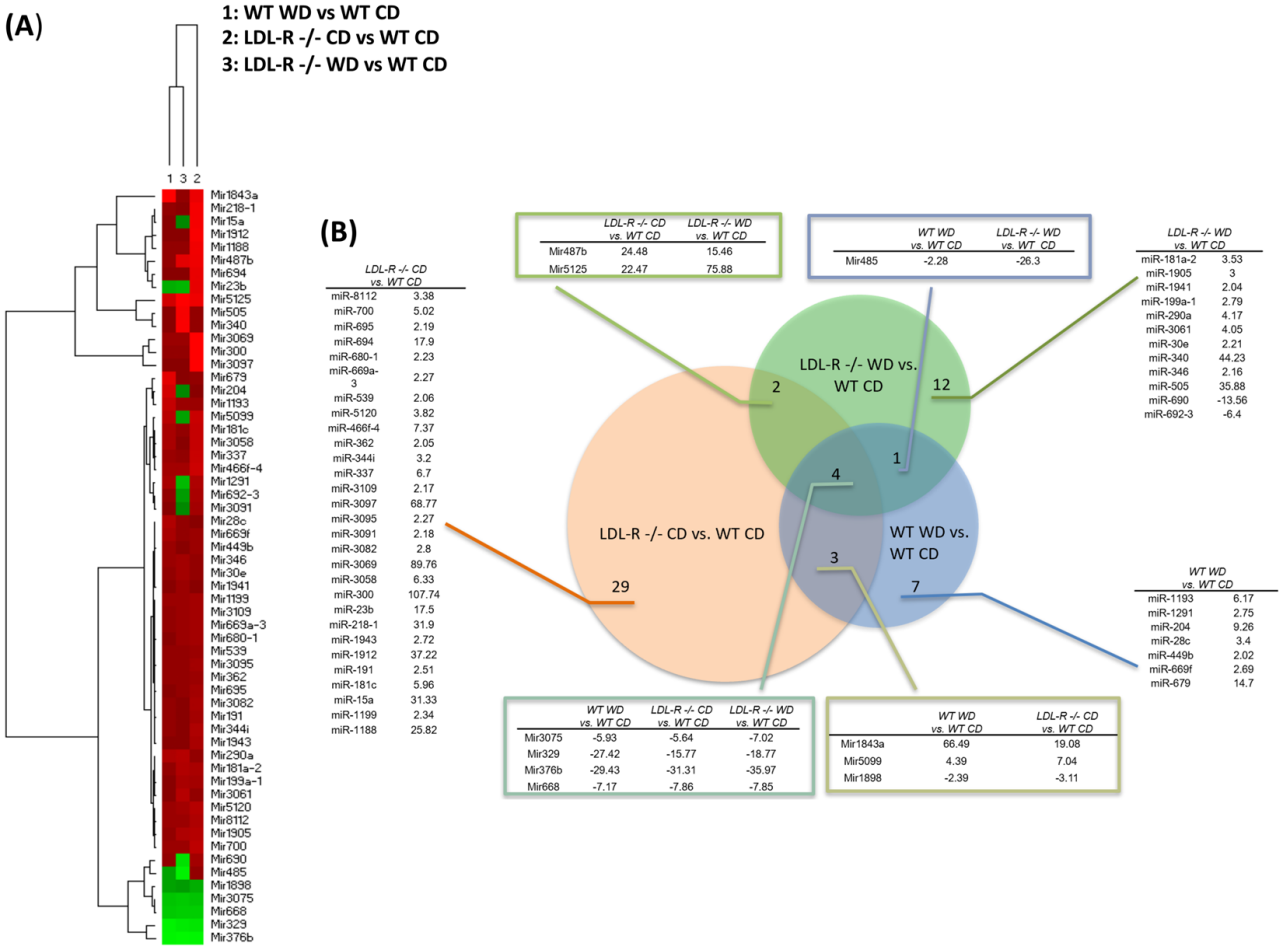
Effect of the Western diet on microRNA expression in hippocampal microvessels. (**A**) Heat map of expression profiles of microRNAs (miRNAs) identified as differentially expressed in at least one experimental dietary comparison group. Red indicates up-regulation and green down-regulation of gene expression compared to control [C57BL/6J wild type (WT) mice fed a control diet (CD)]. Individual miRNAs are represented in rows, and the three different experimental comparison groups in columns, as follows: column 1: Western diet (WD) fed C57BL/6J (WT) mice compared to control diet (CD) fed WT mice; column 2: CD fed LDL-R −/− mice compared to CD fed WT mice; column 3: Western diet (WD) fed LDL-R −/− mice compared to CD fed WT mice. (**B**) Venn diagram of differentially expressed miRNAs. Venn diagram displays common and distinct miRNAs among the experimental conditions compared to control [C57BL/6J (WT) fed control diet (CD)]. Numbers in the Venn diagram indicate number of common or distinct miRNAs for each experimental comparison. Numbers in the columns indicate fold-changes for each miRNA compared to control.

Two-dimensional hierarchical clustering analysis of differentially expressed miRNAs (Fig. 5A) revealed general up-regulation of miRNA gene expression in all study diet/genotype comparison groups. A few clusters of miRNAs with down-regulated expression profiles were primarily observed in WD fed LDL-R −/− mice compared to CD fed WT mice. In addition, a cluster of down-regulated miRNA expression was observed in all comparison groups for Mir1898, Mir3075, Mir668, Mir329, and Mir376b. To our knowledge, these results demonstrate for the first time that the WD modulates the expression of miRNAs in female brain microvessels.

Furthermore, miRNA expression analysis showed that the WD significantly modulated expression of 58 miRNAs in female brain hippocampal microvessels and revealed common and distinct miRNAs among the experimental conditions when compared to control mice, Fig. 5B. Specifically, when compared to CD fed WT mice, the WD fed WT mice had significant up-regulation of 7 miRNAs unique to this comparison with fold-changes ranging from + 2.02 to + 14.7. In contrast, in LDL-R −/− mice the WD significantly modulated expression of 12 miRNAs (10 up-regulated and 2 down-regulated with fold changes ranging from −13.56 to + 44.23) specific to this comparison. The LDL-R −/− genotype itself (CD fed LDL-R −/− mice) when compared to CD fed WT mice significantly modulated expression of 29 miRNAs unique to this comparison. These 29 differentially expressed miRNAs were up-regulated with fold-changes ranging from + 2.05 to + 107.74. In general, there was relatively little overlap in miRNA expression between the diet/genotype comparison groups. When compared to CD fed WT mice, only 3, 2 and 1 miRNAs were common between WD fed WT mice and CD fed LDL-R −/− mice, CD fed LDL-R −/− mice and WD fed LDL-R −/− mice, and WD fed WT mice and WD fed LDL-R −/− mice respectively. For all experimental conditions, there were only 4 miRNAs in common (Mir3075, Mir329, Mir376b, and Mir 668), all down-regulated.

To identify potential target genes for the observed differentially expressed miRNAs in the study groups, we used the miRWalk database (Supplemental Fig. S6A). When compared to CD fed WT mice, bioinformatic analysis identified 864 potential targets genes for differentially expressed miRNA in WD fed WT mice, 2,064 potential targets genes for miRNA in CD fed LDL-R −/− mice, and 2,035 potential targets genes for miRNA in WD fed LDL-R −/− mice. Comparison of the potential target genes of the differentially expressed miRNAs and differentially expressed genes revealed relatively little overlap (a total of 10, 123, and 46 genes in common for WD fed WT mice, CD fed LDL-R −/− mice, and WD fed LDL-R −/− mice, respectively, when compared to CD fed WT mice).

Pathway analysis of miRNA gene targets (Supplemental Fig. S6B) showed that compared to CD fed WT mice, there were 46, 63, and 92 miRNA target gene pathways in WD fed WT mice, CD fed LDL-R −/− mice, and WD fed LDL-R −/− mice, respectively. Venn diagrams for the differentially expressed gene pathways and miRNA target gene pathways showed that 34, 84, and 52 pathways, respectively, were in common for WD fed WT mice, CD fed LDL-R −/− mice, and WD fed LDL-R −/− mice, when compared to CD fed WT mice.

**Figure 6:**
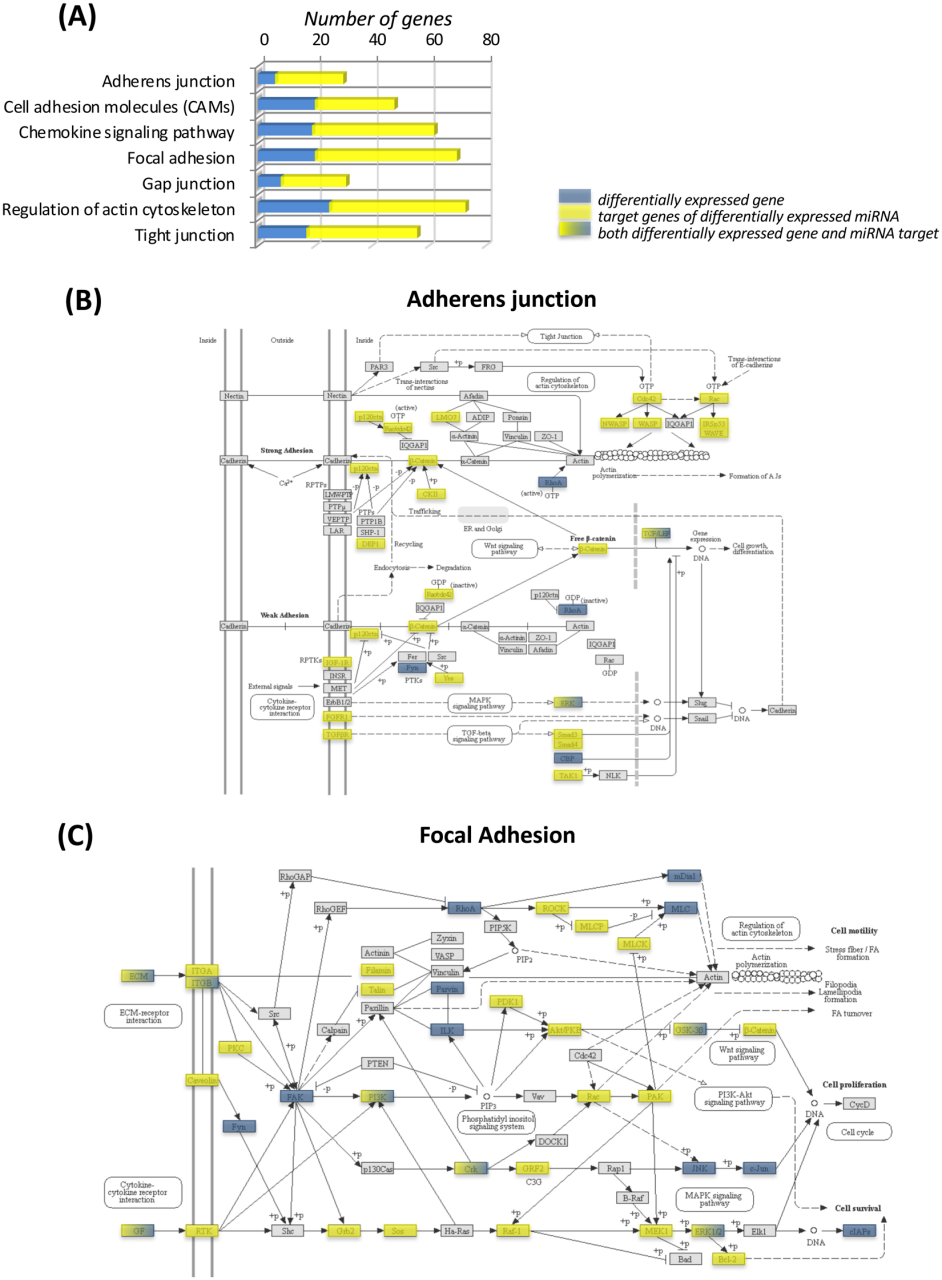
Functional integration of differentially expressed genes and target genes of differentially expressed miRNAs. (**A)** Histogram represents cellular pathways involved in the regulation of endothelial function obtained using differentially expressed genes (blue), and target genes of differentially expressed miRNA (yellow), identified in at least one experimental dietary study group compared to control [C57BL/6J (WT) fed control diet (CD)]. Representative integrated analysis of differentially expressed genes, and target genes of differentially expressed miRNAs for **(B)** adherens junctions and **(C)** focal adhesion pathways. Blue = differentially expressed genes; Yellow = target genes of differentially expressed miRNAs; Color gradation from Yellow to Blue = genes identified to be both differentially expressed and to be targets of differentially expressed miRNAs.

To determine the potential cellular function of the differentially expressed genes, and gene targets of the differentially expressed miRNAs, bioinformatics histogram analysis was performed and identified diverse cellular pathways, Fig. 6A–C. Comparison of the most over-represented pathways (Fig. 6A) showed the following common pathways for differentially expressed genes and miRNAs target genes: adherens junction, cell adhesion molecules (CAMs), chemokine signaling pathways, focal adhesion, gap junction, regulation of actin cytoskeleton, and tight junctions. We performed integrated analyses of differentially expressed genes, and target genes of differentially expressed miRNAs, for two of these pathways (Fig. 6B,C). For focal adhesion pathways, seven of the differentially expressed genes were also targets of differentially expressed miRNAs, namely, laminin, gamma 3 (ECM), integrin beta 1 (ITGβ), phosphatidylinositol-4,5-bisphosphate 3-kinase (PI3K), proto-oncogene C-crk (CRK), glycogen synthase kinase 3 beta (GSK-3β), epidermal growth factor (GF), and mitogen-activated protein kinase 1/2 (ERK1/2). For adherens junction pathways, two of the differentially expressed genes were also targets of differentially expressed miRNAs, namely, mitogen-activated protein kinase (ERK) and lymphoid enhancer-binding factor 1 (TCF/LEF). These finding suggest that modulation of differential gene expression in our system could be partially explained by differential miRNA expression.

### Impact of the western diet on expression of snoRNAs and lncRNAs in brain microvessels

The results detailed above showed that the majority of differential gene expression in our study system was attributable to differential regulation of cell signaling proteins and their transcription factors, approximately 7% was attributable to differential expression of miRNAs, and a lesser proportion of differential expression was due to other non-protein non-coding RNAs, primarily long non-coding RNAs (lncRNAs) and small nucleolar RNAs (snoRNAs). Regarding non coding RNAs, we identified differential expression of a total of 160 snoRNAs and 59 lncRNAs in the experimental groups when compared to control CD fed WT mice, Table 2. Differential expression for snoRNAs and lncRNAs consisted almost exclusively of their up-regulation. Only few of these snoRNAs (AF357425, snoRA2C and snoRA44)^35,36^ and LncRNAs (A630075F10Rik, SNHG7, Gm12603 and 2900097C17Rik)^37–40^ have known function in cognition, endothelial cell function and neuro-inflammation. However, most of the identified snoRNAs and LncRNAs have no known function. Thus, the Western diet was associated with a potentially newly described crucial layer of biological regulation via snoRNAs and lncRNAs.

**Table 2:** Effect of the Western diet on the expression of small nucleolar RNAs (snoRNAs) and long non-coding RNAs (lncRNAs) in female hippocampal micro-vessels.

snoRNAs	C57 WD vs Chow	LDLR KO Chow vs C57 Chow	LDLR KO WD vs C57 Chow
DQ267102	112.18		
Scarna3b	66.49	19.08	
Gm25744	35.4		
Snora44	18.16	24.25	
Gm25604	16.78		
Gm25357	11.71	45.7	100.22
Gm24095	10.74		
Gm25788	7.48	7.89	10.91
Snord42b	4.96		3.82
Gm25519	4.57		
Gm25224	4.46	64.19	3.39
Gm22188	3.05	4.32	−7.89
Gm23613	2.97		
Snora34	2.75		
Snord116	2.42		
Gm24519	2.39		
Gm22304	2.36		
Gm24175	2.18		
Gm25759	−2.45		
Gm22205	−3.04		
Gm23546	−6.14	−7.12	−5.49
AF357425	−6.5		10.25
Gm25635	−7.41	−7.58	
Gm26202	−9.27		−12.12
Gm23301	−9.45	−3.2	−7.44
DQ267100	−9.95	−14.82	−17.77
Gm22858	−10.53	−11.79	−8.25
Snord65	−18.49	−12.82	−14.13
Gm26265	−19.24		−16.39
Snord61	−33.17	−28.79	−34.09
Gm26347	−12.83	−18.09	−18.68
Gm22882		110.37	
Scarna13		89.76	
DQ267101		37.28	6.07
AF357428		36.2	
Gm25406		29.68	
Snora17		27.77	
Snord17		22.87	5.45
Gm25376		21.03	
Gm23508		20.16	9.62
Gm26225		17.92	
Snord104		15.69	
Snora30		14.11	
Snord34		14.08	
Gm24411		12.88	
Gm25053		11.33	4.44
Gm24556		10.43	
Gm22131		10.39	−6.57
Gm25973		10.16	
Snord55		8	
Gm25396		7.76	
Gm25615		7.55	
Gm25683		7.02	
Scarna2		6.87	3.22
Gm25093		6.5	
Gm23456		6.44	
Gm25128		6.23	
Gm26148		5.5	
Snord8		5.44	
Gm23745		5.36	
Gm22962		5.32	45.71
Gm22039		5	3.06
Gm24154		4.65	
Gm23266		3.85	
Gm23965		3.67	
Gm23443		3.55	
Gm25092		3.49	
Gm24670		3.41	2.64
Gm23123		3.34	
Gm24942		3.24	
Gm22497		3.23	
Snhg11		3.17	
Gm23300		3.09	
Gm23979		3.04	
Gm24727		2.98	3.77
Gm24339		2.7	
Gm23826		2.62	
Snora21		2.6	
DQ267102		2.59	13.43
Snord64		2.59	
Gm22900		2.58	
Gm26286		2.49	
Gm25274		2.47	
Gm25466		2.45	
Gm23951		2.44	
Snord73a		2.42	
Gm24900		2.3	
Gm25967		2.25	
Gm23321		2.19	
Snord11		2.13	
Gm22173		2.1	
Gm24212		2.09	
Gm25906		2.06	
Gm24988		2.05	
Gm22795		2.04	
Gm25434		2.03	
Gm23320		2.01	
Gm25432		−3.3	−3.16
Gm23644		−4.38	
Snord16a			26.26
Snora23			14.28
Gm22957			11.98
Gm24241			6.65
Gm24706			4.72
Gm24620			3.88
Gm24068			3.56
Gm25617			3.34
Gm24696			3.23
Gm25342			2.98
Gm23746			2.95
Gm22763			2.79
Gm26358			2.54
Gm26423			2.49
Gm24987			2.4
Gm24665			2.33
Gm26173			2.22
Gm22271			2.11
Gm25402			−2.49
Gm26236			−7.3

## Discussion

The present study was a large-scale transcriptome gene profiling of the hippocampal microvasculature of female mice in relation to hyperlipidemic conditions (western diet and/or LDL-R−/− genotype). Our results show profound transcriptome changes including the modulation of protein coding genes, miRNAs, snoRNAs and lncRNAs, as well as the corresponding cellular functional pathways, and the mechanism of regulation by transcription factors. We focused on molecular mechanisms of differential gene expression in brain microvessels due to their critical importance in the vascular determinants of dementia. However, as this was a preliminary study, we did not assess functional outcomes such as cognition or vascular permeability. Interestingly, our recently published work using a similar experimental model in male mice has demonstrated that the WD increased blood brain barrier (BBB) permeability and resulted in cognitive impairment^8^.

Our study demonstrated the expected significant differences in cholesterol and lipid levels between control diet (CD) and the high fat Western diet (WD) fed to LDL-R −/− mice. The control diet fed LDL-R −/− mice spontaneously demonstrated hyperlipidemia because of absence of the LDL receptor. The physiological significance of severe hyperlipidemia awaits further study, but has been shown in other study systems to correlate with accelerated atherosclerosis and vascular injury^18^. We also demonstrated changes in serum glucose and insulin that were consistent with those previously published for our experimental models. Specifically, glucose levels were highest in the WD fed groups and insulin levels were highest in the LDL-R −/− genotype.

It was not previously known how a high cholesterol diet affects the transcriptome of brain hippocampal microvessels in females. In our prior work using a candidate gene approach in male mice, we identified activating transcription factor 3 (ATF3) as a key regulator of neuro-inflammation^13^. The present study shows for the first time that in females, the WD significantly modulates differential expression of approximately 10% of the genome of microvessels in the hippocampus, including protein coding genes and non-coding genes (miRNAs, snoRNAs and LncRNAs).

We looked at a large data set of n = 2412 DE genes, including coding and non-coding genes, and the analysis suggests separation between diet and genotype. A comparison of gene expression profiles of ~ 150 genes by clustering identified two groups of genes with opposite expression profiles between diet and genotype (data not shown). This suggests that diet and genotype differentially impacted expression of a subset of genes. Bioinformatic analyses of all of the differentially expressed genes identified them to be implicated in multiple and complex cellular pathways including those regulating cell adhesion and cytoskeletal organization, neurofunction, cell junctions and chemotaxis. In addition, we identified several differentially expressed gene networks involved in disease states and biological processes including Alzheimer’s dementia, inflammation, and oxidative stress.

Adherent and tight junctions regulate permeability by maintaining neighboring endothelial cells together and by regulation of paracellular diffusion^41^. The WD in our study modulated expression of genes regulating the cell-cell junctions. Among these was down regulation of Claudin 5 (CLDN5) which has been observed to result in increased BBB permeability^42^, suggesting a potential genetic mechanism for WD-associated increased BBB permeability, a hypothesis in agreement with our previously reported work^8^. Another differentially expressed gene was the gene coding for ERK1/2 which has also been linked to impairment of BBB via a neuro-inflammatory response promoting leukocyte infiltration^43^. A significant increase in expression of Actin Related Protein 2/3 (Arp2/3) Complex Subunit 4 was also observed. The Arp2/3 complex is one of the principal actin-polymerizing and organizing factors. Activation of Arp2/3 induces neuro-inflammatory effects and disruption of the BBB, while inhibition of the Arp2/3 complex has been shown to attenuate a decrease in BBB permeability and increase transendothelial monocyte migration^44^. Taken together, our data suggest that the WD modulates expression of hippocampal microvascular genes associated with increases in BBB permeability.

We also performed bioinformatics analyses to identify transcription factors whose activity could be modulated by the WD in female brain microvessels. Among the transcription factors identified was CREB1 (cAMP Responsive Element Binding Protein 1). Interestingly, CREB1 has been shown to play an important role in regulating expression of genes involved in memory (both long-term and working memory), executive functions, and Alzheimer’s disease^45^. Another transcription factor identified was SP1 (specificity protein 1). Recently it has been shown that SP1 contributes to the pathogenesis of Alzheimer’s disease^46^ and cognitive dysfunction^47^. We also identified HIF1A (Hypoxia-inducible factor 1α) which plays an important role in cognitive function^48^. Furthermore, we identified nuclear ESR1 (estrogen receptor- alpha) and Ying Yang 1 (YYI) as transcription factor involved in the observed genomic effect in all three study groups. The expression of ESR1 can be modulated in brain endothelial cells and regulate their permeability^49^. Interaction of ESR1 with nutrients, such as vitamin D, regulates molecular pathways associated with Alzheimer’s disease development^50^. YY1 is a ubiquitously distributed transcription factor belonging to the GLI-Kruppel class of zinc finger proteins. YYI has been shown to induce or repress gene expression in inflammatory processes. It has been suggested that YY1 exerts inflammatory responses by interacting not only with DNA but also with long non-coding RNAs^51^. In microvascular endothelial cells, an injury such as hypoxia, modulates YY1 activity and increases adhesion of platelets to endothelial cells and expression of von Willebrand factor (VWF)^52^. This analysis suggests that chronic consumption of western-type diet could affect activity of different transcription factors in brain microvascular cells in females and result in the genomic changes observed.

**Figure 7:**
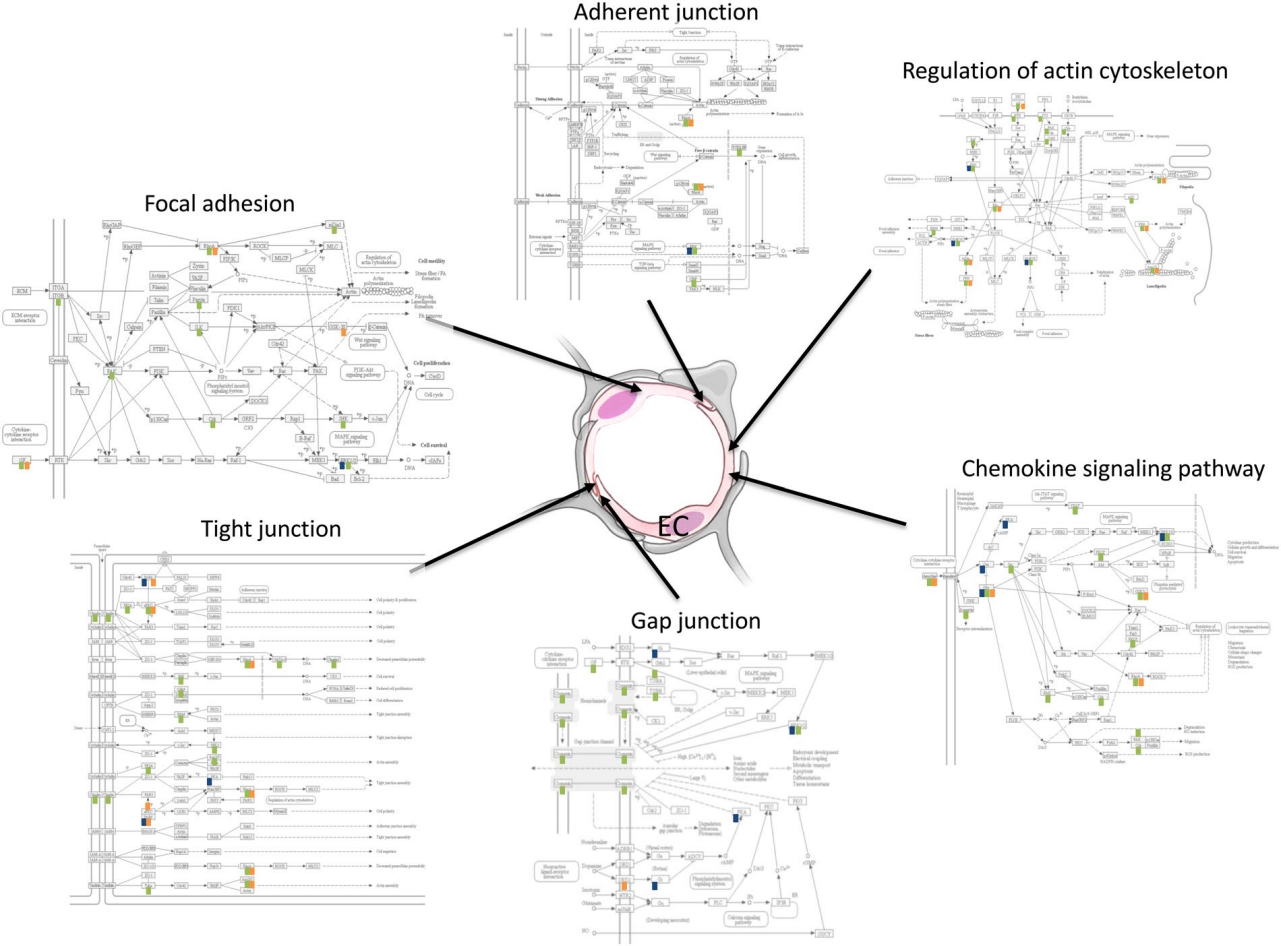
Schematic integration of cell-cell junction, cytoskeleton organization, chemotaxis, and focal adhesion pathways involved in the regulation of endothelial function in hippocampal brain microvessels. Pathways identified using KEGG database. Differentially expressed genes in WT WD compared to WT CD (blue); in LDL-R −/− CD compared to WT CD (green); and in LDL-R −/− WD compared to WT CD (orange).

Taken together, our bioinformatics analyses of protein coding genes identified important differential expression of genes whose activities can be modulated by the WD in female brain, and consequently expression of genes involved in regulation of endothelial function presenting interesting molecular mechanisms whereby consumption of the WD could impact cognitive function. Figure 7 provides a schematic summary of the key cellular processes differentially regulated by the WD in the female hippocampal microvasculature.

In addition to evaluating the impact of the WD on the expression of protein-coding genes, our studies also identified a previously unreported effect of the WD on the expression of protein non-coding RNAs in female hippocampal microvessels. These included microRNAs (miRNAs), small nucleolar RNAs (snoRNAs), and long non-coding RNAs (lncRNAs). Prior studies have suggest an important role for non-coding RNAs in the vasculature and in cognition. For example, Labouesse *et al*. showed that in adolescents a high fat diet (HFD) impacts global prefrontal cortex miRNA expression that affects neural function and axon guidance^17^. In addition, studies of aortas of atherosclerotic apolipoprotein E knock out (ApoE −/−) mice fed a high fat diet demonstrated global effects on expression of lncRNAs and miRNAs, particularly those having a role in metabolic and inflammation pathways^53^. Previous studies have also shown that changes in cortical transcriptome associated with Alzheimer’s disease are mediated by miRNAs^54^ and play an important role in higher order brain functions such as learning and memory^55^.

With respect to non-coding miRNA expression, we observed an up-regulation of miRNA340. Mir-340 expression is up-regulated after traumatic brain injury (TBI) and plays a role in TBI-induced memory and cognitive dysfunction^56^. We also observed increased mir-505 expression, and mir-505 levels have been shown to be elevated in cerebrospinal fluid (CSF) of patients with Alzheimer’s disease^57^. Also, elevated plasma levels of mir-505 in hypertensive patients are associated with impaired endothelial cell migration and tube formation^58^. Another identified differentially expressed miRNA was mir-329 that is present in serum of patients with mild cognitive impairment^59^. Vascular endothelial growth factor (VEGF) and tumor necrosis factor alpha (TNF-α) down-regulate the expression of mir-329 resulting in increased endothelial biomarker (CD146) expression and promoted angiogenic functions such as migration, cytoskeleton organization, formation of capillary networks and endothelial tubes^60^. This implies that WD activated mir-329 expression could impair cytoskeletal organization and migration, and potentially BBB permeability. Furthermore, we also observed that mir-181expression was increased, overexpression of which down-regulates proteins essential for memory (c-Fos and SIRT-1)^61^, indicating that increased expression of mir-181 in response to consumption of the WD could be associated with impaired memory. In contrast to the up-regulated miRNAs noted above, we also identified mir-485 as down-regulated. Interestingly, Lau *et al*. showed that mir-485 expression is decreased in late-onset Alzheimer’s disease patients^62^. Furthermore, down-regulation of mir-485 increases β-site APP cleaving enzyme 1 (BACE1), a protein involved in the formation of β-amyloid in AD patients^63,64^. Therefore, differential expression of miRNAs could be a mechanism for cognitive dysfunction.

In addition, analyses of predicted miRNA target genes identified several hundred for each dietary experimental group. These miRNA target genes are involved in different pathways such as those related to endothelial cell permeability and function, insulin signaling/resistance, and dementia. Interestingly, for the 3 study comparison groups we observed over 20 common pathways of the miRNA target genes, including the pathway involved in insulin signaling. This suggests that the WD, by modulating expression of miRNAs, can post-transcriptionally regulate genes involved in the regulation of the insulin pathway in hippocampal microvessels in females. Insulin dysregulation has been linked with cognitive dysfunction^65^ and endothelial cell dysfunction^66^. Other pathways identified from target genes of differentially expressed miRNAs are involved in Ras and Rap1 signaling, and small GTPases that regulates VE-cadherin-mediated cell-cell adhesions and cytoskeleton organization, and consequently, control of vascular permeability^67^. This suggests that the WD can modulate expression of miRNA in brain endothelial cells to increase endothelial permeability and consequently cognitive impairment. We also observed that miRNAs modulated by the WD can also regulate genes involved in cell-cell junction, focal adhesion or transendothelial leukocyte migration. Taken together, these results suggest that the Western diet affects the expression of miRNA, together with mRNA, with functional implications consistent with endothelial cell dysfunction and the pathogenesis of neurodegenerative and cognitive disorders.

Interestingly, our microarray analysis also identified changes in expression of other non-coding RNAs, such as snoRNAs. SnoRNAs are non-coding RNA molecules that play an important role in post-transcriptional modification of other RNAs, regulation of gene expression, and in stabilizing the genome^68^. We observed that the WD up-regulated expression of brain specific snoRNA AF357425 (MBII-48). Rogelj *et al*. have shown that in hippocampus, AF357425 is down-regulated following contextual fear memory consolidation^35^, memory that is reduced in patients with early-stage AD. Observed up-regulation of expression of this snoRNA suggests that AF357425 could have a role in impaired memory consolidation. We also identified WD associated changes in expression of snoRA2C and snoRA44. These two SnoRNAs have been identified as potentially involved in the development of Wilson-Turner Syndrome as suggested from MalaCards: The human disease database^36^. Wilson-Turner is a congenital X-linked condition characterized by intellectual disability associated with childhood-onset obesity^69^. This suggests that these two snoRNAs may play important roles in cognitive dysfunction associated with the WD. However, most of the snoRNAs we identified do not presently have any known function leaving open the possibility of additional important functional sequelae of the differential expression we observed in snoRNAs in our studies.

Our studies showed that the WD can also modulate expression of several long non-coding RNA (lncRNAs). LncRNAs are regulatory RNAs that function in transcriptional, post-transcriptional, and translational regulation of genes. As such, lncRNAs function in several aging-related processes, such as neuronal differentiation, apoptosis, and immune or stress responses^70,71^. We observed up-regulation in expression of lncRNA A630075F10Rik. An increase in A630075F10Rik expression has been shown to occur in macrophages treated with Serum amyloid P-component (SAP)^72^, and is associated with the development of cardiovascular disease^73^ and with impairment of cognition in centenarians^37^. We also observed increased expression of lncRNA SNHG7 (small nucleolar RNA host gene 7). SNHG7 expression is increased in granulin (GRN) peptide treated human neuroblastoma cells^38^. GRN is up-regulated in neurodegenerative diseases such as Alzheimer’s disease and multiple sclerosis and may function in neuro-inflammation^74–76^. The WD also increased lncRNA Gm12603 (WINCR1) expression in female hippocampal microvessels. Mullin *et al*. have shown that up-regulation of WINCR1 expression affects collective cell migration and collagen contraction^39^, suggesting that the WD- activated WINCR1 might affect endothelial cell migration and contractibility, and consequently potentially also endothelial permeability. In contrast to the above mentioned up-regulated lncRNAs, we identified lncRNA 2900097C17Rik (NORAD), an inhibitory regulator of inflammation in macrophages^40^, as down-regulated. This implies that a decrease in the expression of 2900097C17Rik could increase inflammation in hippocampal microvessels. In summary, these findings suggest for the first time that the WD modulates expression of non-coding RNAs (miRNAs, snoRNAs and lncRNAs) in the female hippocampal microvasculature that may play an important role in diet-associated brain microvascular disease and dementia.

Our study has a few limitations. It is a pilot study and as such our results need further follow-up including considering hippocampal subsection analysis, and the possibility of obtaining and correlating the findings with estrous cycle data (we did perform estradiol levels finding no differences between study groups, data not shown). We chose to perform our studies in the LDL-R −/− phenotype and recognize that the findings may differ in other murine models of hyperlipidemia. In addition, although our previous studies indicate that the hippocampal impact of the WD is lipotoxic in nature, we cannot rule out glycemic injury associated with the WD and further studies are needed to make this distinction. Furthermore, future functional analyses for cognition and BBB permeability would provide a functional correlate to the molecular pathways identified in this pilot study.

**Figure 8:**
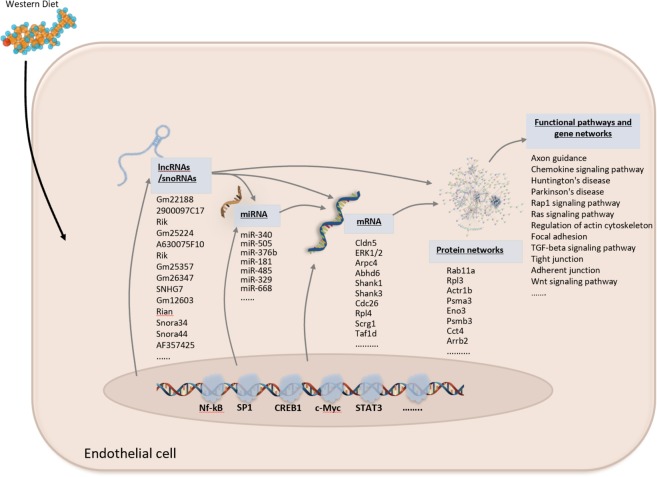
Summary schematic of the multilevel method of molecular regulation of hippocampal microvascular gene expression in females in response to the Western diet. The schematic summarizes the effect of the Western diet on modulating the expression of protein coding and protein non-coding genes (microRNAs, long-non coding RNAs and SnoRNAs), as well as their interaction with other RNAs and proteins, to exert post-transcriptional functional regulation in female hippocampal microvessels.

In conclusion, this study investigated the molecular mechanisms of differential gene expression induced by the Western diet in the hippocampal microvasculature of female mice. We used a genome-wide microarray and bioinformatics analysis of laser-captured microvessels to identify differential gene expression, gene networks and pathways, transcription factors, and non-protein coding miRNAs. We identified a panel of differentially expressed genes, miRNAs, lncRNAs and snoRNAs in response to the WD. Our study is significant because to our knowledge it is the first to examine the genomic effect of the WD on isolated brain microvascular endothelium in females. Such genes are involved in several important functional pathways and protein networks, including BBB permeability and vascular integrity. In addition, we herein propose an integrated multilevel molecular regulation of hippocampal microvessels in response to the WD in females, summarized in the schematic in Fig. 7. The genomic cascade demonstrates effects that span the genomic spectrum. Taken together, our nutrigenomics data show the complexity of the molecular effect of the WD on brain hippocampal microvascular endothelium that proceeds via simultaneous regulation of expression of protein-coding and non-coding genes. Furthermore, as shown in Fig. 8 we comprehensively integrate the data and assess the impact of diet/genotype in relation to the key biological processes and functions affected; namely, focal adhesion, tight junctions, adherent junctions, gap junction, cytoskeletal actin regulation and chemokine signaling pathways. In addition, the differentially expressed genes, pathways, and transcriptional regulatory mechanisms identified in our study may provide potential drug targets or biomarkers for addressing diet-related microvascular endothelial injury with relevance to cognitive impairment and vascular dementia in females. Furthermore, in future studies it could be interesting to look at the genomic responses we observed as one of the mechanisms that may help explain the epidemiological differences between males and females in dementia occurrence and severity.

## Supplementary information

supplemental figures and tables
