# Supplementary material for: The Western Diet Regulates Hippocampal Microvascular Gene Expression: An Integrated Genomic Analyses in Female Mice
Source: Sci Rep. 2019 Dec 13;9:19058. doi: 10.1038/s41598-019-55533-9 (PMC6911042; doi:10.1038/s41598-019-55533-9)
Supplement: Supplementary file 1 — supplemental figures and tables [file 41598_2019_55533_MOESM1_ESM.pdf]

The Western Diet Regulates Hippocampal Microvascular Gene Expression: An Integrated Genomic Analyses in Female Mice

Saivageethi Nuthikattu[1], Dragan Milenkovic[1,2], John Rutledge[1], Amparo Villablanca[1]

[1] Division of Cardiovascular Medicine, University of California, Davis; Davis, California

[2] Université Clermont Auvergne, INRA, UNH, CRNH Auvergne, F-63000 Clermont-Ferrand, France

Supplement Figure S1: Mean body weight of wild type (WT) and LDL-R -/- mice pre- and post-feeding with the control (CD) and western (WD) diets. Line graph shows mean weight (grams) of wild type (WT) and LDL-R -/- mice before feeding (at age 5 weeks, solid lines) and after feeding (at age 13 weeks, dashed lines) with the control (CD) and western (WD) diets. Weight increased for mice in all the 4 groups (WT CD, WT WD, LDL-R -/- CD and LDL-R -/- WD) post the diet when compared to pre-diet intervention ($p<0.05$ for all pre/post comparisons).

Supplement Figure S2: Volcano plots of gene expression changes in hippocampal microvessels. The transcriptome of: A) microvessels from western diet (WD) fed C57BL/6J (WT) compared to microvessels from control diet (CD) fed WT mice; B) microvessels from CD fed LDL-R -/- mice compared to microvessels from CD fed WT mice; and C) microvessels from WD fed LDL-R -/- mice compared to microvessels from CD fed WT mice. The x-axis specifies the fold-changes (FC) and the y-axis specifies the negative logarithm to the base 10 of the p-values. Red vertical and horizontal lines reflect the filtering criteria (FC = fold change, ±2.0 and p-value = 0.05). Red and green dots represent probe sets for transcripts expressed at significantly higher or lower levels than control mice (CD fed WT mice), respectively.

Supplement Figure S3: Gene expression by qRT-PCR of genes identified by microarray analysis in hippocampal microvessels. Six protein coding genes (Atxn7l1, Slc17a5, Ndufa4, Rpl3, Psmb4 and Bmpr2) and 3 non-coding genes (ScaRNA3B, Mir340and Mir505) were tested by qRT-PCR in hippocampal microvessels isolated from wild type (WT) and LDL-R -/- mice fed with control diet (CD) and western diet (WD) and showed the same trend in gene expression as microarray. Protein coding gene expression was normalized to glyceraldehyde-

3-phosphate dehydrogenase (GAPDH), and non-coding gene expression was normalized to small nucleolar RNA 68 (SNORNA68). Expression levels were expressed as log2 fold change (*$p \leq 0.05$ for WT WD, LDL-R -/- CD, and LDL-R -/- WD when compared to WT CD).

Supplement Figure S4. Predicted protein–protein interactions generated following STRING database analysis of significantly differentially expressed genes. The network nodes represent the proteins encoded by the differentially expressed genes and lines represent protein-protein interactions. A) Western diet (WD) fed C57BL/6J (WT) mice compared to control diet (CD) fed WT mice; B) CD fed LDL-R -/- mice compared to CD fed WT mice; C) WD fed LDL-R -/- mice compared to CD fed WT mice.

Supplement Figure S5. Venn diagram of the top 30 transcription factors affected by diet and genotype in hippocampal microvascular endothelium. Venn diagram shows 16 transcription factors in common for WD fed WT mice, CD fed LDL-R -/- mice, and WD fed LDL-R - /- mice, when compared to CD fed WT mice.

Supplement Figure S6A. Venn diagrams representing the number of differentially expressed (DE) genes, compared to miRNA target genes, affected by diet and genotype in hippocampal microvessels. Venn diagrams of differentially expressed (DE) genes and miRNA target genes showing 10, 123, and 46 genes in common for WD fed WT mice, CD fed LDL-R -/- mice, and WD fed LDL-R -/- mice, respectively, when compared to CD fed WT mice. Genes targeted by miRNAs were identified using the miRWalk database.

Supplement Figure S6B. Venn diagrams representing the number of differentially expressed (DE) gene pathways, compared to miRNA target gene pathways, affected by

diet and genotype in hippocampal microvessels. Venn diagrams of differentially expressed (DE) gene pathways and miRNA target gene pathways showing 34, 84, and 52 pathways in common for WD fed WT mice, CD fed LDL-R -/- mice, and WD fed LDL-R -/- mice, respectively, when compared to CD fed WT mice. Pathways of DE genes and miRNA target genes were identified using the KEGG database and Genetrail2.

Supplement Figure 1: Mean body weight of wild type (WT) and LDL-R -/- mice pre- and post-feeding with the control (CD) and western (WD) diets.

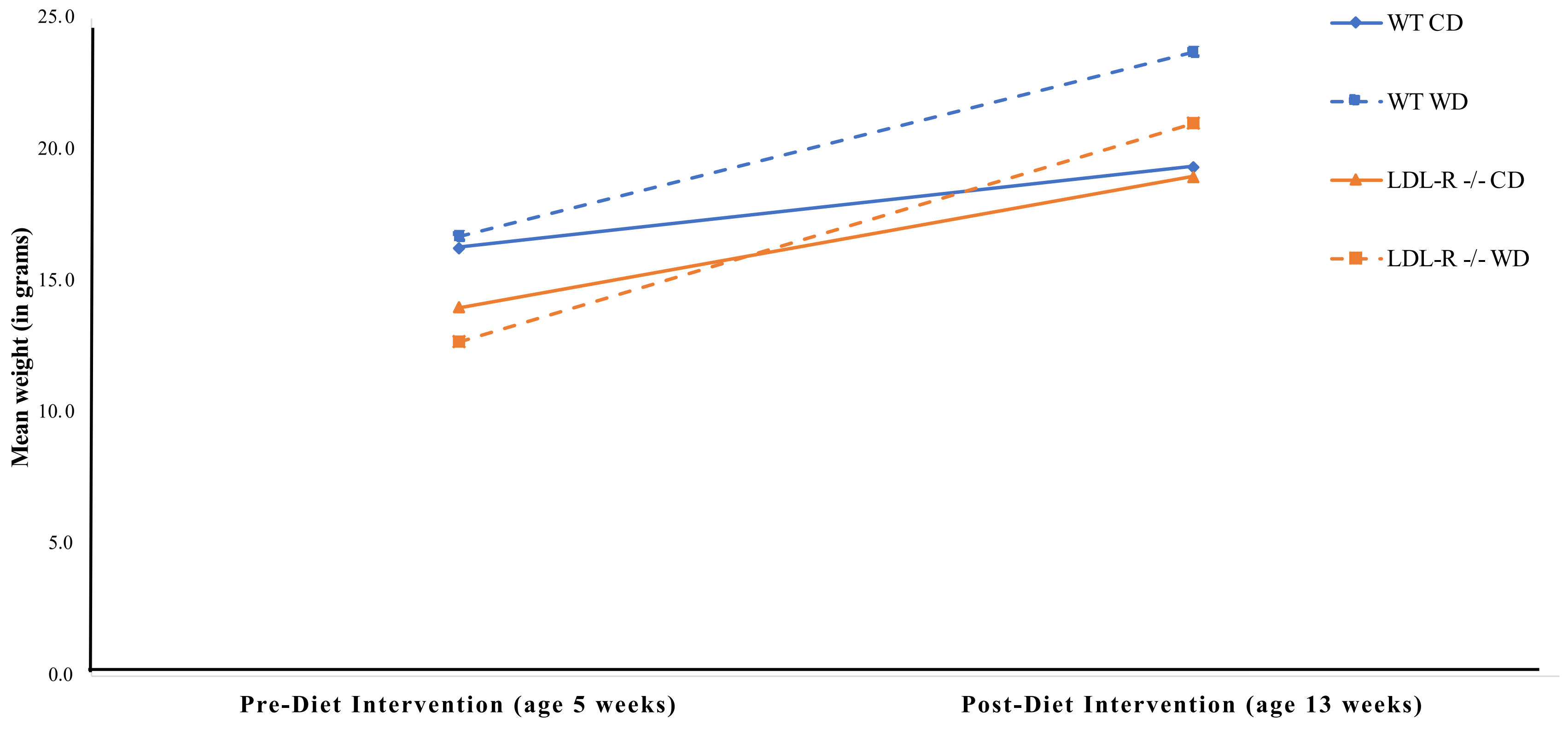

Supplement Figure 2: Volcano plots of gene expression changes in hippocampal microvessels.

Supplement Figure 2 (A)

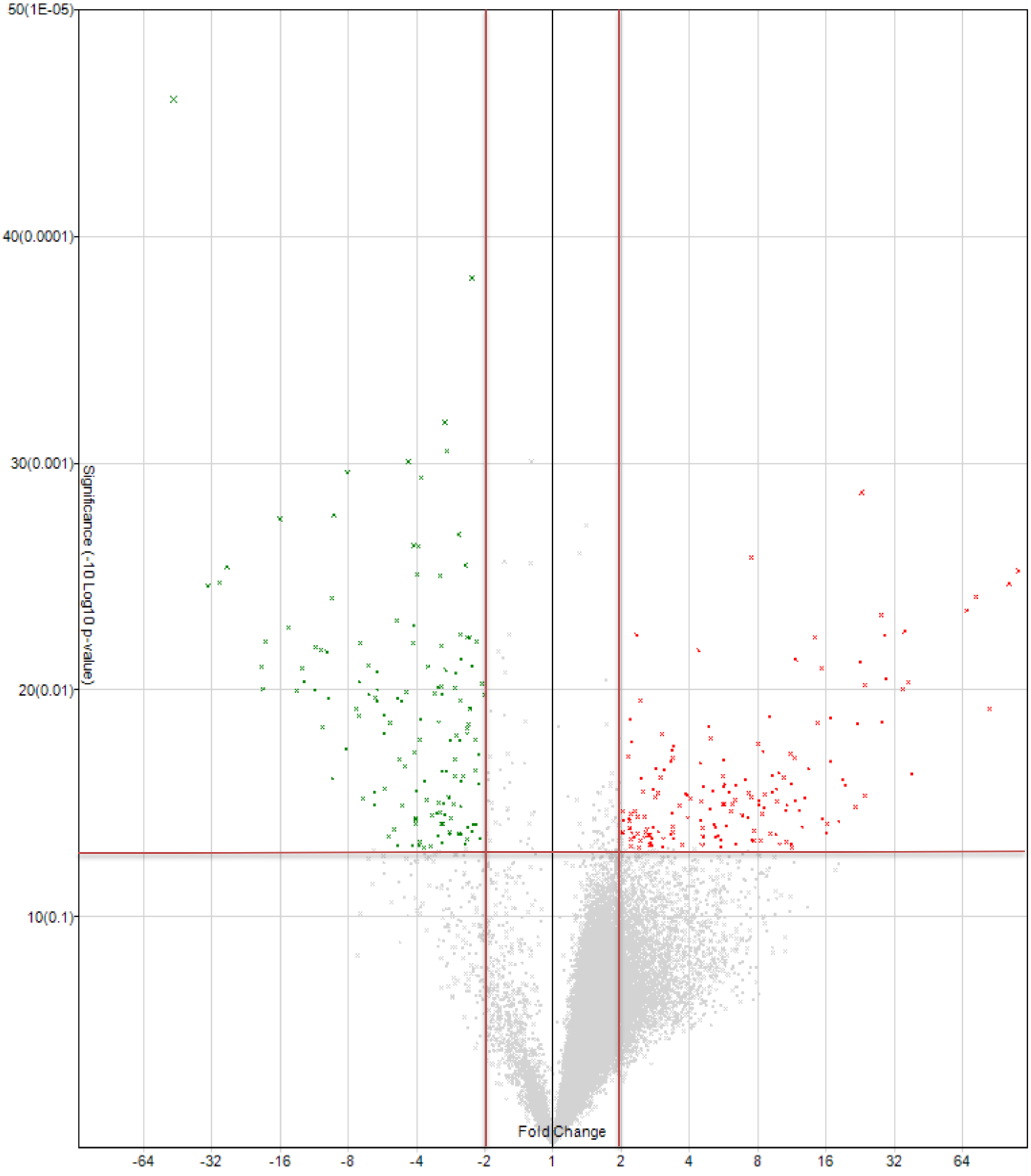

WT WD vs WT CD

Supplement Figure 2 (B)

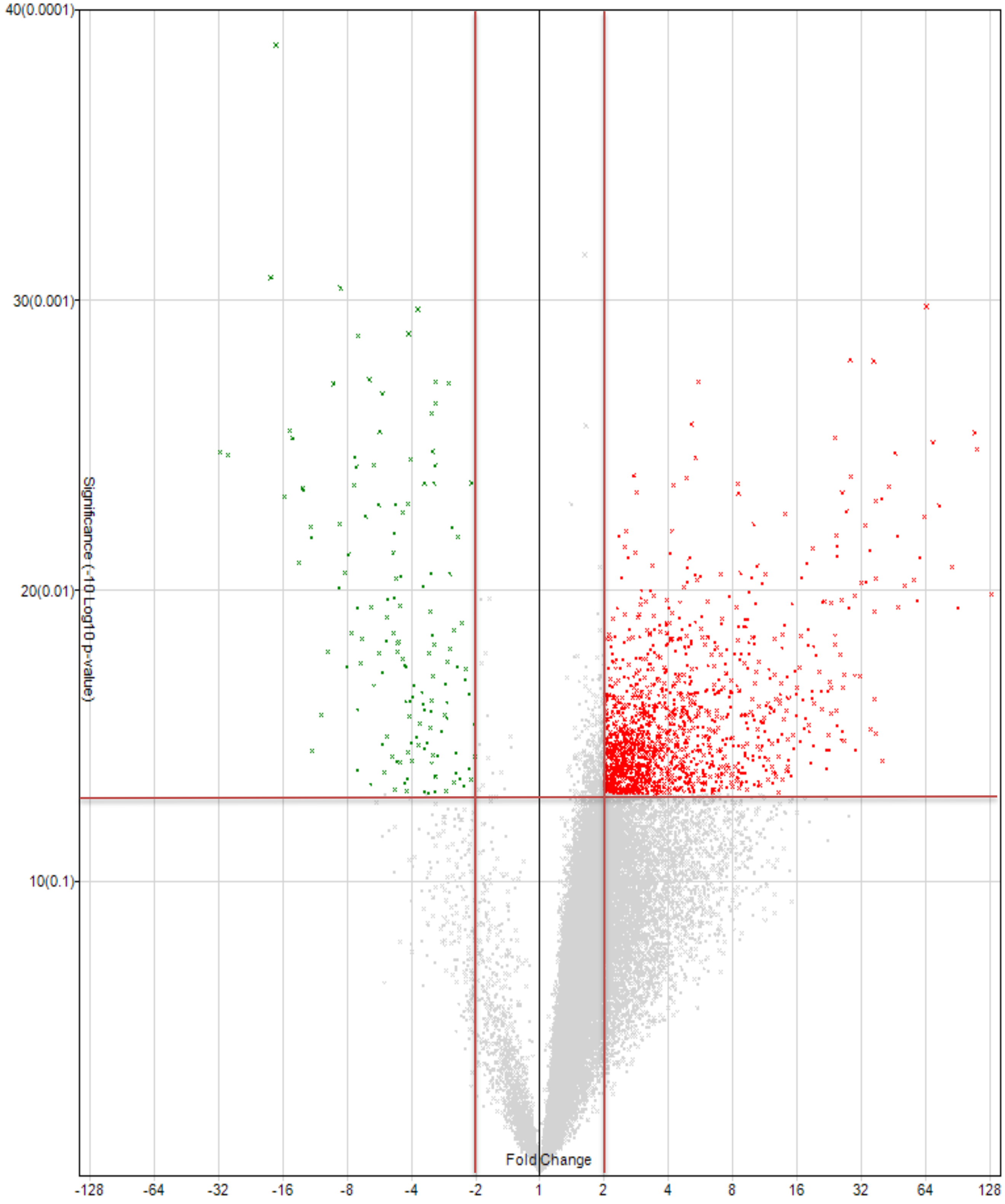

LDL-R -/- CD vs WT CD

Supplement Figure 2 (C)

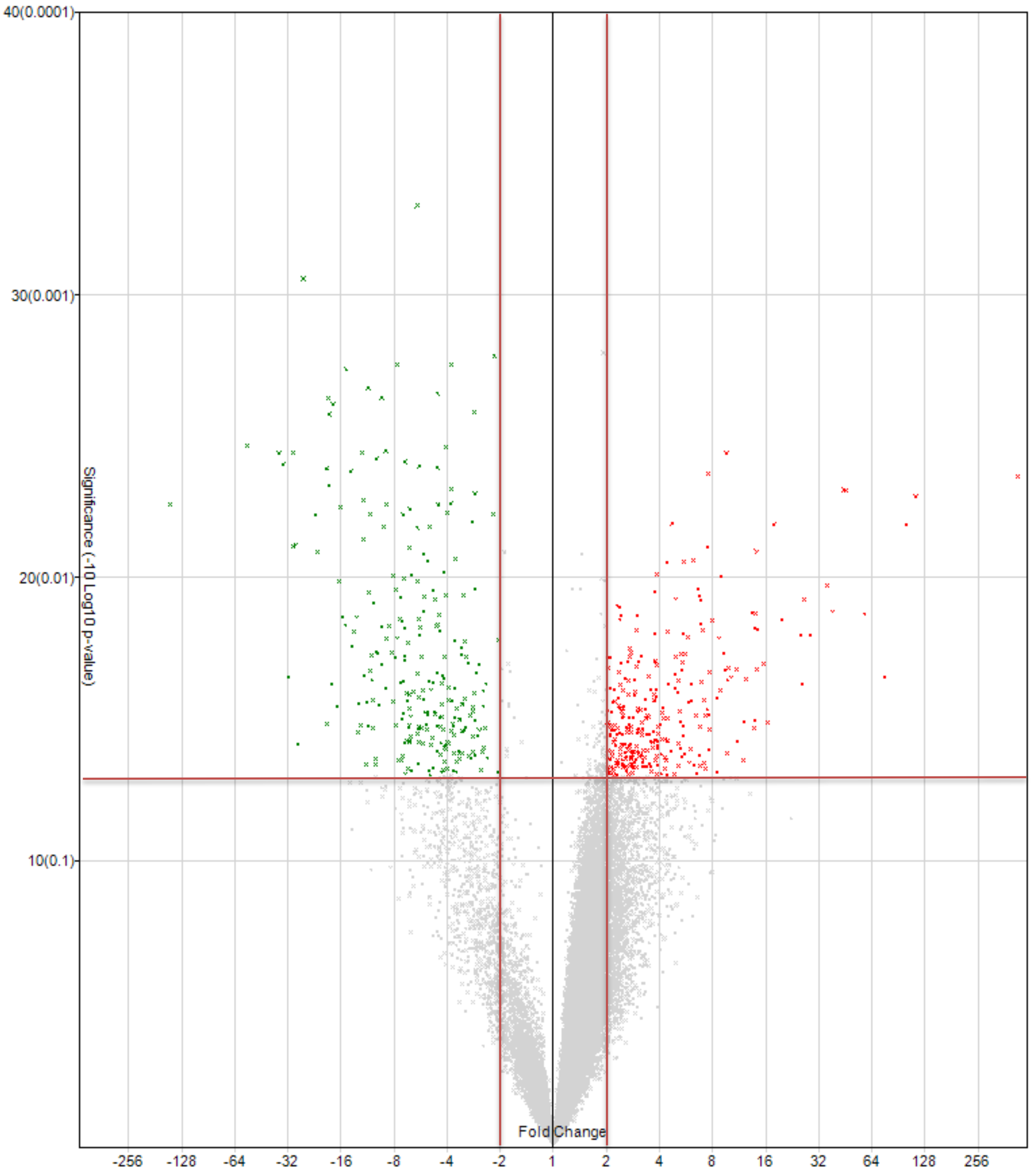

LDL-R -/- WD vs WT CD

Supplement Figure 3. Gene expression by qRT-PCR of genes identified by microarray analysis in hippocampal microvessels.

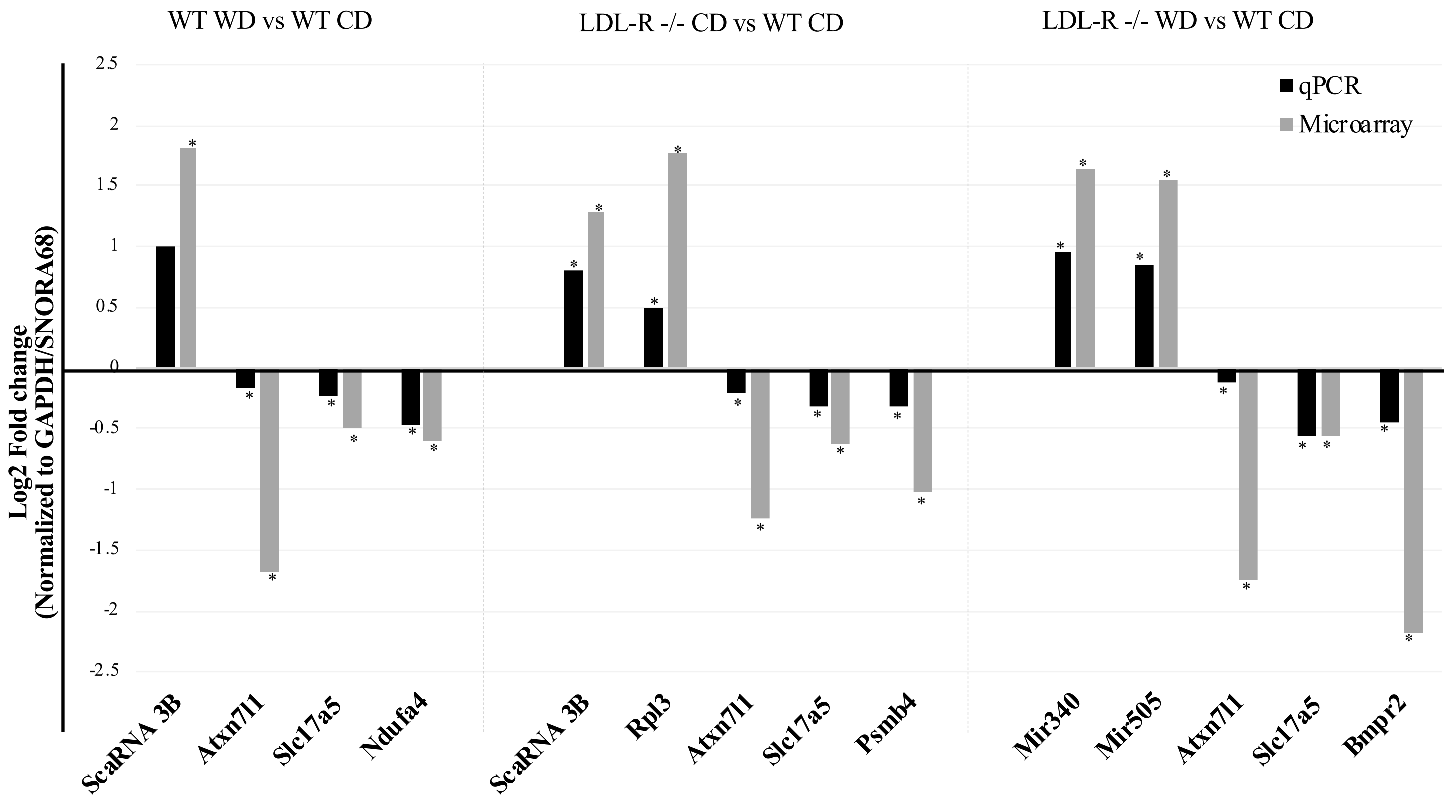

Supplement Figure 4. Predicted protein–protein interactions generated following STRING database analysis of significantly differentially expressed genes.

Supplement Figure 4 (A)

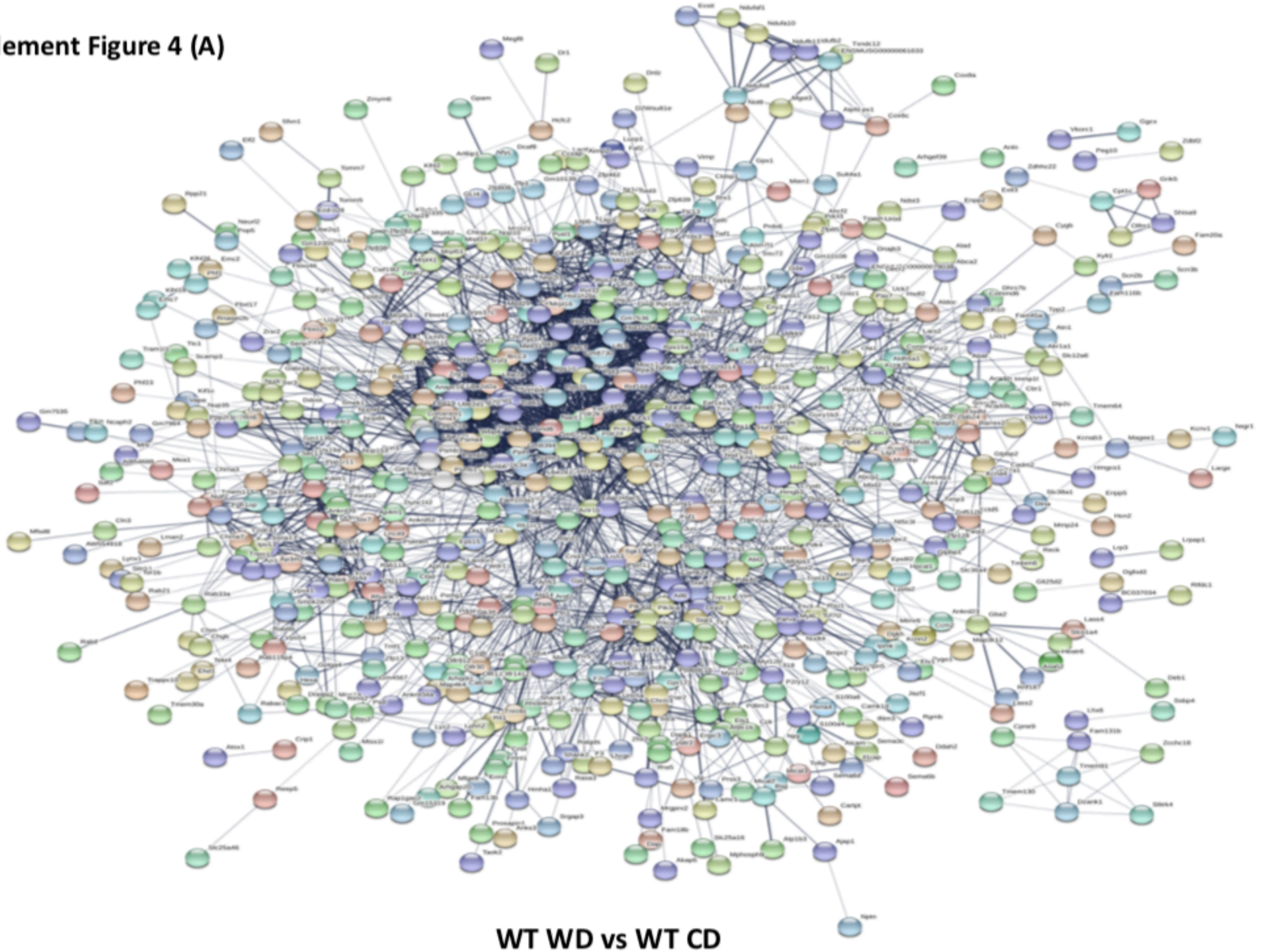

WT WD vs WT CD

Supplement Figure 4 (B)

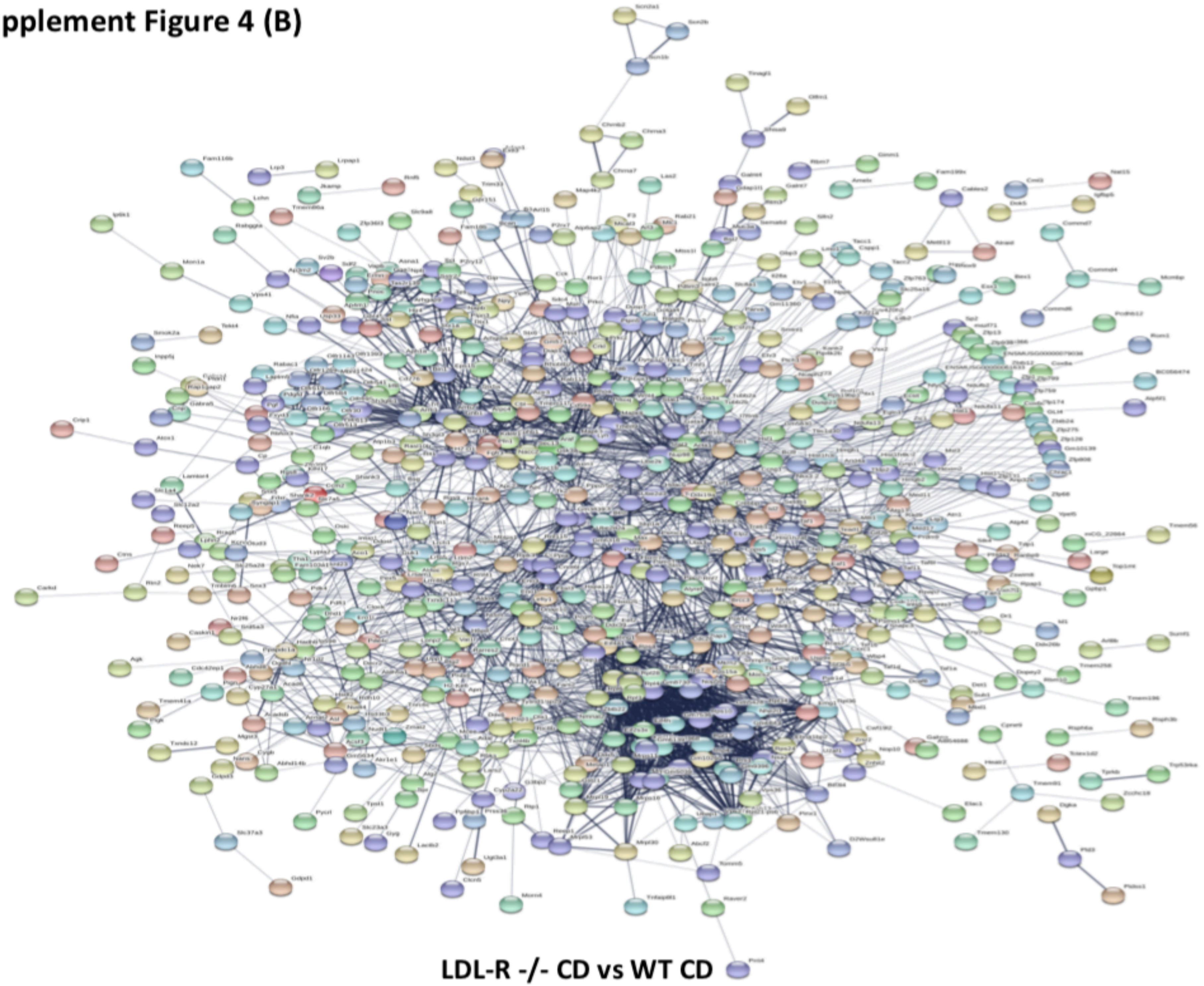

LDL-R -/- CD vs WT CD

Supplement Figure 4 (C)

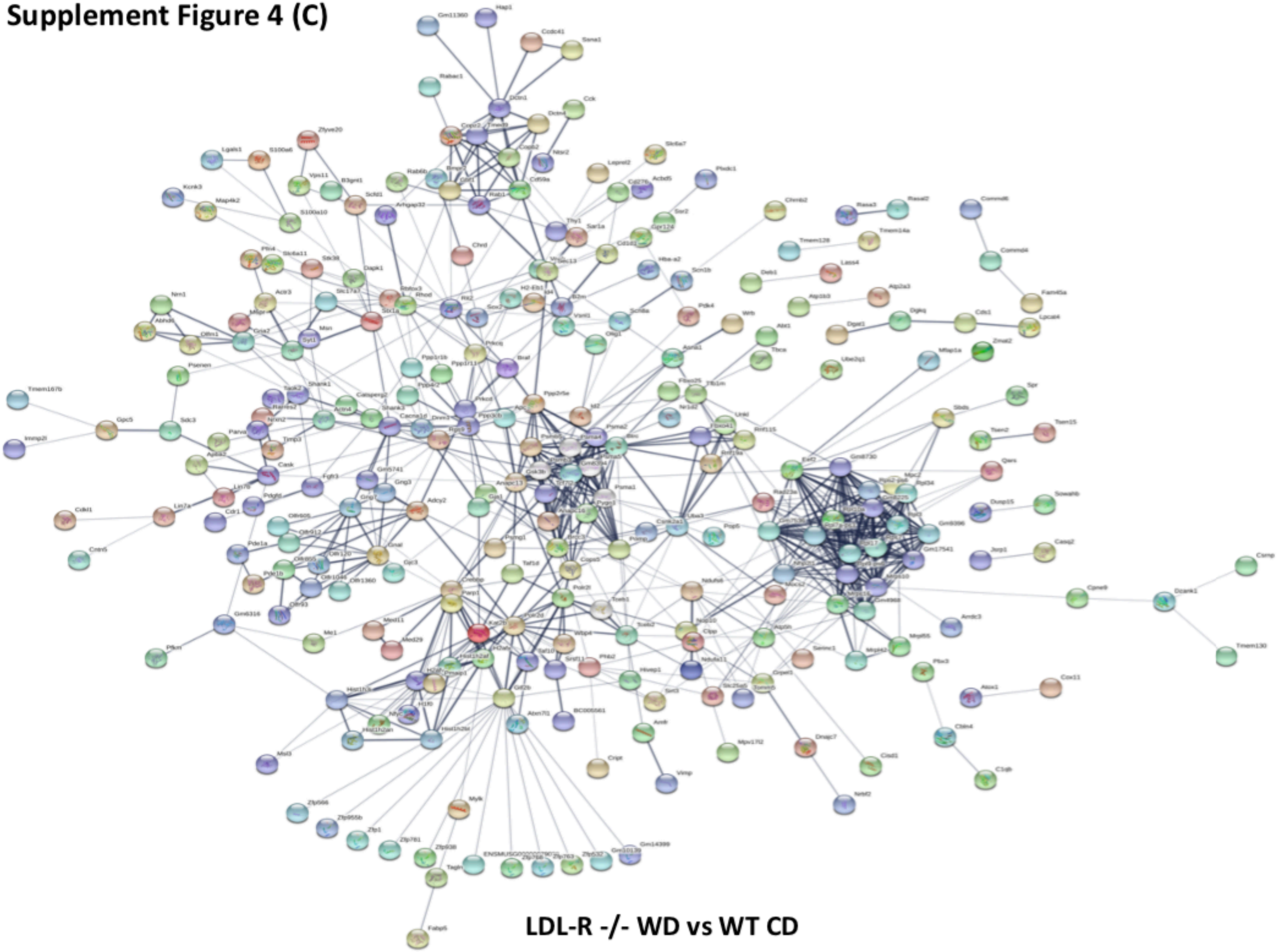

LDL-R -/- WD vs WT CD

Supplement Figure 5. Venn diagram of the top 30 transcription factors affected by diet and genotype in hippocampal microvascular endothelium.

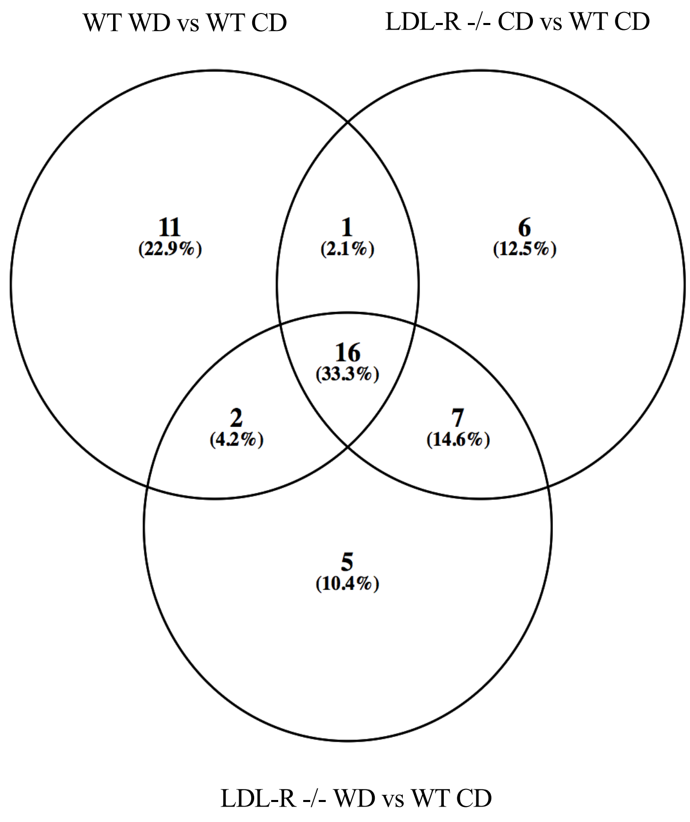

Supplement Figure 6A. Venn diagrams representing the number of differentially expressed (DE) genes, compared to miRNA target genes, affected by diet and genotype in hippocampal microvessels.

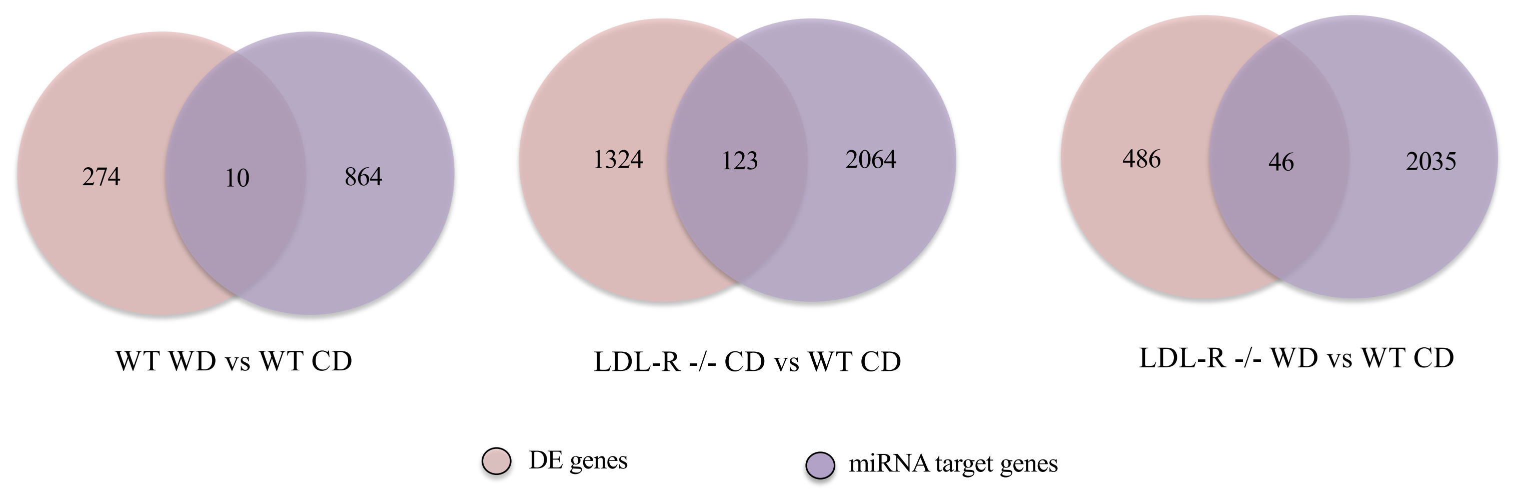

Supplement Figure 6B. Venn diagrams representing the number of differentially expressed (DE) gene pathways compared to miRNA target gene pathways affected by diet and genotype in hippocampal microvessels.

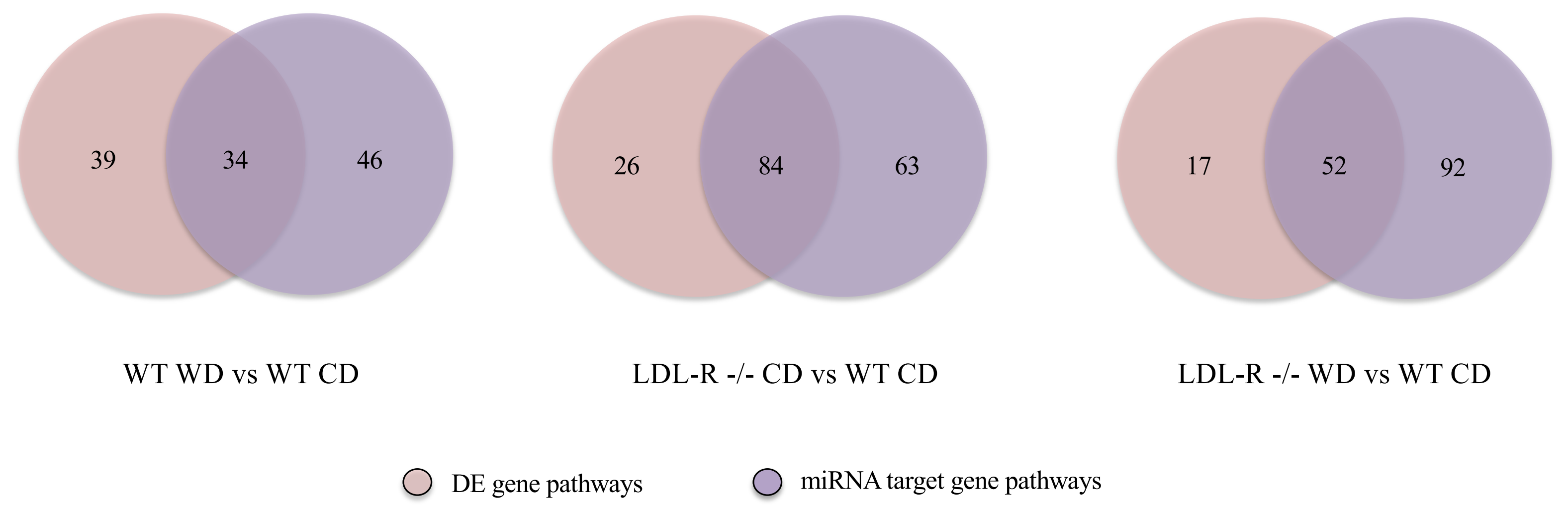

Table S1. Primer sequences for genes tested by qRT-PCR were prepared by Primer3 software using Affymetrix transcript ID sequences.

Gene	Primer Sequence (5′-3′)
GAPDH	Sense-GCAACAGGGTGGTGGACCT
	Antisense GGATAGGGCCTCTCTTGCTCA
SnoRNA68	Sense-TAGTGGTGCACACAGCAAAG
	Antisense -AGAGCCTACTGATTCACTCTGG
ScaRNA3b	Sense-GAAAATGCCTTTGTTTGCAG
	Antisense -CGATCAGACTCAGCCAGCTA
Atxn7l1	Sense-GGCGTGGAAGACATTAGGAA
	Antisense-TACTGTGTTGGCGTCTCCTG
Slc17a5	Sense-CCCCTGTTACAGCCACTGTT
	Antisense-TTTCCCTCGTGCTTGTCTCT
Ndufa4	Sense-GGCTGATGTAAGGCCATGTT
	Antisense-TAAAGCTGTGTGCTGCCATC
Rpl3	Sense-GATAATGTGAGACCCTGGAACG
	Antisense-AGAAGGCAGGCAGGCAAG
Psmb4	Sense-TGAAGTGTTCGCTGAGTTGG
	Antisense-TCTAGCTTTTGGGAGGCAGA
Mir340	Sense-CAATTGTACTTGGTGTGATTATAAAGC
	Antisense-AGTAACTGAGACGGATCCCACA
Mir505	Sense -GTGGGGGAGCCAGGAAGTAT
	Antisense -TGATACTCCAGAGAGAAAACCAG
Bmpr2	Sense-AGTTGTGTGACGGAGCAGTG
	Antisense-TCAGCGTTCATAGTGGCATC

Table S2 (A). Plasma lipid levels of wildtype (WT) and LDL-R -/- mice fed with control (CD) and western (WD) diet.

Plasma Lipids (Mean±SEM)	WT-CD	WT-WD	LDL-R -/- CD	LDL-R -/- WD
**TC (mg/dL)**	73.9±10.0	119.8±7.1*	225.7±24.0*	1259.6±59.5*
**TG (mg/dL)**	79.3±20.0	50.7±5.1	125.9±26.6	537.5±72.2*
**LDL (mg/dl)**	0.93±2.0	14.5±0.6*	113.0±17.2*	1038.6±39.7*
**HDL (mg/dL)**	57.1±8.4	95.1±5.8*	87.5±11.7	113.5±14.3*

** p <0.05 for TC, TG, LDL and HDL compared to CD fed WT mice*

Table S2 (B). Plasma Glucose and Insulin levels of wildtype (WT) and LDL-R -/- mice fed with control (CD) and Western (WD) diet.

Mean±SEM	WT-CD	WT-WD	LDL-R -/- CD	LDL-R -/- WD
**Glucose (mg/dL)**	172.1±50.4	430.4±34.6**	303.0±23.1**	424.1±27.9**
**Insulin (pg/mL)**	102.9±11.7	132.6±46.8	249.6±34.3*	179±54.6*

**   p <0.05 for insulin in LDL-R -/- groups compared to WT groups*

*** p <0.05 for glucose compared to CD fed WT mice*

Table S3: Differentially expressed genes in Western diet (WD) fed WT mice when compared to control diet (CD) fed WT mice.

Gene Symbol	Gene Description	Fold Change
DQ267102	snoRNA DQ267102	112.18
		102.31
		83.64
Syne1	spectrin repeat containing, nuclear envelope 1	73.27
Scarna3b; Mir1843a	small Cajal body-specific RNA 3B; microRNA 1843a	66.49
Syne1	spectrin repeat containing, nuclear envelope 1	38.04
Uck2	uridine-cytidine kinase 2	36.77
1110038B12Rik; Gm25744	RIKEN cDNA 1110038B12 gene [Source:MGI Symbol;Acc:MGI:1916013]; predicted gene, 25744 [Source:MGI Symbol;Acc:MGI:5455521]	35.4
Gm23713; Gm22615	predicted gene, 23713 [Source:MGI Symbol;Acc:MGI:5453490]; predicted gene, 22615 [Source:MGI Symbol;Acc:MGI:5452392]	34.89
Gm23713; Gm22615	predicted gene, 23713 [Source:MGI Symbol;Acc:MGI:5453490]; predicted gene, 22615 [Source:MGI Symbol;Acc:MGI:5452392]	34.89
Gm24056	predicted gene, 24056 [Source:MGI Symbol;Acc:MGI:5453833]	29.27
Syne1	spectrin repeat containing, nuclear envelope 1	28.95
Syne1	spectrin repeat containing, nuclear envelope 1	28.17
Gm25186	predicted gene, 25186 [Source:MGI Symbol;Acc:MGI:5454963]	27.96
Eif4a2	eukaryotic translation initiation factor 4A2	23.71
Pmch	pro-melanin-concentrating hormone	23.65
Gm24009	predicted gene, 24009 [Source:MGI Symbol;Acc:MGI:5453786]	22.97
Syne1	spectrin repeat containing, nuclear envelope 1	22.52
Gm24217	predicted gene, 24217 [Source:MGI Symbol;Acc:MGI:5453994]	22.01
Syne1	spectrin repeat containing, nuclear envelope 1	21.44
Mapk3	mitogen-activated protein kinase 3	19.34
2610524H06Rik	RIKEN cDNA 2610524H06 gene	18.89
Snora44	small nucleolar RNA, H/ACA box 44	18.16
Gm25604	predicted gene, 25604 [Source:MGI Symbol;Acc:MGI:5455381]	16.78
Ap1s1	adaptor protein complex AP-1, sigma 1	16.73

mt-Tv	mitochondrially encoded tRNA valine [Source:MGI Symbol;Acc:MGI:102472]	16.11
Vezf1	vascular endothelial zinc finger 1	15.94
Slc35e3	solute carrier family 35, member E3	15.46
Cnnm2	cyclin M2	15.24
Mir679	microRNA 679	14.7
Tmem64	transmembrane protein 64	14.23
Mag	myelin-associated glycoprotein	13.39
Gm12226	predicted pseudogene 12226 [Source:MGI Symbol;Acc:MGI:3649742]	12.86
Syne1	spectrin repeat containing, nuclear envelope 1	12.56
Tmem9b	TMEM9 domain family, member B	12.12
Gm25357	predicted gene, 25357 [Source:MGI Symbol;Acc:MGI:5455134]	11.71
Gm25559	predicted gene, 25559 [Source:MGI Symbol;Acc:MGI:5455336]	11.7
Lhcgr	luteinizing hormone/choriogonadotropin receptor	11.62
Gm22043	predicted gene, 22043 [Source:MGI Symbol;Acc:MGI:5451820]	11.27
Nbl1	neuroblastoma, suppression of tumorigenicity 1	11.24
Syne1	spectrin repeat containing, nuclear envelope 1	11.17
Nme6	NME/NM23 nucleoside diphosphate kinase 6	11.17
Gm24095	predicted gene, 24095 [Source:MGI Symbol;Acc:MGI:5453872]	10.74
Rps19-ps3	ribosomal protein S19, pseudogene 3	10.71
Gm22579; Gm24539	predicted gene, 22579 [Source:MGI Symbol;Acc:MGI:5452356]; predicted gene, 24539 [Source:MGI Symbol;Acc:MGI:5454316]	10.62
Taf1d	TATA box binding protein (Tbp)-associated factor, RNA polymerase I, D	10.62
Syne1	spectrin repeat containing, nuclear envelope 1	10.39
Elavl1	ELAV (embryonic lethal, abnormal vision)-like 1 (Hu antigen R)	10.01
		9.9
Lims2	LIM and senescent cell antigen like domains 2	9.72
Nicn1	nicolin 1	9.66
Sgpp2	sphingosine-1-phosphate phosphotase 2	9.62
mt-Tc	mitochondrially encoded tRNA cysteine [Source:MGI Symbol;Acc:MGI:102490]	9.27
		9.27

Mir204	microRNA 204	9.26
Dusp18	dual specificity phosphatase 18	9.12
Gm24103	predicted gene, 24103 [Source:MGI Symbol;Acc:MGI:5453880]	8.97
Pfdn1	prefoldin 1	8.58
Gm5830	predicted pseudogene 5830 [Source:MGI Symbol;Acc:MGI:3647375]	8.49
Gm15319	predicted gene 15319	8.43
Ldb1	LIM domain binding 1	8.37
Hspa12a	heat shock protein 12A	8.26
Kcnip1	Kv channel-interacting protein 1	8.08
Plekho1	pleckstrin homology domain containing, family O member 1	8.06
Syne1	spectrin repeat containing, nuclear envelope 1	8.04
Mpv17	MpV17 mitochondrial inner membrane protein	7.66
Wfs1	Wolfram syndrome 1 homolog (human)	7.65
Mfap1a	microfibrillar-associated protein 1A	7.53
Syne1	spectrin repeat containing, nuclear envelope 1	7.51
Gm25788	predicted gene, 25788 [Source:MGI Symbol;Acc:MGI:5455565]	7.48
		7.27
Epas1	endothelial PAS domain protein 1	7.23
		7.05
		6.84
Supt3	suppressor of Ty 3	6.38
Anp32b	acidic (leucine-rich) nuclear phosphoprotein 32 family, member B	6.38
Gtf2h3	general transcription factor IIH, polypeptide 3	6.36
Mir1193	microRNA 1193	6.17
Tac2	tachykinin 2	6.07
Apoa1bp	apolipoprotein A-I binding protein	5.95
		5.81
		5.7
7420700N18Rik	RIKEN cDNA 7420700N18 gene	5.69
Tma7	translational machinery associated 7 homolog (S. cerevisiae)	5.67
Nup35	nucleoporin 35	5.66
Clec2d	C-type lectin domain family 2, member d	5.62
Zbtb38; E030011O05Rik	zinc finger and BTB domain containing 38; RIKEN cDNA E030011O05 gene	5.58
Zbtb39	zinc finger and BTB domain containing 39	5.52
Prkaca	protein kinase, cAMP dependent, catalytic, alpha	5.47

Gm12250	predicted gene 12250	5.37
Ighv2-6-8	immunoglobulin heavy variable 2-6-8	5.21
Syne1	spectrin repeat containing, nuclear envelope 1	5.18
Syne1	spectrin repeat containing, nuclear envelope 1	5.15
Cmtm8	CKLF-like MARVEL transmembrane domain containing 8	5.06
Snord42b	small nucleolar RNA, C/D box 42B	4.96
		4.92
Gm14295; Gm38519	predicted gene 14295; predicted gene, 38519	4.85
mt-Tg	mitochondrially encoded tRNA glycine [Source:MGI Symbol;Acc:MGI:102486]	4.6
Rps15a-ps6	ribosomal protein S15A, pseudogene 6	4.59
Gm25519	predicted gene, 25519 [Source:MGI Symbol;Acc:MGI:5455296]	4.57
Mnat1	menage a trois 1	4.56
Rmrp	RNA component of mitochondrial RNAase P	4.5
LOC101055672	nuclear body protein SP140-like	4.47
Gm25224	predicted gene, 25224 [Source:MGI Symbol;Acc:MGI:5455001]	4.46
Mir5099	microRNA 5099	4.39
Rabggta	Rab geranylgeranyl transferase, a subunit	4.03
Serac1	serine active site containing 1	3.93
		3.89
Syne1	spectrin repeat containing, nuclear envelope 1	3.82
Zfp458	zinc finger protein 458	3.73
Zfp867	zinc finger protein 867	3.61
Mir28c	microRNA 28c	3.4
		3.39
Gm5316	predicted gene 5316 [Source:MGI Symbol;Acc:MGI:3645576]	3.38
Anln	anillin, actin binding protein	3.38
		3.37
mt-Tn	mitochondrially encoded tRNA asparagine [Source:MGI Symbol;Acc:MGI:102479]	3.35
Gm12207	predicted gene 12207 [Source:MGI Symbol;Acc:MGI:3702399]	3.33
Bvht	braveheart long non-coding RNA	3.32
Polr2d	polymerase (RNA) II (DNA directed) polypeptide D	3.29
Gm11467	predicted gene 11467 [Source:MGI Symbol;Acc:MGI:3650520]	3.08
Gm4968	predicted gene 4968 [Source:MGI Symbol;Acc:MGI:3647516]	3.07

Gm22188	predicted gene, 22188 [Source:MGI Symbol;Acc:MGI:5451965]	3.05
Ogfod1	2-oxoglutarate and iron-dependent oxygenase domain containing 1	3.02
Gm23613	predicted gene, 23613 [Source:MGI Symbol;Acc:MGI:5453390]	2.97
Cxxc5	CXXC finger 5	2.91
Stxbp3-ps	syntaxin-binding protein 3, pseudogene	2.9
Pcp4; Igsf5	Purkinje cell protein 4; immunoglobulin superfamily, member 5	2.85
Gm36490	predicted gene, 36490	2.82
mt-Tq	mitochondrially encoded tRNA glutamine [Source:MGI Symbol;Acc:MGI:102477]	2.76
Snora34; Mir1291	small nucleolar RNA, H/ACA box 34; microRNA 1291	2.75
		2.73
Abhd3	abhydrolase domain containing 3	2.72
Mnd1	meiotic nuclear divisions 1 homolog (S. cerevisiae)	2.7
Mir669f	microRNA 669f	2.69
		2.67
		2.66
Gng4	guanine nucleotide binding protein (G protein), gamma 4	2.63
Tas2r143	taste receptor, type 2, member 143	2.6
Gm15446	predicted gene 15446	2.55
		2.54
		2.54
		2.54
Gm29721; Gm2808	predicted gene, 29721; predicted gene 2808	2.49
		2.43
7167; Gm29964; Gm21075; Gm	predicted gene 17167; predicted gene, 29964; predicted gene, 21075; predicted gene 13301	2.43
Ipw	imprinted gene in the Prader-Willi syndrome region	2.42
Gm24519	predicted gene, 24519 [Source:MGI Symbol;Acc:MGI:5454296]	2.39
Gm22304	predicted gene, 22304 [Source:MGI Symbol;Acc:MGI:5452081]	2.36
		2.36
		2.34
Dnaja3	DnaJ (Hsp40) homolog, subfamily A, member 3	2.29
Rps19-ps13	ribosomal protein S19, pseudogene 13	2.28

		2.23
Gm23054	predicted gene, 23054 [Source:MGI Symbol;Acc:MGI:5452831]	2.22
Cdc42ep1	CDC42 effector protein (Rho GTPase binding) 1	2.2
Pard6a	par-6 family cell polarity regulator alpha	2.2
Olfr26	olfactory receptor 26	2.2
Sfi1; Drg1	Sfi1 homolog, spindle assembly associated (yeast); developmentally regulated GTP binding protein 1	2.19
Gm24175	predicted gene, 24175	2.18
Cnih1	cornichon homolog 1 (Drosophila)	2.17
Mterf1a	mitochondrial transcription termination factor 1a	2.17
Olfr1461	olfactory receptor 1461	2.15
Olfr1384	olfactory receptor 1384	2.14
Gm20939	predicted gene, 20939	2.05
Olfr1265	olfactory receptor 1265	2.03
Mir449b	microRNA 449b	2.02
2500002B13Rik	RIKEN cDNA 2500002B13 gene	2.01
LOC105243270	40S ribosomal protein S2-like	-2.01
Necap1	NECAP endocytosis associated 1	-2.05
Gm5601; Ppp1cc	predicted pseudogene 5601; protein phosphatase 1, catalytic subunit, gamma isoform	-2.1
Gm9762	predicted pseudogene 9762 [Source:MGI Symbol;Acc:MGI:3704220]	-2.12
Tmem222	transmembrane protein 222	-2.12
Dnajc19-ps	DnaJ (Hsp40) homolog, subfamily C, member 19, pseudogene	-2.17
Tmem200b	transmembrane protein 200B	-2.19
Psmb1	proteasome (prosome, macropain) subunit, beta type 1	-2.21
Shoc2	soc-2 (suppressor of clear) homolog (C. elegans)	-2.21
Bax	BCL2-associated X protein	-2.26
Mir485; Mirg	microRNA 485; miRNA containing gene	-2.28
Arl6ip1	ADP-ribosylation factor-like 6 interacting protein 1	-2.28
Sft2d1	SFT2 domain containing 1	-2.29
Rpl4	ribosomal protein L4	-2.3
		-2.33
Gm15466	predicted gene 15466 [Source:MGI Symbol;Acc:MGI:3705491]	-2.34

Ddost	dolichyl-di-phosphooligosaccharide-protein glycotransferase	-2.36
Uso1	USO1 vesicle docking factor	-2.37
Cacng8	calcium channel, voltage-dependent, gamma subunit 8	-2.38
Mir1898	microRNA 1898	-2.39
Wbp5	WW domain binding protein 5	-2.39
Add1	adducin 1 (alpha)	-2.4
Slc25a44	solute carrier family 25, member 44	-2.44
Gm25759	predicted gene, 25759 [Source:MGI Symbol;Acc:MGI:5455536]	-2.45
Gm10053	predicted gene 10053 [Source:MGI Symbol;Acc:MGI:3704493]	-2.49
Cycs	cytochrome c, somatic	-2.49
Eef1b2	eukaryotic translation elongation factor 1 beta 2	-2.54
Ppp3r1; Wdr92	protein phosphatase 3, regulatory subunit B, alpha isoform (calcineurin B, type I); WD repeat domain 92	-2.54
Snapc3	small nuclear RNA activating complex, polypeptide 3	-2.54
Clec2l	C-type lectin domain family 2, member L	-2.55
Kcnc4	potassium voltage gated channel, Shaw-related subfamily, member 4	-2.56
2310057M21Rik	RIKEN cDNA 2310057M21 gene	-2.57
Zfr	zinc finger RNA binding protein	-2.58
Syt4	synaptotagmin IV	-2.59
Sv2b	synaptic vesicle glycoprotein 2 b	-2.61
n-R5s8	nuclear encoded rRNA 5S 8 [Source:MGI Symbol;Acc:MGI:4421742]	-2.63
Prrt2; Pagr1a	proline-rich transmembrane protein 2; PAXIP1 associated glutamate rich protein 1A	-2.67
Aftph	aftiphilin	-2.68
Nkain2	Na+/K+ transporting ATPase interacting 2	-2.71
Scn2a1	sodium channel, voltage-gated, type II, alpha 1	-2.71
Ddx26b	DEAD/H (Asp-Glu-Ala-Asp/His) box polypeptide 26B	-2.72
Rtcb	RNA 2,3-cyclic phosphate and 5-OH ligase	-2.75
Gdpd1	glycerophosphodiester phosphodiesterase domain containing 1	-2.81
Katna1	katanin p60 (ATPase-containing) subunit A1	-2.84
Exosc7	exosome component 7	-2.87
		-2.87

		-2.87
Cbr1	carbonyl reductase 1	-2.88
Arl8b	ADP-ribosylation factor-like 8B	-2.95
		-2.97
Rexo2	REX2, RNA exonuclease 2 homolog (S. cerevisiae)	-2.98
Gnai1	guanine nucleotide binding protein (G protein), alpha inhibiting 1	-3
Clta	clathrin, light polypeptide (Lca)	-3.01
Gm22205	predicted gene, 22205 [Source:MGI Symbol;Acc:MGI:5451982]	-3.04
Lin7b	lin-7 homolog B (C. elegans)	-3.06
Amd1	S-adenosylmethionine decarboxylase 1	-3.07
Cdc123	cell division cycle 123	-3.08
		-3.09
n-R5s80	nuclear encoded rRNA 5S 80 [Source:MGI Symbol;Acc:MGI:4421928]	-3.1
Gm6293	predicted pseudogene 6293 [Source:MGI Symbol;Acc:MGI:3649011]	-3.11
4933431E20Rik	RIKEN cDNA 4933431E20 gene	-3.12
Slc35g2	solute carrier family 35, member G2	-3.15
Usp9x	ubiquitin specific peptidase 9, X chromosome	-3.15
Pip4k2a	phosphatidylinositol-5-phosphate 4-kinase, type II, alpha	-3.18
Slc17a5	solute carrier family 17 (anion/sugar transporter), member 5	-3.18
Lrfn5	leucine rich repeat and fibronectin type III domain containing 5	-3.2
Tnks	tankyrase, TRF1-interacting ankyrin-related ADP-ribose polymerase	-3.22
Grpel1	GrpE-like 1, mitochondrial	-3.26
Napb	N-ethylmaleimide sensitive fusion protein attachment protein beta	-3.32
Sub1	SUB1 homolog (S. cerevisiae)	-3.43
Syt14	synaptotagmin XIV	-3.48
Edem2	ER degradation enhancer, mannosidase alpha-like 2	-3.55
Slc25a28	solute carrier family 25, member 28	-3.61
Churc1; Fntb	churchill domain containing 1; farnesyltransferase, CAAX box, beta	-3.7
Ccdc159	coiled-coil domain containing 159	-3.7
Rars	arginyl-tRNA synthetase	-3.82
Rabac1	Rab acceptor 1 (prenylated)	-3.84
		-3.85

Snhg14	small nucleolar RNA host gene 14	-3.88
Ttc3	tetratricopeptide repeat domain 3	-3.9
Ndufa4	NADH dehydrogenase (ubiquinone) 1 alpha subcomplex, 4	-3.93
Srsf9	serine/arginine-rich splicing factor 9	-3.98
Tctex1d2	Tctex1 domain containing 2	-4.02
Gm13770	predicted gene 13770 [Source:MGI Symbol;Acc:MGI:3651719]	-4.03
Nop10	NOP10 ribonucleoprotein	-4.04
Gm25817; Gm24766	predicted gene, 25817 [Source:MGI Symbol;Acc:MGI:5455594]; predicted gene, 24766 [Source:MGI Symbol;Acc:MGI:5454543]	-4.04
Gm25817; Gm24766	predicted gene, 25817 [Source:MGI Symbol;Acc:MGI:5455594]; predicted gene, 24766 [Source:MGI Symbol;Acc:MGI:5454543]	-4.04
Smim11	small integral membrane protein 11	-4.09
Dph6	diphthamine biosynthesis 6	-4.12
		-4.12
Camkv	CaM kinase-like vesicle-associated	-4.14
Tmem243	transmembrane protein 243, mitochondrial	-4.17
Sh3gl3	SH3-domain GRB2-like 3	-4.33
Tmem259	transmembrane protein 259	-4.44
Jkamp	JNK1/MAPK8-associated membrane protein	-4.48
Prpf18	PRP18 pre-mRNA processing factor 18 homolog (yeast)	-4.63
Skp1a	S-phase kinase-associated protein 1A	-4.64
Ilf2	interleukin enhancer binding factor 2	-4.73
Gm16357	predicted gene 16357	-4.86
Syne1	spectrin repeat containing, nuclear envelope 1	-4.87
Slc17a7	solute carrier family 17 (sodium-dependent inorganic phosphate cotransporter), member 7	-4.87
Zc4h2	zinc finger, C4H2 domain containing	-5.02
Rnf7	ring finger protein 7	-5.22
Eif3j2; Eif3j1	eukaryotic translation initiation factor 3, subunit J2; eukaryotic translation initiation factor 3, subunit J1	-5.33
Arxes2	adipocyte-related X-chromosome expressed sequence 2	-5.51
		-5.59
S100a6	S100 calcium binding protein A6 (calcyclin)	-5.59
Mir3075	microRNA 3075	-5.93
Scrg1	scrapie responsive gene 1	-5.98

Gm12396	predicted gene 12396 [Source:MGI Symbol;Acc:MGI:3649201]	-5.99
Cdc26	cell division cycle 26	-6.1
Ddx5	DEAD (Asp-Glu-Ala-Asp) box polypeptide 5	-6.14
Gm23546	predicted gene, 23546 [Source:MGI Symbol;Acc:MGI:5453323]	-6.14
AF357425	snoRNA AF357425	-6.5
Lrrtm3	leucine rich repeat transmembrane neuronal 3	-6.52
Hmga1-rs1	high mobility group AT-hook I, related sequence 1	-6.91
Ccdc25	coiled-coil domain containing 25	-7.08
Mir668	microRNA 668	-7.17
Syne1	spectrin repeat containing, nuclear envelope 1	-7.18
Gm25635	predicted gene, 25635 [Source:MGI Symbol;Acc:MGI:5455412]	-7.41
Fam160a1; Arfip1	family with sequence similarity 160, member A1; ADP-ribosylation factor interacting protein 1	-8.03
Wdr82	WD repeat domain containing 82	-8.18
Gm26202	predicted gene, 26202 [Source:MGI Symbol;Acc:MGI:5455979]	-9.27
		-9.44
Gm23301	predicted gene, 23301 [Source:MGI Symbol;Acc:MGI:5453078]	-9.45
Rbm7	RNA binding motif protein 7	-9.79
DQ267100; Rian	snoRNA DQ267100; RNA imprinted and accumulated in nucleus	-9.95
Mpc1; Mpc1-ps	mitochondrial pyruvate carrier 1; mitochondrial pyruvate carrier 1, pseudogene	-10.41
Gm22858	predicted gene, 22858 [Source:MGI Symbol;Acc:MGI:5452635]	-10.53
Gm25147	predicted gene, 25147 [Source:MGI Symbol;Acc:MGI:5454924]	-11.14
Syne1	spectrin repeat containing, nuclear envelope 1	-11.2
Gm21399	predicted gene, 21399 [Source:MGI Symbol;Acc:MGI:5434754]	-12.5
Gm26347	predicted gene, 26347 [Source:MGI Symbol;Acc:MGI:5456124]	-12.83
1700025G04Rik	RIKEN cDNA 1700025G04 gene	-13.51
Psenen	presenilin enhancer 2 homolog (C. elegans)	-14.67
Tmem196	transmembrane protein 196	-16
Snord65	small nucleolar RNA, C/D box 65	-18.49
Gm25873	predicted gene, 25873 [Source:MGI Symbol;Acc:MGI:5455650]	-19.02

Gm26265	predicted gene, 26265 [Source:MGI Symbol;Acc:MGI:5456042]	-19.24
Mir329	microRNA 329	-27.42
Mir376b	microRNA 376b	-29.43
Snord61	small nucleolar RNA, C/D box 61	-33.17
Atxn7l1	ataxin 7-like 1	-47.16

Table S4: Differentially expressed genes in control diet (CD) fed LDL-R -/- mice when compared to control diet (CD) fed WT mice.

Gene Symbol	Gene Description	Fold Change
LOC105245453; Gm2399	nidogen-1-like; predicted gene 2399 [Source:MGI Symbol;Acc:MGI:3780567]	129.21
Gm22882	predicted gene, 22882 [Source:MGI Symbol;Acc:MGI:5452659]	110.37
Mir300	microRNA 300	107.74
Scarna13; Mir3069	small Cajal body-specific RNA 1; microRNA 3069	89.76
Gm25559	predicted gene, 25559 [Source:MGI Symbol;Acc:MGI:5455336]	84.1
Gm23111	predicted gene, 23111 [Source:MGI Symbol;Acc:MGI:5452888]	73.6
Mir3097	microRNA 3097	68.77
Gm25224	predicted gene, 25224 [Source:MGI Symbol;Acc:MGI:5455001]	64.19
Chtf8	CTF8, chromosome transmission fidelity factor 8	62.9
Rpl3	ribosomal protein L3	59.66
mt-Tv	mitochondrially encoded tRNA valine [Source:MGI Symbol;Acc:MGI:102472]	58.1
Gm17146	predicted gene 17146 [Source:MGI Symbol;Acc:MGI:4937973]	56.05
Syne1	spectrin repeat containing, nuclear envelope 1	50.84
Gm24155; Gm9916	predicted gene, 24155 [Source:MGI Symbol;Acc:MGI:5453932]; predicted gene 9916 [Source:MGI Symbol;Acc:MGI:3642633]	47.21
Commd4	COMM domain containing 4	46.84
Gm25357	predicted gene, 25357 [Source:MGI Symbol;Acc:MGI:5455134]	45.7
Syne1	spectrin repeat containing, nuclear envelope 1	42.58
Trf	transferrin	39.73
Kansl2-ps	KAT8 regulatory NSL complex subunit 2, pseudogene	39.63
DQ267101	snoRNA DQ267101	37.28
Mir1912	microRNA 1912	37.22
Syne1	spectrin repeat containing, nuclear envelope 1	37.21
		36.61
Syne1	spectrin repeat containing, nuclear envelope 1	36.6
AF357428	snoRNA AF357428 [Source:MGI Symbol;Acc:MGI:3053436]	36.2
Fads3	fatty acid desaturase 3	35.29
Gm23232	predicted gene, 23232 [Source:MGI Symbol;Acc:MGI:5453009]	34.77

Syne1	spectrin repeat containing, nuclear envelope 1	33.49
		33.13
Mir218-1	microRNA 218-1	31.9
Mir15a	microRNA 15a	31.33
Shisa4	shisa family member 4	29.9
Rpsa	ribosomal protein SA	29.74
Gm25406	predicted gene, 25406 [Source:MGI Symbol;Acc:MGI:5455183]	29.68
Gm10139	predicted gene 10139	28.36
		28.17
Snora17; Snhg7	small nucleolar RNA, H/ACA box 17; small nucleolar RNA host gene 7	27.77
Gm24009	predicted gene, 24009 [Source:MGI Symbol;Acc:MGI:5453786]	27.12
Gm10800	predicted gene 10800 [Source:MGI Symbol;Acc:MGI:3641657]	26.45
LOC100862043	protein FAM205A-like	26.35
		25.99
Gm5741	predicted gene 5741	25.95
2610524H06Rik	RIKEN cDNA 2610524H06 gene	25.86
Mir1188	microRNA 1188	25.82
Ntng1	netrin G1	25.61
n-R5s98	nuclear encoded rRNA 5S 98 [Source:MGI Symbol;Acc:MGI:4421946]	25.39
Tmem130	transmembrane protein 130	24.5
Mir487b	microRNA 487b	24.48
Lgals1	lectin, galactose binding, soluble 1	24.45
Atraid	all-trans retinoic acid induced differentiation factor	24.32
Snora44	small nucleolar RNA, H/ACA box 44	24.25
Gm8069	predicted pseudogene 8069	24.23
Gm25732	predicted gene, 25732 [Source:MGI Symbol;Acc:MGI:5455509]	24.04
Eif4a1	eukaryotic translation initiation factor 4A1	24.03
		23.94
Gm9396	predicted gene 9396 [Source:MGI Symbol;Acc:MGI:3645563]	23
Znhit2	zinc finger, HIT domain containing 2	22.87
Snord17	small nucleolar RNA, C/D box 17	22.87
		22.65
Mir5125; Srrm2	microRNA 5125; serine/arginine repetitive matrix 2	22.47
Sbds	Shwachman-Bodian-Diamond syndrome homolog (human)	22.19
		22.18

Kctd13	potassium channel tetramerisation domain containing 13	21.87
Syne1	spectrin repeat containing, nuclear envelope 1	21.76
		21.39
		21.39
Gm25376	predicted gene, 25376 [Source:MGI Symbol;Acc:MGI:5455153]	21.03
Snhg11	small nucleolar RNA host gene 11	20.82
Hspa12a	heat shock protein 12A	20.75
Gm23508; Rian	predicted gene, 23508 [Source:MGI Symbol;Acc:MGI:5453285]; RNA imprinted and accumulated in nucleus [Source:MGI Symbol;Acc:MGI:1922995]	20.16
Polr2d	polymerase (RNA) II (DNA directed) polypeptide D	19.57
Rarres2	retinoic acid receptor responder (tazarotene induced) 2	19.1
Scarna3b; Mir1843a	small Cajal body-specific RNA 3B; microRNA 1843a	19.08
		18.95
Gm12115	predicted gene 12115 [Source:MGI Symbol;Acc:MGI:3652288]	18.79
Pfdn1	prefoldin 1	18.78
Syne1	spectrin repeat containing, nuclear envelope 1	18.64
Actr3	ARP3 actin-related protein 3	18.4
Gm5908	predicted gene 5908	18.09
Trp53rka	transformation related protein 53 regulating kinase A	18
Gm26225	predicted gene, 26225 [Source:MGI Symbol;Acc:MGI:5456002]	17.92
Mir694	microRNA 694	17.9
Commd6	COMM domain containing 6	17.65
2010111I01Rik; Mir23b	RIKEN cDNA 2010111I01 gene; microRNA 23b	17.5
Zfp275	zinc finger protein 275	17.18
		16.92
H2-T22; H2-T9	histocompatibility 2, T region locus 22; histocompatibility 2, T region locus 9	16.72
Gm6736	predicted gene 6736 [Source:MGI Symbol;Acc:MGI:3643048]	16.65
Mrpl53	mitochondrial ribosomal protein L53	16.41
Tstd1	thiosulfate sulfurtransferase (rhodanese)-like domain containing 1	16.39
Naa60	N(alpha)-acetyltransferase 60, NatF catalytic subunit	15.87
Tdp1	tyrosyl-DNA phosphodiesterase 1	15.85

Anapc16	anaphase promoting complex subunit 16	15.73
Snord104	small nucleolar RNA, C/D box 104	15.69
Sar1b	SAR1 gene homolog B (S. cerevisiae)	15.52
Jmjd8	jumonji domain containing 8	15.35
		15.3
		15.08
Asna1	arsA arsenite transporter, ATP-binding, homolog 1 (bacterial)	14.92
Syne1	spectrin repeat containing, nuclear envelope 1	14.59
Psip1	PC4 and SFRS1 interacting protein 1	14.49
Cd276	CD276 antigen	14.48
Deb1	differentially expressed in B16F10 1	14.4
Fads2	fatty acid desaturase 2	14.37
Anp32b	acidic (leucine-rich) nuclear phosphoprotein 32 family, member B	14.33
Rqcd1	rcd1 (required for cell differentiation) homolog 1 (S. pombe)	14.28
Ap3m2	adaptor-related protein complex 3, mu 2 subunit	14.23
Snora30	small nucleolar RNA, H/ACA box 30	14.11
Snord34; Rpl13a	small nucleolar RNA, C/D box 34; ribosomal protein L13A	14.08
Spag7	sperm associated antigen 7	13.94
Rtfdc1	replication termination factor 2 domain containing 1	13.83
Gm10718	predicted gene 10718 [Source:MGI Symbol;Acc:MGI:3642028]	13.65
Gm22328	predicted gene, 22328 [Source:MGI Symbol;Acc:MGI:5452105]	13.59
Snn	stannin	13.52
Lman2	lectin, mannose-binding 2	13.47
Syne1	spectrin repeat containing, nuclear envelope 1	13.43
Syne1	spectrin repeat containing, nuclear envelope 1	13.28
Fam103a1	family with sequence similarity 103, member A1	13.26
Zfp13	zinc finger protein 13	13.23
Lrpap1	low density lipoprotein receptor-related protein associated protein 1	13
E530001F21Rik	RIKEN cDNA E530001F21 gene	12.97
Msl3	male-specific lethal 3 homolog (Drosophila)	12.93
Gm24411; Snhg17	predicted gene, 24411 [Source:MGI Symbol;Acc:MGI:5454188]; small nucleolar RNA host gene 17 [Source:MGI Symbol;Acc:MGI:1915358]	12.88
Ddx19a	DEAD (Asp-Glu-Ala-Asp) box polypeptide 19a	12.65
Arhgdia	Rho GDP dissociation inhibitor (GDI) alpha	12.62

Tmub2	transmembrane and ubiquitin-like domain containing 2	12.6
Gm2011; 3110057O12Rik	predicted gene 2011; RIKEN cDNA 3110057O12 gene	12.49
AW495222	expressed sequence AW495222	12.48
Abhd8	abhydrolase domain containing 8	12.4
Gm6158	predicted gene 6158	12.24
H2-Q5	histocompatibility 2, Q region locus 5	12.21
Ppp4r2	protein phosphatase 4, regulatory subunit 2	12.12
Syne1	spectrin repeat containing, nuclear envelope 1	12.09
Sfxn1	sideroflexin 1	12.01
Ercc5	excision repair cross-complementing rodent repair deficiency, complementation group 5	11.91
Cep83os	centrosomal protein 83, opposite strand	11.85
Shank3	SH3/ankyrin domain gene 3	11.84
Ccdc32	coiled-coil domain containing 32	11.74
Arl3	ADP-ribosylation factor-like 3	11.73
Smim12	small integral membrane protein 12	11.7
Dync1i2	dynein cytoplasmic 1 intermediate chain 2	11.66
Bcl9	B cell CLL/lymphoma 9	11.65
Vps37c	vacuolar protein sorting 37C (yeast)	11.39
Apc	adenomatosis polyposis coli	11.38
Gm25053	predicted gene, 25053 [Source:MGI Symbol;Acc:MGI:5454830]	11.33
Vps41	vacuolar protein sorting 41 (yeast)	11.19
Gm6642	ubiquitin-like 5 pseudogene	11.19
Gm24063	predicted gene, 24063 [Source:MGI Symbol;Acc:MGI:5453840]	11.16
Syne1	spectrin repeat containing, nuclear envelope 1	11.08
Gm11360	predicted gene 11360 [Source:MGI Symbol;Acc:MGI:3649916]	11.06
Gm4943	predicted pseudogene 4943	11
Nacc1	nucleus accumbens associated 1, BEN and BTB (POZ) domain containing	10.95
Rasl10b	RAS-like, family 10, member B	10.93
Ptpn5	protein tyrosine phosphatase, non-receptor type 5	10.79
Rit1	Ras-like without CAAX 1	10.68
Gpbp1	GC-rich promoter binding protein 1	10.65
Mllt1	myeloid/lymphoid or mixed-lineage leukemia (trithorax homolog, Drosophila); translocated to, 1	10.6
Gm22716	predicted gene, 22716 [Source:MGI Symbol;Acc:MGI:5452493]	10.6
Zfp1	zinc finger protein 1	10.56
Mkrn2	makorin, ring finger protein, 2	10.5

Nrbp2	nuclear receptor binding protein 2	10.46
Gm24556	predicted gene, 24556 [Source:MGI Symbol;Acc:MGI:5454333]	10.43
Dcaf8	DDB1 and CUL4 associated factor 8	10.41
LOC102640399; Gm22131	uncharacterized LOC102640399; predicted gene, 22131 [Source:MGI Symbol;Acc:MGI:5451908]	10.39
Pfn4	profilin family, member 4	10.36
Gm26398	predicted gene, 26398 [Source:MGI Symbol;Acc:MGI:5456175]	10.29
Gm25973	predicted gene, 25973 [Source:MGI Symbol;Acc:MGI:5455750]	10.16
Mapk3	mitogen-activated protein kinase 3	10.1
Nol8	nucleolar protein 8	10.07
Gm23039	predicted gene, 23039 [Source:MGI Symbol;Acc:MGI:5452816]	10.03
Gp1bb; Sept5	glycoprotein Ib, beta polypeptide; septin 5	10.02
F3	coagulation factor III	9.99
Gm12396	predicted gene 12396 [Source:MGI Symbol;Acc:MGI:3649201]	9.99
Lxn	latexin	9.91
Gm6644	Akr1b3 pseudogene	9.89
		9.88
Gkap1	G kinase anchoring protein 1	9.82
Vmn2r38	vomeronasal 2, receptor 38	9.79
Fam96a	family with sequence similarity 96, member A	9.75
5031439G07Rik	RIKEN cDNA 5031439G07 gene	9.7
Gm14295; Gm38519	predicted gene 14295; predicted gene, 38519	9.46
Peg10	paternally expressed 10	9.45
Cxxc5	CXXC finger 5	9.44
Fam131a	family with sequence similarity 131, member A	9.38
Gm14817	predicted gene 14817 [Source:MGI Symbol;Acc:MGI:3705159]	9.38
		9.35
		9.32
Rnf41	ring finger protein 41	9.3
Gm5830	predicted pseudogene 5830 [Source:MGI Symbol;Acc:MGI:3647375]	9.28
Rtn2	reticulon 2 (Z-band associated protein)	9.28
Ptch1	patched homolog 1	9.25
Gja1	gap junction protein, alpha 1	9.18
		9.18
Rps19bp1	ribosomal protein S19 binding protein 1	9.13
Gsk3a	glycogen synthase kinase 3 alpha	9.1
Actr1b	ARP1 actin-related protein 1B, centractin beta	9.09

Aph1a	anterior pharynx defective 1a homolog (C. elegans)	9.08
Nefm	neurofilament, medium polypeptide	9.07
Abcf2	ATP-binding cassette, sub-family F (GCN20), member 2	9.04
Atp6v0c-ps2; Atp6v0c	ATPase, H+ transporting, lysosomal V0 subunit C, pseudogene 2; ATPase, H+ transporting, lysosomal V0 subunit C	8.99
Eps15	epidermal growth factor receptor pathway substrate 15	8.92
Nfyc	nuclear transcription factor-Y gamma	8.91
Gm8730	predicted pseudogene 8730 [Source:MGI Symbol;Acc:MGI:3644565]	8.88
Cops5	COP9 (constitutive photomorphogenic) homolog, subunit 5 (Arabidopsis thaliana)	8.84
mt-Tt	mitochondrially encoded tRNA threonine [Source:MGI Symbol;Acc:MGI:102473]	8.8
Otud3	OTU domain containing 3	8.79
Zfp938	zinc finger protein 938	8.72
Vps36	vacuolar protein sorting 36 (yeast)	8.72
2310036O22Rik	RIKEN cDNA 2310036O22 gene	8.72
Luzp1	leucine zipper protein 1	8.71
		8.69
Calr	calreticulin	8.69
Gdap1l1	ganglioside-induced differentiation-associated protein 1-like 1	8.6
Kctd13	potassium channel tetramerisation domain containing 13	8.53
Pip4k2b	phosphatidylinositol-5-phosphate 4-kinase, type II, beta	8.5
Mocs2	molybdenum cofactor synthesis 2	8.5
Snrpa	small nuclear ribonucleoprotein polypeptide A	8.47
1110012L19Rik	RIKEN cDNA 1110012L19 gene	8.47
Ctns	cystinosis, nephropathic	8.46
Ermn	ermin, ERM-like protein	8.45
Tubg1	tubulin, gamma 1	8.44
Srrm4	serine/arginine repetitive matrix 4	8.44
Uba3	ubiquitin-like modifier activating enzyme 3	8.44
Ifitm3	interferon induced transmembrane protein 3	8.43
Gm22707	predicted gene, 22707 [Source:MGI Symbol;Acc:MGI:5452484]	8.42
Gm9954	predicted gene 9954	8.35
Cox20	COX20 Cox2 chaperone	8.32
Scn1b	sodium channel, voltage-gated, type I, beta	8.28

Gm26809	predicted gene, 26809 [Source:MGI Symbol;Acc:MGI:5477303]	8.26
Tinagl1	tubulointerstitial nephritis antigen-like 1	8.18
Syne1	spectrin repeat containing, nuclear envelope 1	8.09
Snord55	small nucleolar RNA, C/D box 55	8
		7.94
Fars2	phenylalanine-tRNA synthetase 2 (mitochondrial)	7.94
Emg1	EMG1 nucleolar protein homolog (S. cerevisiae)	7.94
Gm25788	predicted gene, 25788 [Source:MGI Symbol;Acc:MGI:5455565]	7.89
Snx3	sorting nexin 3	7.86
Eif4h	eukaryotic translation initiation factor 4H	7.86
Gm5454	predicted gene 5454	7.84
1700029P11Rik	RIKEN cDNA 1700029P11 gene	7.82
Golga4	golgi autoantigen, golgin subfamily a, 4	7.8
		7.79
Gm25396	predicted gene, 25396 [Source:MGI Symbol;Acc:MGI:5455173]	7.76
		7.67
		7.63
Spr	sepiapterin reductase	7.63
Gm25615	predicted gene, 25615 [Source:MGI Symbol;Acc:MGI:5455392]	7.55
Mt3	metallothionein 3	7.52
Lypla2	lysophospholipase 2	7.5
Anapc15-ps	anaphase prompoting complex C subunit 15, pseudogene [Source:MGI Symbol;Acc:MGI:3646302]	7.47
Plac9b; Plac9a; Gm9780	placenta specific 9b; placenta specific 9a; predicted gene 9780	7.47
Cdkl1	cyclin-dependent kinase-like 1 (CDC2-related kinase)	7.44
Psmg1	proteasome (prosome, macropain) assembly chaperone 1	7.43
Plppr4	phospholipid phosphatase related 4	7.41
Ddost	dolichyl-di-phosphooligosaccharide-protein glycotransferase	7.38
Mir466f-4	microRNA 466f-4	7.37
Mrps16	mitochondrial ribosomal protein S16	7.37
Adnp; Dpm1	activity-dependent neuroprotective protein; dolichol-phosphate (beta-D) mannosyltransferase 1	7.28
Slc25a16	solute carrier family 25 (mitochondrial carrier, Graves disease autoantigen), member 16	7.21
Rps15a-ps5	ribosomal protein S15A, pseudogene 5	7.21

Ephb1	Eph receptor B1	7.2
Fam168a	family with sequence similarity 168, member A	7.15
Gm8054	predicted pseudogene 8054 [Source:MGI Symbol;Acc:MGI:3643693]	7.1
Med11	mediator complex subunit 11	7.08
Trim8	tripartite motif-containing 8	7.08
Arid4a	AT rich interactive domain 4A (RBP1-like)	7.07
		7.05
Mir5099	microRNA 5099	7.04
Gm10291	predicted pseudogene 10291 [Source:MGI Symbol;Acc:MGI:3641638]	7.03
Gm25683	predicted gene, 25683 [Source:MGI Symbol;Acc:MGI:5455460]	7.02
		7
Cpne9	copine family member IX	6.98
Chd3os	chromodomain helicase DNA binding protein 3, opposite strand	6.97
Sirt2	sirtuin 2	6.97
D2Wsu81e	DNA segment, Chr 2, Wayne State University 81, expressed	6.94
Hist1h2af	histone cluster 1, H2af	6.89
Dap	death-associated protein	6.88
Igkv1-135	immunoglobulin kappa variable 1-135	6.88
Scarna2	small Cajal body-specific RNA 2	6.87
		6.84
Raver2	ribonucleoprotein, PTB-binding 2	6.82
Sh3glb1	SH3-domain GRB2-like B1 (endophilin)	6.81
Sema6b	sema domain, transmembrane domain (TM), and cytoplasmic domain, (semaphorin) 6B	6.79
Hmgn3	high mobility group nucleosomal binding domain 3	6.77
Rpl17	ribosomal protein L17	6.73
Acadsb	acyl-Coenzyme A dehydrogenase, short/branched chain	6.72
Mir337	microRNA 337	6.7
Letmd1	LETM1 domain containing 1	6.7
		6.68
Bex1	brain expressed gene 1	6.61
G3bp2	GTPase activating protein (SH3 domain) binding protein 2	6.57
Guk1	guanylate kinase 1	6.56
Pdk4	pyruvate dehydrogenase kinase, isoenzyme 4	6.55
Gpr151	G protein-coupled receptor 151	6.54
Prkcq	protein kinase C, theta	6.54

Sema6d	sema domain, transmembrane domain (TM), and cytoplasmic domain, (semaphorin) 6D	6.54
Taf11	TAF11 RNA polymerase II, TATA box binding protein (TBP)-associated factor	6.51
Gm25093	predicted gene, 25093 [Source:MGI Symbol;Acc:MGI:5454870]	6.5
Trdv2-2	T cell receptor delta variable 2-2	6.48
		6.46
Gm23456	predicted gene, 23456 [Source:MGI Symbol;Acc:MGI:5453233]	6.44
Pycrl	pyrroline-5-carboxylate reductase-like	6.43
Sec13	SEC13 homolog, nuclear pore and COPII coat complex component	6.42
Tacc1	transforming, acidic coiled-coil containing protein 1	6.41
Gm11549	predicted gene 11549	6.4
		6.38
Txnl4b	thioredoxin-like 4B	6.37
Gm10154	predicted gene 10154 [Source:MGI Symbol;Acc:MGI:3642271]	6.36
Usp29	ubiquitin specific peptidase 29	6.36
Mir3058	microRNA 3058	6.33
Syngap1	synaptic Ras GTPase activating protein 1 homolog (rat)	6.29
Cdkn2aipnl	CDKN2A interacting protein N-terminal like	6.24
Hdac5	histone deacetylase 5	6.24
Gm25128	predicted gene, 25128 [Source:MGI Symbol;Acc:MGI:5454905]	6.23
Atg13	autophagy related 13	6.22
Taf6l	TAF6-like RNA polymerase II, p300/CBP-associated factor (PCAF)-associated factor	6.21
		6.18
Map4k2	mitogen-activated protein kinase kinase kinase kinase 2	6.16
Gm5921	predicted gene 5921 [Source:MGI Symbol;Acc:MGI:3648968]	6.15
H2-Ke6	H2-K region expressed gene 6	6.13
		6.12
		6.12
		6.12
Atp1b3	ATPase, Na+/K+ transporting, beta 3 polypeptide	6.09
		6.08
Gm5637	predicted pseudogene 5637 [Source:MGI Symbol;Acc:MGI:3648120]	6.08
Mob3b	MOB kinase activator 3B	6.02

C1qb	complement component 1, q subcomponent, beta polypeptide	6.02
Nab1	Ngfi-A binding protein 1	5.99
Jrkl	Jrk-like	5.97
Pnpla6	patatin-like phospholipase domain containing 6	5.96
Mir181c	microRNA 181c	5.96
Gm9234	predicted pseudogene 9234	5.91
Tbc1d30	TBC1 domain family, member 30	5.9
Lef1	lymphoid enhancer binding factor 1	5.89
		5.89
Phlda3	pleckstrin homology-like domain, family A, member 3	5.87
Parp2	poly (ADP-ribose) polymerase family, member 2	5.85
Mid2	midline 2	5.85
Tmed9	transmembrane emp24 protein transport domain containing 9	5.83
Mbnl1	muscleblind-like 1 (Drosophila)	5.82
Mgst3	microsomal glutathione S-transferase 3	5.81
		5.79
Stx5a	syntaxin 5A	5.78
Gm13031	predicted gene 13031	5.76
Txndc12	thioredoxin domain containing 12 (endoplasmic reticulum)	5.74
Scn2b	sodium channel, voltage-gated, type II, beta	5.74
Snrnp35	small nuclear ribonucleoprotein 35 (U11/U12)	5.73
		5.71
Egln1	egl-9 family hypoxia-inducible factor 1	5.69
		5.69
Nr1d2	nuclear receptor subfamily 1, group D, member 2	5.68
Cmtm3	CKLF-like MARVEL transmembrane domain containing 3	5.63
3110039I08Rik	RIKEN cDNA 3110039I08 gene	5.63
Zfp68	zinc finger protein 68	5.61
Plekha2	pleckstrin homology domain-containing, family A (phosphoinositide binding specific) member 2	5.56
Ttc1	tetratricopeptide repeat domain 1	5.54
Crip1	cysteine-rich protein 1 (intestinal)	5.51
Zmat2	zinc finger, matrin type 2	5.51
4930567H12Rik	RIKEN cDNA 4930567H12 gene	5.51
Gm26148	predicted gene, 26148 [Source:MGI Symbol;Acc:MGI:5455925]	5.5
Ptk2	PTK2 protein tyrosine kinase 2	5.48
Nhp2l1	NHP2 non-histone chromosome protein 2-like 1 (S. cerevisiae)	5.46

Ttc14	tetratricopeptide repeat domain 14	5.46
Gm23458	predicted gene, 23458 [Source:MGI Symbol;Acc:MGI:5453235]	5.46
Snord8	small nucleolar RNA, C/D box 8	5.44
		5.44
Ilk	integrin linked kinase	5.44
Cadm4	cell adhesion molecule 4	5.41
Brcc3	BRCA1/BRCA2-containing complex, subunit 3	5.41
3110052M02Rik	RIKEN cDNA 3110052M02 gene	5.4
Dr1	down-regulator of transcription 1	5.38
Gm10293	predicted pseudogene 10293 [Source:MGI Symbol;Acc:MGI:3704216]	5.36
Gm23745	predicted gene, 23745 [Source:MGI Symbol;Acc:MGI:5453522]	5.36
Klhl18	kelch-like 18	5.34
Bst2	bone marrow stromal cell antigen 2	5.32
Gm22962	predicted gene, 22962 [Source:MGI Symbol;Acc:MGI:5452739]	5.32
Reck	reversion-inducing-cysteine-rich protein with kazal motifs	5.31
Mrps11	mitochondrial ribosomal protein S11	5.29
Vmn2r33	vomeronasal 2, receptor 33	5.29
Ankrd34a	ankyrin repeat domain 34A	5.28
Atxn7l1	ataxin 7-like 1	5.27
Tdg	thymine DNA glycosylase	5.26
		5.26
Rpn1	ribophorin I	5.24
Gtf2b	general transcription factor IIB	5.23
Ndufb2	NADH dehydrogenase (ubiquinone) 1 beta subcomplex, 2	5.21
Arrb2	arrestin, beta 2	5.2
Vat1	vesicle amine transport protein 1 homolog (T californica)	5.16
Fdft1	farnesyl diphosphate farnesyl transferase 1	5.16
Chrnb2	cholinergic receptor, nicotinic, beta polypeptide 2 (neuronal)	5.13
Syne1	spectrin repeat containing, nuclear envelope 1	5.12
Srp19	signal recognition particle 19	5.11
Cd300lh	CD300 antigen like family member H	5.1
Lamtor4	late endosomal/lysosomal adaptor, MAPK and MTOR activator 4	5.1
Gabra4	gamma-aminobutyric acid (GABA) A receptor, subunit alpha 4	5.1
Tmem91	transmembrane protein 91	5.1

Zswim8	zinc finger SWIM-type containing 8	5.09
Samd10	sterile alpha motif domain containing 10	5.09
Nfia	nuclear factor I/A	5.08
Ost4	oligosaccharyltransferase 4 homolog (S. cerevisiae)	5.06
Sdc4	syndecan 4	5.05
Btg2	B cell translocation gene 2, anti-proliferative	5.04
Mpv17l2	MPV17 mitochondrial membrane protein-like 2	5.04
Rcan3; Mir700	regulator of calcineurin 3; microRNA 700	5.02
Bcam	basal cell adhesion molecule	5.02
Strn	striatin, calmodulin binding protein	5.01
Gm15292	predicted gene 15292	5.01
Syne1	spectrin repeat containing, nuclear envelope 1	5
Gm22039	predicted gene, 22039 [Source:MGI Symbol;Acc:MGI:5451816]	5
Tmem150a	transmembrane protein 150A	4.99
Mrpl16	mitochondrial ribosomal protein L16	4.98
Gm25518	predicted gene, 25518 [Source:MGI Symbol;Acc:MGI:5455295]	4.97
Cdh6	cadherin 6	4.95
		4.95
		4.95
Cables2	CDK5 and Abl enzyme substrate 2	4.93
Taf1a	TATA box binding protein (Tbp)-associated factor, RNA polymerase I, A	4.91
Elp2	elongator acetyltransferase complex subunit 2	4.91
Chchd2	coiled-coil-helix-coiled-coil-helix domain containing 2	4.91
Gm13189	predicted gene 13189 [Source:MGI Symbol;Acc:MGI:3650860]	4.9
Rap1gap2	RAP1 GTPase activating protein 2	4.89
Cp	ceruloplasmin	4.89
Plac9b; Plac9a; Gm9780	placenta specific 9b; placenta specific 9a; predicted gene 9780	4.88
Plac9b; Plac9a; Gm9780	placenta specific 9b; placenta specific 9a; predicted gene 9780	4.88
Gm25321	predicted gene, 25321 [Source:MGI Symbol;Acc:MGI:5455098]	4.88
AW554918	expressed sequence AW554918	4.87
Gm4604	predicted gene 4604	4.87
mt-Tc	mitochondrially encoded tRNA cysteine [Source:MGI Symbol;Acc:MGI:102490]	4.87
		4.87
Rpa3	replication protein A3	4.86
Lonp2	lon peptidase 2, peroxisomal	4.86

Zbtb38; E030011O05Rik	zinc finger and BTB domain containing 38; RIKEN cDNA E030011O05 gene	4.85
Dusp7	dual specificity phosphatase 7	4.84
Lrch1	leucine-rich repeats and calponin homology (CH) domain containing 1	4.83
Gm12082; Gm40818	predicted gene 12082; predicted gene, 40818	4.82
Axin1	axin 1	4.82
		4.82
Sf3b5	splicing factor 3b, subunit 5	4.81
Toporsos	topoisomerase I binding, arginine/serine-rich, opposite strand	4.81
Rps7	ribosomal protein S7	4.8
Nexn	nexilin	4.79
Irf8	interferon regulatory factor 8	4.79
Cct4	chaperonin containing Tcp1, subunit 4 (delta)	4.76
Cldn5	claudin 5	4.76
Mettl5	methyltransferase like 5	4.76
Bin1	bridging integrator 1	4.75
Ostc	oligosaccharyltransferase complex subunit	4.75
Ppp1r37	protein phosphatase 1, regulatory subunit 37	4.75
Ralgds	ral guanine nucleotide dissociation stimulator	4.74
		4.73
Fbxw7	F-box and WD-40 domain protein 7	4.73
Rpl34-ps1	ribosomal protein L34, pseudogene 1	4.73
Zmym6	zinc finger, MYM-type 6	4.72
Gm3878	predicted gene 3878	4.71
Ndst3	N-deacetylase/N-sulfotransferase (heparan glucosaminyl) 3	4.71
Cdh20	cadherin 20	4.7
Morn4	MORN repeat containing 4	4.69
Fbxo25	F-box protein 25	4.69
Eno3	enolase 3, beta muscle	4.68
Ncaph2	non-SMC condensin II complex, subunit H2	4.68
		4.67
Syne1	spectrin repeat containing, nuclear envelope 1	4.66
Gm6713	predicted gene 6713	4.66
Gm24154	predicted gene, 24154 [Source:MGI Symbol;Acc:MGI:5453931]	4.65
Tmbim6	transmembrane BAX inhibitor motif containing 6	4.64
Gm5428	predicted gene 5428 [Source:MGI Symbol;Acc:MGI:3647789]	4.63
Hes5	hairy and enhancer of split 5 (Drosophila)	4.63
Zfp128	zinc finger protein 128	4.62

Nudt4	nudix (nucleoside diphosphate linked moiety X)-type motif 4	4.6
U2af1	U2 small nuclear ribonucleoprotein auxiliary factor (U2AF) 1	4.6
		4.6
Larp4b	La ribonucleoprotein domain family, member 4B	4.59
Gm12905	predicted gene 12905 [Source:MGI Symbol;Acc:MGI:3702581]	4.59
Rab21	RAB21, member RAS oncogene family	4.58
Tcrg-V3	T cell receptor gamma, variable 3	4.58
Tubb2b	tubulin, beta 2B class IIB	4.57
Nsa2	NSA2 ribosome biogenesis homolog (S. cerevisiae)	4.56
Herpud2	HERPUD family member 2	4.55
Tox4	TOX high mobility group box family member 4	4.53
Wbp4	WW domain binding protein 4	4.53
Syne1	spectrin repeat containing, nuclear envelope 1	4.52
Kdelr1	KDEL (Lys-Asp-Glu-Leu) endoplasmic reticulum protein retention receptor 1	4.52
Zcchc18	zinc finger, CCHC domain containing 18	4.52
Ap4m1	adaptor-related protein complex AP-4, mu 1	4.51
Mrpl19	mitochondrial ribosomal protein L19	4.5
Mtss1l	metastasis suppressor 1-like	4.5
		4.49
Syt1	synaptotagmin I	4.47
Gm24525	predicted gene, 24525 [Source:MGI Symbol;Acc:MGI:5454302]	4.47
A230057D06Rik	RIKEN cDNA A230057D06 gene	4.47
Nt5m	5,3-nucleotidase, mitochondrial	4.45
Gm13441	predicted gene 13441 [Source:MGI Symbol;Acc:MGI:3649406]	4.44
Ptms	parathymosin	4.43
		4.43
Prdx6	peroxiredoxin 6	4.42
Gm21540; Tmed2	predicted gene, 21540; transmembrane emp24 domain trafficking protein 2	4.39
Mon1a	MON1 homolog A (yeast)	4.39
Araf	v-raf murine sarcoma 3611 viral oncogene homolog	4.39
Stxbp6; Gm38487	syntaxin binding protein 6 (amisyn); predicted gene, 38487	4.38
Zfp763	zinc finger protein 763	4.38
Erich6	glutamate rich 6	4.36
Gm3837; Pdcd5	predicted gene 3837; programmed cell death 5	4.34
Nfic	nuclear factor I/C	4.34
Ebna1bp2	EBNA1 binding protein 2	4.34

		4.34
Rundc3b	RUN domain containing 3B	4.33
Apc2	adenomatosis polyposis coli 2	4.32
B130034C11Rik	RIKEN cDNA B130034C11 gene	4.32
Gm22188	predicted gene, 22188 [Source:MGI Symbol;Acc:MGI:5451965]	4.32
Tfdp2	transcription factor Dp 2	4.32
		4.31
Cnp	2,3-cyclic nucleotide 3 phosphodiesterase	4.3
Gm13305; Gm2002; Il11ra2	predicted gene 13305; predicted gene 2002; interleukin 11 receptor, alpha chain 2	4.3
Gm13305; Gm2002; Il11ra2	predicted gene 13305; predicted gene 2002; interleukin 11 receptor, alpha chain 2	4.3
Gm13305; Gm2002; Il11ra2	predicted gene 13305; predicted gene 2002; interleukin 11 receptor, alpha chain 2	4.3
Gm13305; Gm2002; Il11ra2	predicted gene 13305; predicted gene 2002; interleukin 11 receptor, alpha chain 2	4.3
Dennd4b	DENN/MADD domain containing 4B	4.29
Gstz1	glutathione transferase zeta 1 (maleylacetoacetate isomerase)	4.28
Cyp2a22	cytochrome P450, family 2, subfamily a, polypeptide 22	4.28
Id1	inhibitor of DNA binding 1	4.26
Polr1d	polymerase (RNA) I polypeptide D	4.26
Scube1	signal peptide, CUB domain, EGF-like 1	4.25
Tubb2a	tubulin, beta 2A class IIA	4.24
LOC102634459; Gm10552	uncharacterized LOC102634459; predicted gene 10552 [Source:MGI Symbol;Acc:MGI:3642083]	4.23
Ypel5	yippee-like 5 (Drosophila)	4.23
Cstad; Gm31699	CSA-conditional, T cell activation-dependent protein; predicted gene, 31699	4.23
Igkv1-133	immunoglobulin kappa variable 1-133	4.23
LOC102637947; Gm13144	60S acidic ribosomal protein P1-like; predicted gene 13144 [Source:MGI Symbol;Acc:MGI:3651743]	4.23
LOC101056074	zinc finger protein 124-like	4.22
Alg2	asparagine-linked glycosylation 2 (alpha-1,3-mannosyltransferase)	4.22
Tmem258	transmembrane protein 258	4.2
Acsf3	acyl-CoA synthetase family member 3	4.19
Cck	cholecystokinin	4.18
Eef2	eukaryotic translation elongation factor 2	4.16
Hist1h4k	histone cluster 1, H4k	4.16
Cdc42se2	CDC42 small effector 2	4.15

Gm22231	predicted gene, 22231 [Source:MGI Symbol;Acc:MGI:5452008]	4.15
Bcan	brevican	4.14
Krtap4-9	keratin associated protein 4-9	4.13
Cdo1	cysteine dioxygenase 1, cytosolic	4.13
Tcf7l2	transcription factor 7 like 2, T cell specific, HMG box	4.13
Fuz	fuzzy homolog (Drosophila)	4.12
Tmem86a	transmembrane protein 86A	4.12
Tmem176b	transmembrane protein 176B	4.11
1700093J21Rik	RIKEN cDNA 1700093J21 gene [Source:MGI Symbol;Acc:MGI:1921546]	4.09
Wnt4	wingless-type MMTV integration site family, member 4	4.09
		4.09
Prkci	protein kinase C, iota	4.08
Gm16058	predicted gene 16058	4.08
Ndufa13	NADH dehydrogenase (ubiquinone) 1 alpha subcomplex, 13	4.08
Klhl26	kelch-like 26	4.08
Gps1	G protein pathway suppressor 1	4.07
4731419I09Rik	RIKEN cDNA 4731419I09 gene [Source:MGI Symbol;Acc:MGI:3704222]	4.07
Kcna4	potassium voltage-gated channel, shaker-related subfamily, member 4	4.06
Cbl	Casitas B-lineage lymphoma	4.06
		4.06
Rnf115	ring finger protein 115	4.05
Tmf1	TATA element modulatory factor 1	4.04
Sstr2	somatostatin receptor 2	4.03
Chd1	chromodomain helicase DNA binding protein 1	4.03
P2rx7	purinergic receptor P2X, ligand-gated ion channel, 7	4.03
Pdlim3	PDZ and LIM domain 3	4.03
Csf2ra	colony stimulating factor 2 receptor, alpha, low-affinity (granulocyte-macrophage)	4.02
Kif21a	kinesin family member 21A	4.01
Sema3c	sema domain, immunoglobulin domain (Ig), short basic domain, secreted, (semaphorin) 3C	4.01
		4.01
Setdb1	SET domain, bifurcated 1	3.97
Plekha6	pleckstrin homology domain containing, family A member 6	3.96
Mical3	microtubule associated monooxygenase, calponin and LIM domain containing 3	3.96

Rprl2	ribonuclease P RNA-like 2	3.95
Cs	citrate synthase	3.94
Trim43c	tripartite motif-containing 43C	3.94
Ppp2r2a	protein phosphatase 2, regulatory subunit B, alpha	3.93
		3.93
		3.93
Rdh10	retinol dehydrogenase 10 (all-trans)	3.92
Krt33b	keratin 33B	3.92
		3.92
Fam65b	family with sequence similarity 65, member B	3.91
Gm4968	predicted gene 4968 [Source:MGI Symbol;Acc:MGI:3647516]	3.91
4930503L19Rik	RIKEN cDNA 4930503L19 gene	3.9
Gm13363	predicted gene 13363	3.9
Gm24186	predicted gene, 24186 [Source:MGI Symbol;Acc:MGI:5453963]	3.89
Cxxc1	CXXC finger 1 (PHD domain)	3.89
Olfr1269	olfactory receptor 1269	3.89
Rabggta	Rab geranylgeranyl transferase, a subunit	3.88
Ercc1	excision repair cross-complementing rodent repair deficiency, complementation group 1	3.88
Tmcc3	transmembrane and coiled coil domains 3	3.87
Ppp2r5e	protein phosphatase 2, regulatory subunit B, epsilon	3.87
Snx16	sorting nexin 16	3.87
Gm15710	predicted gene 15710 [Source:MGI Symbol;Acc:MGI:3783151]	3.87
Gm23266	predicted gene, 23266 [Source:MGI Symbol;Acc:MGI:5453043]	3.85
Pcp4; Igsf5	Purkinje cell protein 4; immunoglobulin superfamily, member 5	3.83
Ak1	adenylate kinase 1	3.83
Map6	microtubule-associated protein 6	3.83
		3.82
Stxbp3-ps	syntaxin-binding protein 3, pseudogene	3.82
Mir5120	microRNA 5120	3.82
Gm10775	predicted gene 10775 [Source:MGI Symbol;Acc:MGI:3642324]	3.8
Nop10	NOP10 ribonucleoprotein	3.8
Ola1	Obg-like ATPase 1	3.8
Zrsr2	zinc finger (CCCH type), RNA binding motif and serine/arginine rich 2	3.8
Phf23	PHD finger protein 23	3.79
		3.79
Mcee	methylmalonyl CoA epimerase	3.77

Hsd3b3	hydroxy-delta-5-steroid dehydrogenase, 3 beta- and steroid delta-isomerase 3	3.75
		3.74
Ctps	cytidine 5-triphosphate synthase	3.73
Vmn2r35	vomeronasal 2, receptor 35	3.73
Crebbp	CREB binding protein	3.72
H2-K2	histocompatibility 2, K region locus 2	3.72
4933407L21Rik	RIKEN cDNA 4933407L21 gene	3.71
Dio3os	deiodinase, iodothyronine type III, opposite strand	3.71
Dync1li2	dynein, cytoplasmic 1 light intermediate chain 2	3.7
Ighv2-6-8	immunoglobulin heavy variable 2-6-8	3.68
Reep5	receptor accessory protein 5	3.68
Gm22221	predicted gene, 22221 [Source:MGI Symbol;Acc:MGI:5451998]	3.68
		3.68
Gm23965	predicted gene, 23965 [Source:MGI Symbol;Acc:MGI:5453742]	3.67
Gprin3	GPRIN family member 3	3.65
Mrpl30	mitochondrial ribosomal protein L30	3.64
Lmbr1l	limb region 1 like	3.64
Shisa9	shisa family member 9	3.64
Fgf1	fibroblast growth factor 1	3.64
Dtx1	deltex 1 homolog (Drosophila)	3.64
Hsdl2	hydroxysteroid dehydrogenase like 2	3.63
Lrp3	low density lipoprotein receptor-related protein 3	3.63
Grin1os	glutamate receptor, ionotropic, NMDA1 (zeta 1), opposite strand	3.62
4921531C22Rik	RIKEN cDNA 4921531C22 gene	3.62
Gm25703	predicted gene, 25703 [Source:MGI Symbol;Acc:MGI:5455480]	3.62
Mbtps1	membrane-bound transcription factor peptidase, site 1	3.61
Kctd5	potassium channel tetramerisation domain containing 5	3.6
Arpc4	actin related protein 2/3 complex, subunit 4	3.6
Gm26721	predicted gene, 26721	3.6
Hat1	histone aminotransferase 1	3.59
Fam114a1	family with sequence similarity 114, member A1	3.59
		3.59
Ogdhl	oxoglutarate dehydrogenase-like	3.58
Tekt4	tektin 4	3.58
Gm25785	predicted gene, 25785 [Source:MGI Symbol;Acc:MGI:5455562]	3.57

Leo1	Leo1, Paf1/RNA polymerase II complex component, homolog (S. cerevisiae)	3.57
Hist1h2an	histone cluster 1, H2an	3.56
Galnt4	UDP-N-acetyl-alpha-D-galactosamine:polypeptide N-acetylgalactosaminyltransferase 4	3.55
Gm10941	predicted gene 10941	3.55
Slc24a3	solute carrier family 24 (sodium/potassium/calcium exchanger), member 3	3.55
Gm23443	predicted gene, 23443 [Source:MGI Symbol;Acc:MGI:5453220]	3.55
Gm2614	predicted gene 2614	3.54
Gm4609	predicted gene 4609 [Source:MGI Symbol;Acc:MGI:3782792]	3.54
D130040H23Rik	RIKEN cDNA D130040H23 gene	3.54
Gpr137b	G protein-coupled receptor 137B	3.53
Tprkb	Tp53rk binding protein	3.53
Gm9222	predicted gene 9222	3.52
Cd59a	CD59a antigen	3.52
Stk4	serine/threonine kinase 4	3.5
Pinx1	PIN2/TERF1 interacting, telomerase inhibitor 1	3.49
Gm25092	predicted gene, 25092 [Source:MGI Symbol;Acc:MGI:5454869]	3.49
Cwf19l2	CWF19-like 2, cell cycle control (S. pombe)	3.49
Rpl21-ps6	ribosomal protein L21, pseudogene 6	3.46
9330102E08Rik	RIKEN cDNA 9330102E08 gene	3.46
Ero1l	ERO1-like (S. cerevisiae)	3.45
Gm24878	predicted gene, 24878 [Source:MGI Symbol;Acc:MGI:5454655]	3.45
Olfr584	olfactory receptor 584	3.45
Pbx1	pre B cell leukemia homeobox 1	3.44
Fam217b	family with sequence similarity 217, member B	3.44
Syne1	spectrin repeat containing, nuclear envelope 1	3.43
Nrbf2	nuclear receptor binding factor 2	3.43
Camk2n2	calcium/calmodulin-dependent protein kinase II inhibitor 2	3.43
Triqk	triple QxxK/R motif containing	3.43
Asl	argininosuccinate lyase	3.43
Vmn1r159	vomeronasal 1 receptor 159	3.43
LOC105244007; Gm20045	uncharacterized LOC105244007; predicted gene, 20045 [Source:MGI Symbol;Acc:MGI:5012230]	3.43
Stx1a	syntaxin 1A (brain)	3.42
Clec2d	C-type lectin domain family 2, member d	3.42

LOC102633627; Gm7809	tropomyosin alpha-4 chain pseudogene; predicted gene 7809 [Source:MGI Symbol;Acc:MGI:3643362]	3.42
Gm24670	predicted gene, 24670 [Source:MGI Symbol;Acc:MGI:5454447]	3.41
Msn	moesin	3.41
		3.41
Rgs9	regulator of G-protein signaling 9	3.4
Prr14l	proline rich 14-like	3.4
Pold1	polymerase (DNA directed), delta 1, catalytic subunit	3.4
Mrgprx2	MAS-related GPR, member X2	3.4
Arpin	actin-related protein 2/3 complex inhibitor	3.4
Smim7	small integral membrane protein 7	3.4
Slc12a2	solute carrier family 12, member 2	3.39
Gabra5	gamma-aminobutyric acid (GABA) A receptor, subunit alpha 5	3.39
		3.39
4930594O21Rik	RIKEN cDNA 4930594O21 gene	3.38
Krcc1; Mir8112	lysine-rich coiled-coil 1; microRNA 8112	3.38
Nup98	nucleoporin 98	3.38
Galnt7	UDP-N-acetyl-alpha-D-galactosamine: polypeptide N-acetylgalactosaminyltransferase 7	3.38
Dennd6b	DENN/MADD domain containing 6B	3.36
Lig3	ligase III, DNA, ATP-dependent	3.35
Gm24544	predicted gene, 24544 [Source:MGI Symbol;Acc:MGI:5454321]	3.35
Ecsit	ECSIT homolog (Drosophila)	3.35
Gm23123	predicted gene, 23123 [Source:MGI Symbol;Acc:MGI:5452900]	3.34
Gm8995	predicted gene 8995	3.33
Taf1	TAF1 RNA polymerase II, TATA box binding protein (TBP)-associated factor	3.33
Rps10	ribosomal protein S10	3.32
Gm10719	predicted gene 10719 [Source:MGI Symbol;Acc:MGI:3641690]	3.32
Gm22602	predicted gene, 22602 [Source:MGI Symbol;Acc:MGI:5452379]	3.31
Gm5138; Gm2574	predicted gene 5138 [Source:MGI Symbol;Acc:MGI:3779464]; predicted pseudogene 2574 [Source:MGI Symbol;Acc:MGI:3780741]	3.3
Gm5138; Gm2574	predicted gene 5138 [Source:MGI Symbol;Acc:MGI:3779464]; predicted pseudogene 2574 [Source:MGI Symbol;Acc:MGI:3780741]	3.3

Oard1	O-acyl-ADP-ribose deacylase 1	3.29
Ddx39	DEAD (Asp-Glu-Ala-Asp) box polypeptide 39	3.29
Rsph6a	radial spoke head 6 homolog A (Chlamydomonas)	3.28
Nmnat2	nicotinamide nucleotide adenylyltransferase 2	3.27
Ccdc190	coiled-coil domain containing 190	3.27
Gm24898	predicted gene, 24898 [Source:MGI Symbol;Acc:MGI:5454675]	3.27
2510009E07Rik	RIKEN cDNA 2510009E07 gene	3.27
Aldoa	aldolase A, fructose-bisphosphate	3.27
Stat3	signal transducer and activator of transcription 3	3.26
Cygb	cytoglobin	3.26
Gm8016	predicted gene 8016	3.26
Mcmbp	MCM (minichromosome maintenance deficient) binding protein	3.26
Gm2415	predicted gene 2415 [Source:MGI Symbol;Acc:MGI:3780582]	3.26
Cep131	centrosomal protein 131	3.25
		3.25
Gm24942	predicted gene, 24942 [Source:MGI Symbol;Acc:MGI:5454719]	3.24
Cyp27a1	cytochrome P450, family 27, subfamily a, polypeptide 1	3.24
Rbfox3	RNA binding protein, fox-1 homolog (C. elegans) 3	3.24
Gm6498	glyceraldehyde-3-phosphate dehydrogenase pseudogene	3.24
Hsf1	heat shock factor 1	3.24
Pbx2	pre B cell leukemia homeobox 2	3.24
Gm8801	protein phosphatase 1, regulatory subunit 10 pseudogene	3.24
		3.24
Commd7	COMM domain containing 7	3.24
Rhox9	reproductive homeobox 9	3.24
Tmem254b; Tmem254c; Tmem254a	transmembrane protein 254b; transmembrane protein 254c; transmembrane protein 254a	3.23
Gm22497	predicted gene, 22497 [Source:MGI Symbol;Acc:MGI:5452274]	3.23
Ifnl2	interferon lambda 2	3.23
		3.23
Sp2	Sp2 transcription factor	3.22
Ptdss1	phosphatidylserine synthase 1	3.22
Rps24	ribosomal protein S24	3.22
Rsph3b	radial spoke 3B homolog (Chlamydomonas)	3.22
Tmtc1	transmembrane and tetratricopeptide repeat containing 1	3.22

Rfxank	regulatory factor X-associated ankyrin-containing protein	3.22
Gm26675	predicted gene, 26675 [Source:MGI Symbol;Acc:MGI:5477169]	3.21
Rpp21	ribonuclease P 21 subunit	3.21
Gm26564	predicted gene, 26564	3.2
Mir344i	microRNA 344i	3.2
Ncoa7	nuclear receptor coactivator 7	3.19
n-R5s39	nuclear encoded rRNA 5S 39 [Source:MGI Symbol;Acc:MGI:4421884]	3.19
Parva	parvin, alpha	3.19
		3.19
Hmgb1	high mobility group box 1	3.19
Slc23a3	solute carrier family 23 (nucleobase transporters), member 3	3.18
Gm22949	predicted gene, 22949 [Source:MGI Symbol;Acc:MGI:5452726]	3.18
Btbd3; Gm14061	BTB (POZ) domain containing 3; predicted gene 14061	3.18
Chrna7	cholinergic receptor, nicotinic, alpha polypeptide 7	3.18
Gm7676	predicted gene 7676 [Source:MGI Symbol;Acc:MGI:3644053]	3.18
Gm2696	predicted gene 2696	3.17
Aldh5a1	aldhehyde dehydrogenase family 5, subfamily A1	3.17
Snhg11	small nucleolar RNA host gene 11	3.17
Pld3	phospholipase D family, member 3	3.17
Gm8692	predicted gene 8692	3.17
Lactb2	lactamase, beta 2	3.16
Tor2a	torsin family 2, member A	3.16
Atox1	ATX1 (antioxidant protein 1) homolog 1 (yeast)	3.15
Crkl	v-crk sarcoma virus CT10 oncogene homolog (avian)-like	3.15
Fam162a	family with sequence similarity 162, member A	3.15
Tacc2	transforming, acidic coiled-coil containing protein 2	3.15
Gm10717	predicted gene 10717 [Source:MGI Symbol;Acc:MGI:3642031]	3.15
Eif2s3x	eukaryotic translation initiation factor 2, subunit 3, structural gene X-linked	3.15
Slc25a39	solute carrier family 25, member 39	3.14
Il10rb	interleukin 10 receptor, beta	3.14
Elac1	elaC homolog 1 (E. coli)	3.14
Rbfa	ribosome binding factor A	3.14
Aprt	adenine phosphoribosyl transferase	3.14
Tnrc6c	trinucleotide repeat containing 6C	3.13

Ube2k	ubiquitin-conjugating enzyme E2K	3.13
Gm10715	predicted gene 10715 [Source:MGI Symbol;Acc:MGI:3642376]	3.13
Slc44a2	solute carrier family 44, member 2	3.13
Gm26715	predicted gene, 26715	3.12
Dbndd2	dysbindin (dystrobrevin binding protein 1) domain containing 2	3.12
Eno1	enolase 1, alpha non-neuron	3.11
Tpst1	protein-tyrosine sulfotransferase 1	3.11
Hexim2	hexamethylene bis-acetamide inducible 2	3.09
Gm23300	predicted gene, 23300 [Source:MGI Symbol;Acc:MGI:5453077]	3.09
Atad1	ATPase family, AAA domain containing 1	3.09
Lrrc8b	leucine rich repeat containing 8 family, member B	3.09
Tmem175	transmembrane protein 175	3.09
Chrna3	cholinergic receptor, nicotinic, alpha polypeptide 3	3.09
Hist1h3i	histone cluster 1, H3i	3.08
Dopey2	dopey family member 2	3.08
Trim33	tripartite motif-containing 33	3.08
Gm3086	RuvB-like protein 1 pseudogene	3.07
Ctsa	cathepsin A	3.07
Adgrg1	adhesion G protein-coupled receptor G1	3.07
Erdr1	erythroid differentiation regulator 1	3.07
		3.06
Akr1e1	aldo-keto reductase family 1, member E1	3.06
Ugt3a1	UDP glycosyltransferases 3 family, polypeptide A1	3.06
Rpap2	RNA polymerase II associated protein 2	3.06
Rnf169	ring finger protein 169	3.06
Adgrl2	adhesion G protein-coupled receptor L2	3.05
Carkd	carbohydrate kinase domain containing	3.05
Gm23979	predicted gene, 23979 [Source:MGI Symbol;Acc:MGI:5453756]	3.04
Tut1	terminal uridylyl transferase 1, U6 snRNA-specific	3.04
H6pd	hexose-6-phosphate dehydrogenase (glucose 1-dehydrogenase)	3.04
Sowahb	sosondowah ankyrin repeat domain family member B	3.04
Gm22368; Gm8814	predicted gene, 22368 [Source:MGI Symbol;Acc:MGI:5452145]; predicted gene 8814 [Source:MGI Symbol;Acc:MGI:3648632]	3.04
Zfp808	zinc finger protein 80	3.03
Spats2	spermatogenesis associated, serine-rich 2	3.03
Caskin1	CASK interacting protein 1	3.03
Tysnd1	trypsin domain containing 1	3.02

Gm12359	predicted gene 12359	3.02
2210016F16Rik	RIKEN cDNA 2210016F16 gene	3.02
Decr2	2-4-dienoyl-Coenzyme A reductase 2, peroxisomal	3.02
1700037H04Rik	RIKEN cDNA 1700037H04 gene	3.02
Gm20754	predicted gene, 20754	3.02
Dnaaf5	dynein, axonemal, assembly factor 5	3.02
Sumf1	sulfatase modifying factor 1	3.02
Car7	carbonic anhydrase 7	3.02
Mpc2	mitochondrial pyruvate carrier 2	3.01
Gm10566	predicted gene 10566 [Source:MGI Symbol;Acc:MGI:3642220]	3.01
Ssc5d	scavenger receptor cysteine rich family, 5 domains	3.01
Plekho2	pleckstrin homology domain containing, family O member 2	3.01
Chrac1	chromatin accessibility complex 1	3
Rtp1	receptor transporter protein 1	3
D030046N08Rik	RIKEN cDNA D030046N08 gene [Source:MGI Symbol;Acc:MGI:3645234]	3
Olfr519	olfactory receptor 519	3
Dusp10; Gm39725	dual specificity phosphatase 10; predicted gene, 39725	2.99
Pdgfd	platelet-derived growth factor, D polypeptide	2.99
Kank2	KN motif and ankyrin repeat domains 2	2.99
Ankrd23	ankyrin repeat domain 23	2.98
Id2	inhibitor of DNA binding 2	2.98
Gm22750	predicted gene, 22750 [Source:MGI Symbol;Acc:MGI:5452527]	2.98
Lypd6	LY6/PLAUR domain containing 6	2.98
Mrgpra2b	MAS-related GPR, member A2B	2.98
Gm24727	predicted gene, 24727 [Source:MGI Symbol;Acc:MGI:5454504]	2.98
Alyref	Aly/REF export factor	2.97
Speer4cos	spermatogenesis associated glutamate (E)-rich protein 4C, opposite strand transcript	2.97
Colgalt2	collagen beta(1-O)galactosyltransferase 2	2.96
4-Sep	septin 4	2.96
Fbxl3	F-box and leucine-rich repeat protein 3	2.96
Cnnm2	cyclin M2	2.96
Gm2799	predicted gene 2799	2.96
Fthl17b	ferritin, heavy polypeptide-like 17, member B	2.96
Gm24754	predicted gene, 24754 [Source:MGI Symbol;Acc:MGI:5454531]	2.95
Trub2	TruB pseudouridine (psi) synthase homolog 2 (E. coli)	2.95

P2ry12	purinergic receptor P2Y, G-protein coupled 12	2.95
		2.93
Slc8a1	solute carrier family 8 (sodium/calcium exchanger), member 1	2.93
Cgref1	cell growth regulator with EF hand domain 1	2.93
Rny3	RNA, Y3 small cytoplasmic (associated with Ro protein)	2.93
Fam83g	family with sequence similarity 83, member G	2.92
Gm6316	predicted pseudogene 6316 [Source:MGI Symbol;Acc:MGI:3646088]	2.92
4930413F20Rik	RIKEN cDNA 4930413F20 gene	2.92
Tomm5	translocase of outer mitochondrial membrane 5 homolog (yeast)	2.92
Cml3	camello-like 3	2.92
Atp5f1	ATP synthase, H+ transporting, mitochondrial F0 complex, subunit B1	2.91
Ttc13	tetratricopeptide repeat domain 13	2.91
AI414108; Igsf9b	expressed sequence AI414108; immunoglobulin superfamily, member 9B	2.91
Extl3	exostoses (multiple)-like 3	2.9
1700066C05Rik	RIKEN cDNA 1700066C05 gene	2.9
Mt1	metallothionein 1	2.9
Vat1l	vesicle amine transport protein 1 homolog-like (T. californica)	2.9
Gm15114	predicted gene 15114	2.9
Prss40	protease, serine 40	2.89
Fn3k	fructosamine 3 kinase	2.89
Pcdhb12	protocadherin beta 12	2.89
Fstl5	follistatin-like 5	2.89
1500035N22Rik	RIKEN cDNA 1500035N22 gene	2.89
5031425E22Rik	RIKEN cDNA 5031425E22 gene	2.89
Gm4567	predicted gene 4567	2.89
Litaf; Gm9861	LPS-induced TN factor; predicted gene 9861	2.88
Gm25135	predicted gene, 25135 [Source:MGI Symbol;Acc:MGI:5454912]	2.88
Gm6978	predicted gene 6978	2.88
1700030J22Rik	RIKEN cDNA 1700030J22 gene	2.88
Rabif	RAB interacting factor	2.87
		2.87
		2.87
Fdxr	ferredoxin reductase	2.87
Top1mt	DNA topoisomerase 1, mitochondrial	2.87
Vapb	vesicle-associated membrane protein, associated protein B and C	2.87

Rrs1	RRS1 ribosome biogenesis regulator homolog (S. cerevisiae)	2.86
Rimkla	ribosomal modification protein rimK-like family member A	2.86
9330179D12Rik	RIKEN cDNA 9330179D12 gene	2.86
Gm3200	predicted pseudogene 3200 [Source:MGI Symbol;Acc:MGI:3781379]	2.86
Gm11168	predicted gene 11168 [Source:MGI Symbol;Acc:MGI:3779420]	2.86
Zbtb22	zinc finger and BTB domain containing 22	2.85
Rhobtb2	Rho-related BTB domain containing 2	2.84
Tns2	tensin 2	2.84
		2.84
Fuom	fucose mutarotase	2.84
		2.84
Adprhl1	ADP-ribosylhydrolase like 1	2.84
Gm10051	predicted pseudogene 10051	2.83
Shank2	SH3/ankyrin domain gene 2	2.83
Olfr698	olfactory receptor 698	2.83
Gm23388	predicted gene, 23388 [Source:MGI Symbol;Acc:MGI:5453165]	2.83
		2.83
Gm25996	predicted gene, 25996 [Source:MGI Symbol;Acc:MGI:5455773]	2.82
Gm23054	predicted gene, 23054 [Source:MGI Symbol;Acc:MGI:5452831]	2.82
Ttc34	tetratricopeptide repeat domain 34	2.82
Srd5a3	steroid 5 alpha-reductase 3	2.82
Tead1	TEA domain family member 1	2.82
Lmo1	LIM domain only 1	2.82
		2.82
		2.82
		2.82
		2.82
Gm22696	predicted gene, 22696 [Source:MGI Symbol;Acc:MGI:5452473]	2.81
Aif1l	allograft inflammatory factor 1-like	2.81
Slc9a8	solute carrier family 9 (sodium/hydrogen exchanger), member 8	2.81
Rpap1	RNA polymerase II associated protein 1	2.81
Bcl2l15	BCLl2-like 15	2.81
Ppfibp1	PTPRF interacting protein, binding protein 1 (liprin beta 1)	2.81
4930451G09Rik	RIKEN cDNA 4930451G09 gene	2.8

Stub1; Mir3082	STIP1 homology and U-Box containing protein 1; microRNA 3082	2.8
Aco1	aconitase 1	2.8
Agk	acylglycerol kinase	2.8
Krba1	KRAB-A domain containing 1	2.79
Gm16248	predicted gene 16248 [Source:MGI Symbol;Acc:MGI:3826528]	2.78
Gm17104	predicted gene 17104 [Source:MGI Symbol;Acc:MGI:4937931]	2.78
Gpr50	G-protein-coupled receptor 50	2.78
Rdh9	retinol dehydrogenase 9	2.77
Prr3	proline-rich polypeptide 3	2.77
Gm13483	predicted gene 13483	2.77
Trav9d-4	T cell receptor alpha variable 9D-4	2.76
Gm11787	predicted gene 11787	2.76
		2.76
Nr2f6	nuclear receptor subfamily 2, group F, member 6	2.76
Tmod2	tropomodulin 2	2.76
C230029F24Rik	RIKEN cDNA C230029F24 gene	2.75
Tuba3a	tubulin, alpha 3A	2.75
Fcrlb	Fc receptor-like B	2.74
		2.74
Gm12207	predicted gene 12207 [Source:MGI Symbol;Acc:MGI:3702399]	2.74
Gabpa	GA repeat binding protein, alpha	2.74
4930578G10Rik	RIKEN cDNA 4930578G10 gene	2.74
Arrb1	arrestin, beta 1	2.74
Sec23ip	Sec23 interacting protein	2.74
Ip6k1	inositol hexaphosphate kinase 1	2.73
		2.73
		2.73
Dusp23	dual specificity phosphatase 23	2.72
Inpp5j	inositol polyphosphate 5-phosphatase J	2.72
Gm24157	predicted gene, 24157 [Source:MGI Symbol;Acc:MGI:5453934]	2.72
Mir1943	microRNA 1943	2.72
		2.72
		2.71
Gm9736	predicted gene 9736	2.71
Gga3	golgi associated, gamma adaptin ear containing, ARF binding protein 3	2.71
Nacc2	nucleus accumbens associated 2, BEN and BTB (POZ) domain containing	2.71

Suv420h2	suppressor of variegation 4-20 homolog 2 (Drosophila)	2.71
Gm5114	predicted gene 5114	2.71
Snrnp70	small nuclear ribonucleoprotein 70 (U1)	2.71
Gm24103	predicted gene, 24103 [Source:MGI Symbol;Acc:MGI:5453880]	2.71
Rab11a	RAB11A, member RAS oncogene family	2.71
Eya4	eyes absent 4 homolog (Drosophila)	2.7
Sfta2	surfactant associated 2	2.7
Gm24339	predicted gene, 24339 [Source:MGI Symbol;Acc:MGI:5454116]	2.7
Wbp5	WW domain binding protein 5	2.7
		2.7
Arid3a	AT rich interactive domain 3A (BRIGHT-like)	2.69
Syne1	spectrin repeat containing, nuclear envelope 1	2.69
Clns1a	chloride channel, nucleotide-sensitive, 1A	2.69
Lars2	leucyl-tRNA synthetase, mitochondrial	2.69
Zic1	zinc finger protein of the cerebellum 1	2.69
		2.69
Gm11732	predicted gene 11732	2.68
Rom1	rod outer segment membrane protein 1	2.68
Bcas2	breast carcinoma amplified sequence 2	2.68
Tvp23b	trans-golgi network vesicle protein 23B	2.67
Etv1	ets variant 1	2.67
Reep1	receptor accessory protein 1	2.67
9130023H24Rik	RIKEN cDNA 9130023H24 gene	2.67
3300002A11Rik	RIKEN cDNA 3300002A11 gene	2.66
4933406C10Rik	RIKEN cDNA 4933406C10 gene	2.66
Zc3h8	zinc finger CCCH type containing 8	2.66
Zbtb24	zinc finger and BTB domain containing 24	2.65
9330151L19Rik	RIKEN cDNA 9330151L19 gene	2.65
Gm10069	predicted gene 10069	2.65
Atn1	atrophin 1	2.65
Rpl31-ps13	ribosomal protein L31, pseudogene 13	2.64
Zfp598	zinc finger protein 598	2.64
		2.64
Gm25514	predicted gene, 25514 [Source:MGI Symbol;Acc:MGI:5455291]	2.64
Nudt1	nudix (nucleoside diphosphate linked moiety X)-type motif 1	2.64
Gla	galactosidase, alpha	2.64
Trav9-2	T cell receptor alpha variable 9-2	2.63
Tmem254b; Tmem254c; Tmem254a	transmembrane protein 254b; transmembrane protein 254c; transmembrane protein 254a	2.63

Cnot6l	CCR4-NOT transcription complex, subunit 6-like	2.63
Tbc1d9	TBC1 domain family, member 9	2.63
Fam199x	family with sequence similarity 199, X-linked	2.63
1600002K03Rik	RIKEN cDNA 1600002K03 gene	2.62
Gm23826	predicted gene, 23826 [Source:MGI Symbol;Acc:MGI:5453603]	2.62
Gm5088	poly(A)-binding protein, cytoplasmic pseudogene	2.62
Tnfaip8l1	tumor necrosis factor, alpha-induced protein 8-like 1	2.62
Gip	gastric inhibitory polypeptide	2.61
Krt35	keratin 35	2.61
Rgl2	ral guanine nucleotide dissociation stimulator-like 2	2.61
Gm16121	predicted gene 16121 [Source:MGI Symbol;Acc:MGI:3802131]	2.61
Adap1	ArfGAP with dual PH domains 1	2.61
Snora21	small nucleolar RNA, H/ACA box 21	2.6
Tmem106c	transmembrane protein 106C	2.6
Thap6	THAP domain containing 6	2.6
DQ267102	snoRNA DQ267102	2.59
Dok5	docking protein 5	2.59
Dkk2	dickkopf homolog 2 (Xenopus laevis)	2.59
Gnb1	guanine nucleotide binding protein (G protein), beta 1	2.59
Snord64	small nucleolar RNA, C/D box 64	2.59
Gm16178	predicted gene 16178 [Source:MGI Symbol;Acc:MGI:3801783]	2.59
Rpl28; Rpl28-ps1	ribosomal protein L28; ribosomal protein L28, pseudogene 1	2.58
Gm22900	predicted gene, 22900 [Source:MGI Symbol;Acc:MGI:5452677]	2.58
Lrtm2	leucine-rich repeats and transmembrane domains 2	2.58
Ccm2	cerebral cavernous malformation 2	2.57
1600020E01Rik	RIKEN cDNA 1600020E01 gene	2.57
Rpl21-ps14	ribosomal protein L21, pseudogene 14 [Source:MGI Symbol;Acc:MGI:3648110]	2.57
Bnc2	basonuclin 2	2.56
		2.56
Ginm1	glycoprotein integral membrane 1	2.55
Smok2a	sperm motility kinase 2A	2.55
Ints3	integrator complex subunit 3	2.55
Tnfrsf22	tumor necrosis factor receptor superfamily, member 22	2.55
Gm16099	predicted gene 16099 [Source:MGI Symbol;Acc:MGI:3802061]	2.54
		2.54

Rbks	ribokinase	2.54
Gm15929	predicted gene 15929 [Source:MGI Symbol;Acc:MGI:3802090]	2.54
Aurkc; Gm7207	aurora kinase C; predicted gene 7207	2.54
Lyzl4os	lysozyme-like 4, opposite strand	2.54
Stat1	signal transducer and activator of transcription 1	2.53
		2.53
		2.53
Olfr30	olfactory receptor 30	2.53
4921531P14Rik	RIKEN cDNA 4921531P14 gene	2.53
Prss3	protease, serine 3	2.53
		2.53
Creb3l3	cAMP responsive element binding protein 3-like 3	2.52
Pdia6	protein disulfide isomerase associated 6	2.52
Itgb8	integrin beta 8	2.52
		2.52
		2.52
Erich5	glutamate rich 5	2.51
2300005B03Rik	RIKEN cDNA 2300005B03 gene	2.51
Prrt4	proline-rich transmembrane protein 4	2.51
Nell1os	NEL-like 1, opposite strand	2.51
Zfp882; Zfp617	zinc finger protein 882; zinc finger protein 617	2.51
Mir191; Dalrd3	microRNA 191; DALR anticodon binding domain containing 3	2.51
		2.51
		2.51
		2.51
Gm17189	predicted gene 17189 [Source:MGI Symbol;Acc:MGI:4938016]	2.5
Gm24875	predicted gene, 24875 [Source:MGI Symbol;Acc:MGI:5454652]	2.5
Prss36	protease, serine 36	2.5
Trpc1	transient receptor potential cation channel, subfamily C, member 1	2.5
		2.49
Tmem121	transmembrane protein 121	2.49
Zfp395	zinc finger protein 395	2.49
Ptpn3	protein tyrosine phosphatase, non-receptor type 3	2.49
Gm26286	predicted gene, 26286 [Source:MGI Symbol;Acc:MGI:5456063]	2.49
Gm26122	predicted gene, 26122 [Source:MGI Symbol;Acc:MGI:5455899]	2.48
Gm10260	predicted gene 10260 [Source:MGI Symbol;Acc:MGI:3642298]	2.48

Gm15935	predicted gene 15935 [Source:MGI Symbol;Acc:MGI:3801894]	2.48
I830127L07Rik	RIKEN cDNA I830127L07 gene	2.48
E330033B04Rik	RIKEN cDNA E330033B04 gene	2.48
2810403A07Rik	RIKEN cDNA 2810403A07 gene	2.48
Prkcz	protein kinase C, zeta	2.48
Abhd14b	abhydrolase domain containing 14b	2.48
Esx1	extraembryonic, spermatogenesis, homeobox 1	2.48
		2.48
Lefty1	left right determination factor 1	2.47
Arhgap9	Rho GTPase activating protein 9	2.47
		2.47
Gm25274	predicted gene, 25274 [Source:MGI Symbol;Acc:MGI:5455051]	2.47
Olfr166	olfactory receptor 166	2.47
Gm24720	predicted gene, 24720 [Source:MGI Symbol;Acc:MGI:5454497]	2.47
Tes	testis derived transcript	2.47
Rab30	RAB30, member RAS oncogene family	2.47
Adam12; 5830403F22Rik	a disintegrin and metallopeptidase domain 12 (meltrin alpha); RIKEN cDNA 5830403F22 gene	2.47
Pde4c	phosphodiesterase 4C, cAMP specific	2.47
Rhox3h; Rhox3f	reproductive homeobox 3H; reproductive homeobox 3F	2.47
Ighv1-84	immunoglobulin heavy variable 1-84	2.46
Olfr1143	olfactory receptor 1143	2.46
Acad8	acyl-Coenzyme A dehydrogenase family, member 8	2.46
Paip1	polyadenylate binding protein-interacting protein 1	2.45
Pitx1	paired-like homeodomain transcription factor 1	2.45
Gm25466	predicted gene, 25466 [Source:MGI Symbol;Acc:MGI:5455243]	2.45
Gata4	GATA binding protein 4	2.45
Mfsd5	major facilitator superfamily domain containing 5	2.45
Gm15802	predicted gene 15802	2.45
Zbtb12	zinc finger and BTB domain containing 12	2.45
Etv3	ets variant 3	2.45
Szt2	seizure threshold 2	2.45
Pex5	peroxisomal biogenesis factor 5	2.45
LOC105243090; Gm7579	keratin-associated protein 5-5-like; predicted gene 7579 [Source:MGI Symbol;Acc:MGI:3647476]	2.45
Cspp1	centrosome and spindle pole associated protein 1	2.44
Slc43a2	solute carrier family 43, member 2	2.44
Gm23951	predicted gene, 23951 [Source:MGI Symbol;Acc:MGI:5453728]	2.44

S100a11-ps	S100 calcium binding protein A11, pseudogene [Source:MGI Symbol;Acc:MGI:3645720]	2.44
Cdc42ep1	CDC42 effector protein (Rho GTPase binding) 1	2.44
Htr4	5 hydroxytryptamine (serotonin) receptor 4	2.44
Diaph1	diaphanous related formin 1	2.44
		2.44
Imp4	IMP4, U3 small nucleolar ribonucleoprotein, homolog (yeast)	2.43
Cdk4	cyclin-dependent kinase 4	2.43
Skint10	selection and upkeep of intraepithelial T cells 10	2.43
Gm6139	predicted gene 6139 [Source:MGI Symbol;Acc:MGI:3643279]	2.43
Ces2d-ps	carboxylesterase 2D, pseudogene	2.43
		2.42
Grm4	glutamate receptor, metabotropic 4	2.42
Gbp3	guanylate binding protein 3	2.42
Snord73a	small nucleolar RNA, C/D box U73A	2.42
		2.42
Chst8	carbohydrate (N-acetylgalactosamine 4-0) sulfotransferase 8	2.42
AA986860	expressed sequence AA986860	2.41
		2.41
Fgfr3	fibroblast growth factor receptor 3	2.41
Olfr513	olfactory receptor 513	2.41
Sgf29	SAGA complex associated factor 29	2.41
Plekhg2	pleckstrin homology domain containing, family G (with RhoGef domain) member 2	2.41
Sbno2	strawberry notch homolog 2 (Drosophila)	2.4
Rb1	retinoblastoma 1	2.4
Gdpd3	glycerophosphodiester phosphodiesterase domain containing 3	2.4
Gm24333	predicted gene, 24333 [Source:MGI Symbol;Acc:MGI:5454110]	2.4
Tmem115	transmembrane protein 115	2.4
Gm14549	predicted gene 14549 [Source:MGI Symbol;Acc:MGI:3705175]	2.4
Gm7609	predicted pseudogene 7609	2.39
Trim80	tripartite motif-containing 80	2.39
Zfp174	zinc finger protein 174	2.38
Lrsam1	leucine rich repeat and sterile alpha motif containing 1	2.38
Pigk	phosphatidylinositol glycan anchor biosynthesis, class K	2.38

Rps27a; Rps27a-ps2	ribosomal protein S27A; ribosomal protein S27A, pseudogene 2	2.38
Zfp366	zinc finger protein 366	2.37
Ring1	ring finger protein 1	2.37
Dnd1	dead end homolog 1 (zebrafish)	2.37
		2.37
Gm26516	predicted gene, 26516 [Source:MGI Symbol;Acc:MGI:5477010]	2.37
Gm13058	predicted gene 13058 [Source:MGI Symbol;Acc:MGI:3650002]	2.37
Cfap46	cilia and flagella associated protein 46	2.37
Hmgb2	high mobility group box 2	2.37
Tmem170	transmembrane protein 170	2.37
Slfn2	schlafen 2	2.36
Asb13	ankyrin repeat and SOCS box-containing 13	2.36
		2.36
CK137956	cDNA sequence CK137956	2.36
Gm16049	predicted gene 16049	2.35
Elmsan1	ELM2 and Myb/SANT-like domain containing 1	2.34
Zfp759	zinc finger protein 759	2.34
Psors1c2	psoriasis susceptibility 1 candidate 2 (human)	2.34
Crocc	ciliary rootlet coiled-coil, rootletin	2.34
Plpp4	phospholipid phosphatase 4	2.34
Sct	secretin	2.34
2210011C24Rik; Mir1199	RIKEN cDNA 2210011C24 gene; microRNA 1199	2.34
4921534H16Rik	RIKEN cDNA 4921534H16 gene	2.34
Slc1a4	solute carrier family 1 (glutamate/neutral amino acid transporter), member 4	2.33
Gm14488	predicted gene 14488	2.33
LOC102637278; Gm13017	uncharacterized LOC102637278; predicted gene 13017 [Source:MGI Symbol;Acc:MGI:3650528]	2.33
1700012B09Rik	RIKEN cDNA 1700012B09 gene	2.33
Igfbp5	insulin-like growth factor binding protein 5	2.32
Gm8126	predicted gene 8126 [Source:MGI Symbol;Acc:MGI:3646861]	2.32
Tmem260	transmembrane protein 260	2.32
		2.32
Scube3	signal peptide, CUB domain, EGF-like 3	2.32
		2.32
4931403M11Rik	RIKEN cDNA 4931403M11 gene	2.31
Ifitm7	interferon induced transmembrane protein 7	2.31
Gm24078	predicted gene, 24078 [Source:MGI Symbol;Acc:MGI:5453855]	2.31

Gm12371	predicted gene 12371	2.31
Skint7	selection and upkeep of intraepithelial T cells 7	2.31
Klhl17	kelch-like 17	2.31
Rpl32l	ribosomal protein L32-like	2.3
Gm24900	predicted gene, 24900 [Source:MGI Symbol;Acc:MGI:5454677]	2.3
Snx5	sorting nexin 5	2.3
Laptm5	lysosomal-associated protein transmembrane 5	2.3
Vmn2r36	vomeronasal 2, receptor 36	2.3
1700001J11Rik	ring finger protein 19A pseudogene	2.3
Gm5039	eukaryotic translation initiation factor 1A pseudogene	2.29
Txndc11	thioredoxin domain containing 11	2.29
T2; T; Sft2d1	brachyury 2; brachyury; SFT2 domain containing 1	2.29
l7Rn6	lethal, Chr 7, Rinchik 6	2.29
		2.29
		2.28
Vmn2r88	vomeronasal 2, receptor 88	2.28
Gm4544	predicted gene 4544	2.28
		2.28
Chn1os3	chimerin 1, opposite strand 3	2.28
Gm35553	predicted gene, 35553	2.28
Ccng2	cyclin G2	2.28
Nkx3-2	NK3 homeobox 2	2.28
Fan1	FANCD2/FANCI-associated nuclease 1	2.28
Grtp1	GH regulated TBC protein 1	2.28
Clcn5	chloride channel 5	2.28
		2.28
Chit1	chitinase 1 (chitotriosidase)	2.27
Tmem41a	transmembrane protein 41a	2.27
Mir669a-3	microRNA 669a-3	2.27
1700026L06Rik	RIKEN cDNA 1700026L06 gene	2.27
Slc27a3	solute carrier family 27 (fatty acid transporter), member 3	2.27
Paqr7	progestin and adipoQ receptor family member VII	2.27
Mir3095	microRNA 3095	2.27
		2.27
6330408A02Rik	RIKEN cDNA 6330408A02 gene	2.27
Pgf	placental growth factor	2.26
Gm3424	predicted gene 3424	2.26
Syt10	synaptotagmin X	2.26
1700010I14Rik	RIKEN cDNA 1700010I14 gene	2.26
Zswim3	zinc finger SWIM-type containing 3	2.26
Tram1l1	translocation associated membrane protein 1-like 1	2.26

Rpl6	ribosomal protein L6	2.26
Det1	de-etiolated homolog 1 (Arabidopsis)	2.26
Gm4559	predicted gene 4559	2.26
Eaf1	ELL associated factor 1	2.25
Pnoc	prepronociceptin	2.25
Gm25967	predicted gene, 25967 [Source:MGI Symbol;Acc:MGI:5455744]	2.25
Olfr1424	olfactory receptor 1424	2.25
Ermp1	endoplasmic reticulum metallopeptidase 1	2.25
1110051M20Rik	RIKEN cDNA 1110051M20 gene	2.25
Slc10a4	solute carrier family 10 (sodium/bile acid cotransporter family), member 4	2.25
Upp1	uridine phosphorylase 1	2.24
Gm12349	predicted gene 12349	2.24
Pdlim1	PDZ and LIM domain 1 (elfin)	2.24
Gm15471	predicted gene 15471	2.24
Acta1	actin, alpha 1, skeletal muscle	2.24
Gm11939	predicted gene 11939 [Source:MGI Symbol;Acc:MGI:3651443]	2.23
Vsx2	visual system homeobox 2	2.23
Ighv2-4	immunoglobulin heavy variable V2-4	2.23
Gm20405	predicted gene 20405 [Source:MGI Symbol;Acc:MGI:5141870]	2.23
H2-Bl; Gm8909; Gm10499	histocompatibility 2, blastocyst; predicted gene 8909; predicted gene 10499	2.23
4930429F11Rik	RIKEN cDNA 4930429F11 gene	2.23
Gm14266	predicted gene 14266 [Source:MGI Symbol;Acc:MGI:3649850]	2.23
Gm13380	predicted gene 13380 [Source:MGI Symbol;Acc:MGI:3649647]	2.23
Gm12603	predicted gene 12603	2.23
Hadhb	hydroxyacyl-Coenzyme A dehydrogenase/3-ketoacyl-Coenzyme A thiolase/enoyl-Coenzyme A hydratase (trifunctional protein), beta subunit	2.23
Chmp3	charged multivesicular body protein 3	2.23
Mir680-1	microRNA 680-1	2.23
Mesp2	mesoderm posterior 2	2.23
Rab37	RAB37, member RAS oncogene family	2.22
Senp5	SUMO/sentrin specific peptidase 5	2.22
Gm14264	predicted gene 14264	2.22
Ror1	receptor tyrosine kinase-like orphan receptor 1	2.22
		2.22
Gm15991	predicted gene 15991	2.22
		2.21

Gm11562	predicted gene 11562	2.21
Enthd2	ENTH domain containing 2	2.21
Rpph1	ribonuclease P RNA component H1	2.21
Tprn	taperin	2.21
Slc52a3	solute carrier protein family 52, member 3	2.21
Defa-rs1	defensin, alpha, related sequence 1	2.21
Trav12-1	T cell receptor alpha variable 12-1	2.2
Dnajc10	DnaJ (Hsp40) homolog, subfamily C, member 10	2.2
Gm23830	predicted gene, 23830 [Source:MGI Symbol;Acc:MGI:5453607]	2.2
Ccdc106	coiled-coil domain containing 106	2.2
Tns4	tensin 4	2.19
Gmnn	geminin	2.19
Ripk3	receptor-interacting serine-threonine kinase 3	2.19
Fzd8	frizzled homolog 8 (Drosophila)	2.19
B3gat3	beta-1,3-glucuronyltransferase 3 (glucuronosyltransferase I)	2.19
Ppp1r26	protein phosphatase 1, regulatory subunit 26	2.19
Mir695	microRNA 695	2.19
Gm23321	predicted gene, 23321 [Source:MGI Symbol;Acc:MGI:5453098]	2.19
		2.19
Gm20257	caspase 8 pseudogene	2.18
Rgs8	regulator of G-protein signaling 8	2.18
Scrn2	secernin 2	2.18
Mir3091	microRNA 3091	2.18
Evx2	even skipped homeotic gene 2 homolog	2.18
Metap1	methionyl aminopeptidase 1	2.18
Igfbpl1	insulin-like growth factor binding protein-like 1	2.18
Gm15411; 1810059H22Rik	predicted gene 15411; RIKEN cDNA 1810059H22 gene	2.18
Zfp36l3	zinc finger protein 36, C3H type-like 3	2.18
Npb	neuropeptide B	2.17
Slc34a1	solute carrier family 34 (sodium phosphate), member 1	2.17
Gm14258	predicted gene 14258 [Source:MGI Symbol;Acc:MGI:3652141]	2.17
Ints8	integrator complex subunit 8	2.17
Gm1661	predicted gene 1661	2.17
4933413G19Rik	RIKEN cDNA 4933413G19 gene	2.17
Mir3109	microRNA 3109	2.17
Rbm10	RNA binding motif protein 10	2.17
Gm7467	predicted gene 7467	2.17
Trav2	T cell receptor alpha variable 2	2.16

		2.16
Gm13391	predicted gene 13391	2.16
Tmem144	transmembrane protein 144	2.16
Gm12496	predicted gene 12496	2.16
D130007C19Rik; Gm40240	RIKEN cDNA D130007C19 gene; predicted gene, 40240	2.16
Pign	phosphatidylinositol glycan anchor biosynthesis, class N	2.15
Btbd11	BTB (POZ) domain containing 11	2.15
LOC105245161; LOC105247081; 4930432B10Rik	leucine-rich repeat extensin-like protein 5; atherin-like; RIKEN cDNA 4930432B10 gene [Source:MGI Symbol;Acc:MGI:1921869]	2.15
		2.15
Srfbp1	serum response factor binding protein 1	2.15
Gm16299	predicted gene 16299	2.15
Gm25368	predicted gene, 25368 [Source:MGI Symbol;Acc:MGI:5455145]	2.15
AA387200	expressed sequence AA387200	2.15
Ttc39aos1	Ttc39a opposite strand RNA 1	2.15
Smok3b; Smok3a	sperm motility kinase 3B; sperm motility kinase 3A	2.15
Pbx4	pre B cell leukemia homeobox 4	2.15
Gm29692	predicted gene, 29692	2.15
Mettl13	methyltransferase like 13	2.14
Rragb	Ras-related GTP binding B	2.14
Snord11	small nucleolar RNA, C/D box 11	2.13
Rpl3	ribosomal protein L3	2.13
Fitm2	fat storage-inducing transmembrane protein 2	2.13
Gm14455	predicted gene 14455 [Source:MGI Symbol;Acc:MGI:3711286]	2.13
Gm9372	predicted gene 9372 [Source:MGI Symbol;Acc:MGI:3645996]	2.13
Gbp5	guanylate binding protein 5	2.13
Acnat2	acyl-coenzyme A amino acid N-acyltransferase 2	2.13
Olfr541	olfactory receptor 541	2.13
Gm8482	predicted pseudogene 8482 [Source:MGI Symbol;Acc:MGI:3647858]	2.13
Gdap1	ganglioside-induced differentiation-associated-protein 1	2.12
Samd5	sterile alpha motif domain containing 5	2.12
AU016765	expressed sequence AU016765	2.12
Adal	adenosine deaminase-like	2.12
Smtnl1	smoothelin-like 1	2.12
Adam26b	a disintegrin and metallopeptidase domain 26B	2.12
Amelx	amelogenin, X-linked	2.12

Slc14a1	solute carrier family 14 (urea transporter), member 1	2.11
Rad9a	RAD9 homolog A	2.11
Phxr1	per-hexamer repeat gene 1	2.11
Gm24997	predicted gene, 24997 [Source:MGI Symbol;Acc:MGI:5454774]	2.11
Nova2	neuro-oncological ventral antigen 2	2.11
Galnt2	UDP-N-acetyl-alpha-D-galactosamine:polypeptide N-acetylgalactosaminyltransferase 2	2.11
BC065403	cDNA sequence BC065403	2.11
		2.11
LOC102639958	uncharacterized LOC102639958	2.11
Tex14	testis expressed gene 14	2.1
Krt36	keratin 36	2.1
Cox6c	cytochrome c oxidase subunit VIc	2.1
Gm41568; Gm38585	predicted gene, 41568; predicted gene, 38585	2.1
1500015A07Rik	RIKEN cDNA 1500015A07 gene	2.1
Nppb	natriuretic peptide type B	2.1
Olfr613	olfactory receptor 613	2.1
Hpn	hepsin	2.1
Gm22173	predicted gene, 22173 [Source:MGI Symbol;Acc:MGI:5451950]	2.1
Gpr45	G protein-coupled receptor 45	2.09
LOC102635990; 4932415D10Rik	uncharacterized LOC102635990; RIKEN cDNA 4932415D10 gene [Source:MGI Symbol;Acc:MGI:3045298]	2.09
Ighg3	Immunoglobulin heavy constant gamma 3	2.09
Smyd1; Gm38828	SET and MYND domain containing 1; predicted gene, 38828	2.09
		2.09
4933402J07Rik	RIKEN cDNA 4933402J07 gene	2.09
Gm24212	predicted gene, 24212 [Source:MGI Symbol;Acc:MGI:5453989]	2.09
		2.09
		2.09
		2.09
Gm5176	high mobility group box 2 pseudogene	2.08
Tha1	threonine aldolase 1	2.08
Gm9946	predicted gene 9946	2.08
Ighv1-72	immunoglobulin heavy variable 1-72	2.07
Kbtbd4	kelch repeat and BTB (POZ) domain containing 4	2.07
Fxyd3	FXYD domain-containing ion transport regulator 3	2.07
LOC102636530	ubiquitin-like protein 5	2.07
Ric8b	resistance to inhibitors of cholinesterase 8 homolog B (C. elegans)	2.06

Olfr1393	olfactory receptor 1393	2.06
Mir539	microRNA 539	2.06
1700121N20Rik	RIKEN cDNA 1700121N20 gene	2.06
Fam159b	family with sequence similarity 159, member B	2.06
Gm5476	type II keratin Kb17P pseudogene	2.06
Pcgf5	polycomb group ring finger 5	2.06
Catsper4	cation channel, sperm associated 4	2.06
		2.06
Vmn2r50	vomeronasal 2, receptor 50	2.06
Gm25906	predicted gene, 25906 [Source:MGI Symbol;Acc:MGI:5455683]	2.06
Med17	mediator complex subunit 17	2.06
Gm17361	predicted gene, 17361	2.06
Gm32497	predicted gene, 32497	2.06
Gm24988	predicted gene, 24988 [Source:MGI Symbol;Acc:MGI:5454765]	2.05
Gm13387	predicted gene 13387	2.05
Tmem56	transmembrane protein 56	2.05
Tas2r139	taste receptor, type 2, member 139	2.05
Rnf103	ring finger protein 103	2.05
5033428I22Rik	RIKEN cDNA 5033428I22 gene	2.05
Mir362	microRNA 362	2.05
Fam160b2	family with sequence similarity 160, member B2	2.04
Gm11457	predicted gene 11457	2.04
Gm22795	predicted gene, 22795 [Source:MGI Symbol;Acc:MGI:5452572]	2.04
Vmn2r69	vomeronasal 2, receptor 69	2.04
LOC102636777; Gm12665	uncharacterized LOC102636777; predicted gene 12665 [Source:MGI Symbol;Acc:MGI:3652165]	2.03
Gm23579	predicted gene, 23579 [Source:MGI Symbol;Acc:MGI:5453356]	2.03
Gm17750	predicted gene, 17750	2.03
Prdm9	PR domain containing 9	2.03
Gm13415	predicted gene 13415	2.03
Gm25434	predicted gene, 25434 [Source:MGI Symbol;Acc:MGI:5455211]	2.03
Sfi1; Drg1	Sfi1 homolog, spindle assembly associated (yeast); developmentally regulated GTP binding protein 1	2.02
Gm3839	predicted pseudogene 3839 [Source:MGI Symbol;Acc:MGI:3782011]	2.02
A4galt	alpha 1,4-galactosyltransferase	2.02
Tmem114	transmembrane protein 114	2.02
Mbd1	methyl-CpG binding domain protein 1	2.02
Olfm1	olfactomedin 1	2.02

Atg4d	autophagy related 4D, cysteine peptidase	2.02
Cahm	colon adenocarcinoma hypermethylated RNA	2.01
Bpifa2	BPI fold containing family A, member 2	2.01
Lce1k	late cornified envelope 1K	2.01
Nans	N-acetylneuraminic acid synthase (sialic acid synthase)	2.01
Gm12682	predicted gene 12682 [Source:MGI Symbol;Acc:MGI:3650643]	2.01
Gm26264	predicted gene, 26264 [Source:MGI Symbol;Acc:MGI:5456041]	2.01
Vmn2r46	vomeronasal 2, receptor 46	2.01
Vmn2r46	vomeronasal 2, receptor 46	2.01
Tk2	thymidine kinase 2, mitochondrial	2.01
Gm23320	predicted gene, 23320 [Source:MGI Symbol;Acc:MGI:5453097]	2.01
Gm26151	predicted gene, 26151 [Source:MGI Symbol;Acc:MGI:5455928]	2.01
Mir376b	microRNA 376b	-31.31
Snord61	small nucleolar RNA, C/D box 61	-28.79
Gm26347	predicted gene, 26347 [Source:MGI Symbol;Acc:MGI:5456124]	-18.09
Atxn7l1	ataxin 7-like 1	-17.28
Mir329	microRNA 329	-15.77
DQ267100; Rian	snoRNA DQ267100; RNA imprinted and accumulated in nucleus	-14.82
Rabac1	Rab acceptor 1 (prenylated)	-14.37
Gm2260; Gm2274	predicted gene 2260; predicted gene 2274	-13.4
Gm2260; Gm2274	predicted gene 2260; predicted gene 2274	-13.4
Emc7	ER membrane protein complex subunit 7	-12.84
Snord65	small nucleolar RNA, C/D box 65	-12.82
Gm22858	predicted gene, 22858 [Source:MGI Symbol;Acc:MGI:5452635]	-11.79
		-11.75
15-Sep	selenoprotein	-11.67
Psmb4	proteasome (prosome, macropain) subunit, beta type 4	-10.56
Arl15	ADP-ribosylation factor-like 15	-9.8
Fam160a1; Arfip1	family with sequence similarity 160, member A1; ADP-ribosylation factor interacting protein 1	-9.26
Syne1	spectrin repeat containing, nuclear envelope 1	-8.72
Tmem196	transmembrane protein 196	-8.62
Ube2d2a	ubiquitin-conjugating enzyme E2D 2A	-8.55
Slc7a5	solute carrier family 7 (cationic amino acid transporter, y+ system), member 5	-8.22

Npy	neuropeptide Y	-8
Mir668	microRNA 668	-7.86
Gm25635	predicted gene, 25635 [Source:MGI Symbol;Acc:MGI:5455412]	-7.58
Nop56	NOP56 ribonucleoprotein	-7.38
Ccng1	cyclin G1	-7.35
Atp6ap2	ATPase, H+ transporting, lysosomal accessory protein 2	-7.22
Itm2b	integral membrane protein 2B	-7.15
Gm23546	predicted gene, 23546 [Source:MGI Symbol;Acc:MGI:5453323]	-7.12
Max	Max protein	-7.11
Nek7	NIMA (never in mitosis gene a)-related expressed kinase 7	-7.11
Gm21399	predicted gene, 21399 [Source:MGI Symbol;Acc:MGI:5434754]	-6.91
Rbm7	RNA binding motif protein 7	-6.77
Scrg1	scrapie responsive gene 1	-6.54
Snapc3	small nuclear RNA activating complex, polypeptide 3	-6.28
Mapk8	mitogen-activated protein kinase 8	-6.18
Zc4h2	zinc finger, C4H2 domain containing	-6.16
Ube2d3	ubiquitin-conjugating enzyme E2D 3	-6
Wdr82	WD repeat domain containing 82	-5.97
Arl8b	ADP-ribosylation factor-like 8B	-5.69
Mir3075	microRNA 3075	-5.64
Gm16357	predicted gene 16357	-5.63
Zmym2	zinc finger, MYM-type 2	-5.47
		-5.47
		-5.47
Muc3a	mucin 3A, cell surface associated	-5.33
LOC105243690; Gm14681	nucleophosmin-like; predicted gene 14681 [Source:MGI Symbol;Acc:MGI:3705734]	-5.24
Eny2	enhancer of yellow 2 homolog (Drosophila)	-5.2
1810037I17Rik	RIKEN cDNA 1810037I17 gene	-5.17
Ccdc159	coiled-coil domain containing 159	-5.16
Ccnt2	cyclin T2	-4.91
Cpeb2	cytoplasmic polyadenylation element binding protein 2	-4.85
Tctex1d2	Tctex1 domain containing 2	-4.85
Rhoa	ras homolog gene family, member A	-4.83
Bsg	basigin	-4.79
Sdf2	stromal cell derived factor 2	-4.79
Smim11	small integral membrane protein 11	-4.76

Cox8a	cytochrome c oxidase subunit VIIIa	-4.7
Rnf7	ring finger protein 7	-4.66
Dgka	diacylglycerol kinase, alpha	-4.65
Usp33	ubiquitin specific peptidase 33	-4.62
Slc35g2	solute carrier family 35, member G2	-4.58
Ddx5	DEAD (Asp-Glu-Ala-Asp) box polypeptide 5	-4.52
Ccdc25	coiled-coil domain containing 25	-4.49
Cdc26	cell division cycle 26	-4.48
Gm23644	predicted gene, 23644 [Source:MGI Symbol;Acc:MGI:5453421]	-4.38
4933431E20Rik	RIKEN cDNA 4933431E20 gene	-4.38
Churc1; Fntb	churchill domain containing 1; farnesyltransferase, CAAX box, beta	-4.3
Chst1	carbohydrate (keratan sulfate Gal-6) sulfotransferase 1	-4.29
Taf1d	TATA box binding protein (Tbp)-associated factor, RNA polymerase I, D	-4.25
Tmem59l	transmembrane protein 59-like	-4.23
Gm2506	predicted gene 2506	-4.21
Gm2506	predicted gene 2506	-4.21
Gm2506	predicted gene 2506	-4.21
Slc37a3	solute carrier family 37 (glycerol-3-phosphate transporter), member 3	-4.19
Prpf18	PRP18 pre-mRNA processing factor 18 homolog (yeast)	-4.15
Slc17a5	solute carrier family 17 (anion/sugar transporter), member 5	-4.14
Gyg	glycogenin	-4.11
Stx6	syntaxin 6	-4.07
		-4.07
Btf3l4	basic transcription factor 3-like 4	-4.02
Eif3d	eukaryotic translation initiation factor 3, subunit D	-4
Gm24671	predicted gene, 24671 [Source:MGI Symbol;Acc:MGI:5454448]	-3.97
Peak1	pseudopodium-enriched atypical kinase 1	-3.93
Large	like-glycosyltransferase	-3.9
Ldb2	LIM domain binding 2	-3.78
Usp9x	ubiquitin specific peptidase 9, X chromosome	-3.71
n-R5s80	nuclear encoded rRNA 5S 80 [Source:MGI Symbol;Acc:MGI:4421928]	-3.7
Psma1	proteasome (prosome, macropain) subunit, alpha type 1	-3.66
Pfn1	profilin 1	-3.56

Lrfn5	leucine rich repeat and fibronectin type III domain containing 5	-3.52
Jkamp	JNK1/MAPK8-associated membrane protein	-3.52
Frg1	FSHD region gene 1	-3.47
Sft2d1	SFT2 domain containing 1	-3.47
Polr1c	polymerase (RNA) I polypeptide C	-3.46
Gm13770	predicted gene 13770 [Source:MGI Symbol;Acc:MGI:3651719]	-3.46
Taf1b	TATA box binding protein (Tbp)-associated factor, RNA polymerase I, B	-3.38
Sh3gl3	SH3-domain GRB2-like 3	-3.32
Gm25432	predicted gene, 25432	-3.3
Sub1	SUB1 homolog (S. cerevisiae)	-3.27
Slc25a11	solute carrier family 25 (mitochondrial carrier oxoglutarate carrier), member 11	-3.25
Slc25a28	solute carrier family 25, member 28	-3.23
Mtpn	myotrophin	-3.23
Ubap1	ubiquitin-associated protein 1	-3.22
Pcdh7	protocadherin 7	-3.21
Sobp	sine oculis-binding protein homolog (Drosophila)	-3.2
Gm23301	predicted gene, 23301 [Source:MGI Symbol;Acc:MGI:5453078]	-3.2
Cops6	COP9 (constitutive photomorphogenic) homolog, subunit 6 (Arabidopsis thaliana)	-3.18
n-R5s8	nuclear encoded rRNA 5S 8 [Source:MGI Symbol;Acc:MGI:4421742]	-3.18
Amd1	S-adenosylmethionine decarboxylase 1	-3.17
Dph6	diphthamine biosynthesis 6	-3.13
Rnf5	ring finger protein 5	-3.11
Mir1898	microRNA 1898	-3.11
Gm25581	predicted gene, 25581 [Source:MGI Symbol;Acc:MGI:5455358]	-3.11
Rpl4	ribosomal protein L4	-3.09
Aldoc	aldolase C, fructose-bisphosphate	-3.08
Ift22	intraflagellar transport 22	-3.08
Ddx6	DEAD (Asp-Glu-Ala-Asp) box polypeptide 6	-3.08
Skp1a	S-phase kinase-associated protein 1A	-3.01
Sv2b	synaptic vesicle glycoprotein 2 b	-2.9
2310057M21Rik	RIKEN cDNA 2310057M21 gene	-2.79
Rars	arginyl-tRNA synthetase	-2.78
Clock	circadian locomotor output cycles kaput	-2.76
Gm25147	predicted gene, 25147 [Source:MGI Symbol;Acc:MGI:5454924]	-2.74
Ate1	arginyltransferase 1	-2.72

Gdpd1	glycerophosphodiester phosphodiesterase domain containing 1	-2.72
Yipf5	Yip1 domain family, member 5	-2.72
Tceb1	transcription elongation factor B (SIII), polypeptide 1	-2.67
Ddx26b	DEAD/H (Asp-Glu-Ala-Asp/His) box polypeptide 26B	-2.65
Ranbp9	RAN binding protein 9	-2.64
		-2.58
E330009J07Rik	RIKEN cDNA E330009J07 gene	-2.54
Insig1	insulin induced gene 1	-2.53
Bap1	Brca1 associated protein 1	-2.48
Sst	somatostatin	-2.47
Katna1	katanin p60 (ATPase-containing) subunit A1	-2.43
Napb	N-ethylmaleimide sensitive fusion protein attachment protein beta	-2.43
Mlc1	megalencephalic leukoencephalopathy with subcortical cysts 1 homolog (human)	-2.37
Scn2a1	sodium channel, voltage-gated, type II, alpha 1	-2.32
Bbip1	BBSome interacting protein 1	-2.27
Gm1043	predicted gene 1043	-2.23
Btrc	beta-transducin repeat containing protein	-2.22
Psmc1	protease (prosome, macropain) 26S subunit, ATPase 1	-2.16
S100a1	S100 calcium binding protein A1	-2.15
Rgs7	regulator of G protein signaling 7	-2.1
Zfp799	zinc finger protein 799	-2.09
1700025G04Rik	RIKEN cDNA 1700025G04 gene	-2.03
Zfp131	zinc finger protein 131	-2.01

Table S5: Differentially expressed genes in Wester diet (WD) fed LDL-R -/- mice when compared to control diet (CD) fed WT mice.

Gene Symbol	Gene Description	Fold Change
		431.72
Gm24056	predicted gene, 24056 [Source:MGI Symbol;Acc:MGI:5453833]	113.44
Gm25357	predicted gene, 25357 [Source:MGI Symbol;Acc:MGI:5455134]	100.22
Mir5125; Srrm2	microRNA 5125; serine/arginine repetitive matrix 2	75.88
Gm24627	predicted gene, 24627 [Source:MGI Symbol;Acc:MGI:5454404]	58.13
Gm22962	predicted gene, 22962 [Source:MGI Symbol;Acc:MGI:5452739]	45.71
Mir340	microRNA 340	44.23
Gm24009	predicted gene, 24009 [Source:MGI Symbol;Acc:MGI:5453786]	38.45
Mir505	microRNA 505	35.88
Gm24876	predicted gene, 24876 [Source:MGI Symbol;Acc:MGI:5454653]	28.59
Snord16a	small nucleolar RNA, C/D box 16A	26.26
Gm22578	predicted gene, 22578 [Source:MGI Symbol;Acc:MGI:5452355]	25.73
Rgs9	regulator of G-protein signaling 9	25.3
Gm22067	predicted gene, 22067 [Source:MGI Symbol;Acc:MGI:5451844]	19.84
Gm4887	predicted gene 4887	17.87
Eif4a1	eukaryotic translation initiation factor 4A1	16.48
Mir487b	microRNA 487b	15.46
Gm10293	predicted pseudogene 10293 [Source:MGI Symbol;Acc:MGI:3704216]	14.29
Snora23	small nucleolar RNA, H/ACA box 23	14.28
		14.27
Peg10	paternally expressed 10	13.99
Gng7	guanine nucleotide binding protein (G protein), gamma 7	13.88
LOC105245453; Gm2399	nidogen-1-like; predicted gene 2399 [Source:MGI Symbol;Acc:MGI:3780567]	13.88
mt-Tv	mitochondrially encoded tRNA valine [Source:MGI Symbol;Acc:MGI:102472]	13.72
DQ267102	snoRNA DQ267102	13.43
		12.32
		12.32

Gm22957	predicted gene, 22957 [Source:MGI Symbol;Acc:MGI:5452734]	11.98
Anp32b-ps1	Bacidic (leucine-rich) nuclear phosphoprotein 32 family, member B, pseudogene 1	11.93
Nrbp2	nuclear receptor binding protein 2	11
Gm25788	predicted gene, 25788 [Source:MGI Symbol;Acc:MGI:5455565]	10.91
AF357425	snoRNA AF357425	10.25
D4Ertd617e	DNA segment, Chr 4, ERATO Doi 617, expressed	9.82
Mag	myelin-associated glycoprotein	9.71
		9.62
Gm23508; Rian	predicted gene, 23508 [Source:MGI Symbol;Acc:MGI:5453285]; RNA imprinted and accumulated in nucleus [Source:MGI Symbol;Acc:MGI:1922995]	9.62
Ostc	oligosaccharyltransferase complex subunit	9.41
Ppp1r1b	protein phosphatase 1, regulatory (inhibitor) subunit 1B	9.19
		8.91
Copz2	coatomer protein complex, subunit zeta 2	8.88
Pde1b	phosphodiesterase 1B, Ca2+-calmodulin dependent	8.71
Szrd1	SUZ RNA binding domain containing 1	8.51
Rps11-ps1	ribosomal protein S11, pseudogene 1	8.5
Gm5476	type II keratin Kb17P pseudogene	7.96
Syne1	spectrin repeat containing, nuclear envelope 1	7.71
Gm23209	predicted gene, 23209 [Source:MGI Symbol;Acc:MGI:5452986]	7.7
Arhgdia	Rho GDP dissociation inhibitor (GDI) alpha	7.65
Gm5879	predicted gene 5879	7.6
		7.53
Vegfa	vascular endothelial growth factor A	7.47
LOC105242423	uncharacterized LOC105242423	7.44
Gm22707	predicted gene, 22707 [Source:MGI Symbol;Acc:MGI:5452484]	7.27
Fam96a	family with sequence similarity 96, member A	7.24
Hap1	huntingtin-associated protein 1	7.19
Gmfb	glia maturation factor, beta	7.17
Zfp566	zinc finger protein 566	7.04
Gnal	guanine nucleotide binding protein, alpha stimulating, olfactory type	6.88
Ccdc62	coiled-coil domain containing 62	6.85

Gm10291	predicted pseudogene 10291 [Source:MGI Symbol;Acc:MGI:3641638]	6.84
Drd2	dopamine receptor D2	6.83
Rasl10b	RAS-like, family 10, member B	6.77
Fgf1	fibroblast growth factor 1	6.71
Ppp1r14a	protein phosphatase 1, regulatory (inhibitor) subunit 14A	6.71
Gm24241	predicted gene, 24241 [Source:MGI Symbol;Acc:MGI:5454018]	6.65
Eif4ebp3; Ankhd1	eukaryotic translation initiation factor 4E binding protein 3; ankyrin repeat and KH domain containing 1	6.47
Prkci	protein kinase C, iota	6.43
Nicn1	nicolin 1	6.3
Sirt2	sirtuin 2	6.25
DQ267101	snoRNA DQ267101	6.07
Gm25537	predicted gene, 25537 [Source:MGI Symbol;Acc:MGI:5455314]	6.03
Mbnl1	muscleblind-like 1 (Drosophila)	5.98
Gm24704	predicted gene, 24704 [Source:MGI Symbol;Acc:MGI:5454481]	5.93
Chchd3; Gm40360	coiled-coil-helix-coiled-coil-helix domain containing 3; predicted gene, 40360	5.83
Gjc3	gap junction protein, gamma 3	5.82
Ssr2	signal sequence receptor, beta	5.61
		5.56
Gm15802	predicted gene 15802	5.49
Crip1	cysteine-rich protein 1 (intestinal)	5.45
Snord17	small nucleolar RNA, C/D box 17	5.45
Olfr698	olfactory receptor 698	5.4
Olfr1269	olfactory receptor 1269	5.38
Stxbp3-ps	syntaxin-binding protein 3, pseudogene	5.37
Mrps11	mitochondrial ribosomal protein S11	5.33
Olfr120	olfactory receptor 120	5.31
Diaph2	diaphanous related formin 2	5.26
Rab9	RAB9, member RAS oncogene family	5.25
Efcab1	EF hand calcium binding domain 1	5.21
H2-Ab1	histocompatibility 2, class II antigen A, beta 1	5.13
Pglyrp1	peptidoglycan recognition protein 1	5.12
Gm10754	predicted gene 10754	5.12
Abhd5	abhydrolase domain containing 5	5.06
		4.96
Rab21	RAB21, member RAS oncogene family	4.93
		4.92

Immp2l	IMP2 inner mitochondrial membrane peptidase-like (S. cerevisiae)	4.9
Car7	carbonic anhydrase 7	4.85
Gm24706	predicted gene, 24706 [Source:MGI Symbol;Acc:MGI:5454483]	4.72
Smpd3	sphingomyelin phosphodiesterase 3, neutral	4.64
Dgat1	diacylglycerol O-acyltransferase 1	4.48
Cct4	chaperonin containing Tcp1, subunit 4 (delta)	4.47
Rhod	ras homolog gene family, member D	4.46
Cisd1	CDGSH iron sulfur domain 1	4.45
Gm25053	predicted gene, 25053 [Source:MGI Symbol;Acc:MGI:5454830]	4.44
Gm25696	predicted gene, 25696 [Source:MGI Symbol;Acc:MGI:5455473]	4.42
Phf5a	PHD finger protein 5A	4.41
Trdv2-1	T cell receptor delta variable 2-1	4.4
Gm23975	predicted gene, 23975 [Source:MGI Symbol;Acc:MGI:5453752]	4.3
Tmem258	transmembrane protein 258	4.29
Ces1b	carboxylesterase 1B	4.28
Ift27	intraflagellar transport 27	4.23
Mir290a	microRNA 290a	4.17
Wfdc18	WAP four-disulfide core domain 18	4.17
Gm7367	1110014K08Rik pseudogene	4.14
Galnt18	UDP-N-acetyl-alpha-D-galactosamine:polypeptide N-acetylgalactosaminyltransferase 18	4.1
Efnb3	ephrin B3	4.09
Mir3061; Ppp2ca	microRNA 3061; protein phosphatase 2 (formerly 2A), catalytic subunit, alpha isoform	4.05
Rnf13	ring finger protein 13	3.93
Asun	asunder, spermatogenesis regulator	3.93
Lsm4	LSM4 homolog, U6 small nuclear RNA associated (S. cerevisiae)	3.9
M6pr	mannose-6-phosphate receptor, cation dependent	3.89
Gm24620	predicted gene, 24620 [Source:MGI Symbol;Acc:MGI:5454397]	3.88
Igkv4-74	immunoglobulin kappa variable 4-74	3.88
Olfr761	olfactory receptor 761	3.87
Ighv2-4	immunoglobulin heavy variable V2-4	3.86
Lxn	latexin	3.86
Syne1	spectrin repeat containing, nuclear envelope 1	3.84
		3.84

Snord42b	small nucleolar RNA, C/D box 42B	3.82
		3.82
Zfp781	zinc finger protein 781	3.8
Timm8a1	translocase of inner mitochondrial membrane 8A1	3.8
Ift57	intraflagellar transport 57	3.78
Rpl36a	ribosomal protein L36A	3.78
		3.77
Gm24727	predicted gene, 24727 [Source:MGI Symbol;Acc:MGI:5454504]	3.77
Klc4	kinesin light chain 4	3.74
1700024B05Rik	RIKEN cDNA 1700024B05 gene	3.72
2810403A07Rik	RIKEN cDNA 2810403A07 gene	3.72
Gins4	GINS complex subunit 4 (Sld5 homolog)	3.69
2810410L24Rik	RIKEN cDNA 2810410L24 gene	3.64
Tm6sf1	transmembrane 6 superfamily member 1	3.64
Znhit1	zinc finger, HIT domain containing 1	3.59
H2-Eb1	histocompatibility 2, class II antigen E beta	3.57
Gm24068	predicted gene, 24068 [Source:MGI Symbol;Acc:MGI:5453845]	3.56
Mrps10	mitochondrial ribosomal protein S10	3.54
Mir181a-2	microRNA 181a-2	3.53
		3.51
Syne1	spectrin repeat containing, nuclear envelope 1	3.48
Rnf149	ring finger protein 149	3.45
Gm3286	predicted gene 3286	3.4
Gm25224	predicted gene, 25224 [Source:MGI Symbol;Acc:MGI:5455001]	3.39
Efcab2	EF-hand calcium binding domain 2	3.37
Trav16n	T cell receptor alpha variable 16n	3.37
Gm25617	predicted gene, 25617 [Source:MGI Symbol;Acc:MGI:5455394]	3.34
Syndig1l	synapse differentiation inducing 1 like	3.34
Gm38438	predicted gene, 38438	3.33
Saa2	serum amyloid A 2	3.29
Fam168a	family with sequence similarity 168, member A	3.28
Gm24696	predicted gene, 24696 [Source:MGI Symbol;Acc:MGI:5454473]	3.23
Scarna2	small Cajal body-specific RNA 2	3.22
Rlf	rearranged L-myc fusion sequence	3.22
		3.21
Gm15455	predicted gene 15455	3.2
Gm23932	predicted gene, 23932 [Source:MGI Symbol;Acc:MGI:5453709]	3.18

Klf1	Kruppel-like factor 1 (erythroid)	3.18
Ecsit	ECSIT homolog (Drosophila)	3.18
Scfd1	Sec1 family domain containing 1	3.16
Gm26376	predicted gene, 26376 [Source:MGI Symbol;Acc:MGI:5456153]	3.15
4933406D12Rik	RIKEN cDNA 4933406D12 gene	3.13
Gm14085	predicted gene 14085	3.1
Gm22039	predicted gene, 22039 [Source:MGI Symbol;Acc:MGI:5451816]	3.06
		3.04
Olfr513	olfactory receptor 513	3.04
Slc35d3	solute carrier family 35, member D3	3.03
		3.02
		3.01
Mir1905	microRNA 1905	3
Trav6-1	T cell receptor alpha variable 6-1	2.99
Gm25342	predicted gene, 25342 [Source:MGI Symbol;Acc:MGI:5455119]	2.98
Gm6139	predicted gene 6139 [Source:MGI Symbol;Acc:MGI:3643279]	2.98
Platr25	pluripotency associated transcript 25	2.97
Prpf40b	PRP40 pre-mRNA processing factor 40 homolog B (yeast)	2.97
Gm23746	predicted gene, 23746 [Source:MGI Symbol;Acc:MGI:5453523]	2.95
Luzp4	leucine zipper protein 4	2.95
Maneal	mannosidase, endo-alpha-like	2.94
		2.9
Sct	secretin	2.89
Nkain1	Na+/K+ transporting ATPase interacting 1	2.88
Adap1	ArfGAP with dual PH domains 1	2.88
Gm5424; Ass1	argininosuccinate synthase pseudogene; argininosuccinate synthetase 1	2.85
Gm3543	predicted gene 3543 [Source:MGI Symbol;Acc:MGI:3781720]	2.85
Olfr855	olfactory receptor 855	2.84
		2.84
Fbln7	fibulin 7	2.81
Kcnn3	potassium intermediate/small conductance calcium-activated channel, subfamily N, member 3	2.81
		2.8
Gm22763	predicted gene, 22763 [Source:MGI Symbol;Acc:MGI:5452540]	2.79

Mir199a-1	microRNA 199a-1	2.79
Taf12	TAF12 RNA polymerase II, TATA box binding protein (TBP)-associated factor	2.78
Gm25978	predicted gene, 25978 [Source:MGI Symbol;Acc:MGI:5455755]	2.77
Rps6	ribosomal protein S6	2.76
Olfr293	olfactory receptor 293	2.76
Gm11592	predicted gene 11592	2.75
Pramel3	preferentially expressed antigen in melanoma-like 3	2.74
AA692955	expressed sequence AA692955	2.73
Tspan2	tetraspanin 2	2.72
Gm14420	predicted gene 14420	2.72
Mup20; Mup-ps19	major urinary protein 20; major urinary protein, pseudogene 19	2.71
Bhlha9	basic helix-loop-helix family, member a9	2.7
C230029F24Rik	RIKEN cDNA C230029F24 gene	2.69
Gm22095	predicted gene, 22095 [Source:MGI Symbol;Acc:MGI:5451872]	2.68
Mettl7a1	methyltransferase like 7A1	2.67
Atg4c	autophagy related 4C, cysteine peptidase	2.67
4930431F12Rik	RIKEN cDNA 4930431F12 gene	2.67
B4galnt4	beta-1,4-N-acetyl-galactosaminyl transferase 4	2.67
Stk-ps2	serine/threonine kinase 2	2.66
Pard6a	par-6 family cell polarity regulator alpha	2.66
Gm24670	predicted gene, 24670 [Source:MGI Symbol;Acc:MGI:5454447]	2.64
Gm8692	predicted gene 8692	2.62
D630014O11Rik	RIKEN cDNA D630014O11 gene [Source:MGI Symbol;Acc:MGI:2442874]	2.6
Timm17b	translocase of inner mitochondrial membrane 17b	2.6
Gm25634	predicted gene, 25634 [Source:MGI Symbol;Acc:MGI:5455411]	2.59
Rab4a	RAB4A, member RAS oncogene family	2.59
Speer4a	spermatogenesis associated glutamate (E)-rich protein 4A	2.57
Zfp808	zinc finger protein 80	2.57
Tmem19	transmembrane protein 19	2.56
Gm11011	predicted gene 11011 [Source:MGI Symbol;Acc:MGI:3779229]	2.56
Gm26358	predicted gene, 26358 [Source:MGI Symbol;Acc:MGI:5456135]	2.54
		2.53

Ighv11-1	immunoglobulin heavy variable 11-1	2.51
Gm10735	predicted gene 10735 [Source:MGI Symbol;Acc:MGI:3642476]	2.51
H2-T10; H2-T22	histocompatibility 2, T region locus 10; histocompatibility 2, T region locus 22	2.5
Cdk5rap1	CDK5 regulatory subunit associated protein 1	2.5
Gm26423	predicted gene, 26423 [Source:MGI Symbol;Acc:MGI:5456200]	2.49
Rdh9	retinol dehydrogenase 9	2.48
Gm15940	predicted gene 15940 [Source:MGI Symbol;Acc:MGI:3802082]	2.48
9430024E24Rik	RIKEN cDNA 9430024E24 gene	2.46
Lat	linker for activation of T cells	2.46
Gm21889; Gm21920	predicted gene, 21889 [Source:MGI Symbol;Acc:MGI:5434053]; predicted gene, 21920 [Source:MGI Symbol;Acc:MGI:5434084]	2.46
2-Sep	septin 2	2.44
Tcf20	transcription factor 20	2.44
Olfr618	olfactory receptor 618	2.44
		2.44
Ass1	argininosuccinate synthetase 1	2.42
Gm23196	predicted gene, 23196 [Source:MGI Symbol;Acc:MGI:5452973]	2.41
Ppy	pancreatic polypeptide	2.4
Gm24987	predicted gene, 24987 [Source:MGI Symbol;Acc:MGI:5454764]	2.4
Gm25056	predicted gene, 25056 [Source:MGI Symbol;Acc:MGI:5454833]	2.39
Igkv4-58	immunoglobulin kappa variable 4-58	2.38
Wwp2	WW domain containing E3 ubiquitin protein ligase 2	2.38
Cpsf4l	cleavage and polyadenylation specific factor 4-like	2.37
		2.37
AV051173	expressed sequence AV051173	2.35
Magea3	melanoma antigen, family A, 3	2.34
Gm24665	predicted gene, 24665 [Source:MGI Symbol;Acc:MGI:5454442]	2.33
A630075F10Rik	RIKEN cDNA A630075F10 gene	2.33
		2.32
		2.31
Gm3428	predicted gene 3428	2.31

Chchd2	coiled-coil-helix-coiled-coil-helix domain containing 2	2.3
Ifnl2	interferon lambda 2	2.3
Fbxw25	F-box and WD-40 domain protein 25	2.3
9930111J21Rik2	RIKEN cDNA 9930111J21 gene 2	2.28
Olfr1265	olfactory receptor 1265	2.26
		2.24
Acad12	acyl-Coenzyme A dehydrogenase family, member 12	2.23
Gm26173	predicted gene, 26173 [Source:MGI Symbol;Acc:MGI:5455950]	2.22
Gm23054	predicted gene, 23054 [Source:MGI Symbol;Acc:MGI:5452831]	2.22
Mir30e	microRNA 30e	2.21
Ric8b	resistance to inhibitors of cholinesterase 8 homolog B (C. elegans)	2.2
Prss28	protease, serine 28	2.2
Zfp114	zinc finger protein 114	2.2
Mfsd11	major facilitator superfamily domain containing 11	2.19
Gm12298	predicted gene 12298	2.18
Mir346	microRNA 346	2.16
Gm23585	predicted gene, 23585 [Source:MGI Symbol;Acc:MGI:5453362]	2.14
Stk10	serine/threonine kinase 10	2.14
Olfr1143	olfactory receptor 1143	2.13
Gm22271	predicted gene, 22271 [Source:MGI Symbol;Acc:MGI:5452048]	2.11
Gm17361	predicted gene, 17361	2.11
Zfp273	zinc finger protein 273	2.1
Gm24840	predicted gene, 24840 [Source:MGI Symbol;Acc:MGI:5454617]	2.1
Fthl17b	ferritin, heavy polypeptide-like 17, member B	2.1
		2.1
Olfr239	olfactory receptor 239	2.09
1700012B09Rik	RIKEN cDNA 1700012B09 gene	2.09
Gm8246	predicted gene 8246	2.08
n-R5s197	nuclear encoded rRNA 5S 197 [Source:MGI Symbol;Acc:MGI:4422062]	2.06
Gm22301	predicted gene, 22301 [Source:MGI Symbol;Acc:MGI:5452078]	2.06
Traj6	T cell receptor alpha joining 6	2.05
Ctcfl	CCCTC-binding factor (zinc finger protein)-like	2.05

Gm1965	predicted gene 1965	2.05
Mir1941	microRNA 1941	2.04
Olfr1290	olfactory receptor 1290	2.04
Gm7237	predicted gene 7237 [Source:MGI Symbol;Acc:MGI:3779701]	2.03
		2.03
Gm12531	predicted gene 12531 [Source:MGI Symbol;Acc:MGI:3650531]	2.03
Gm7935	predicted pseudogene 7935 [Source:MGI Symbol;Acc:MGI:3646370]	2.02
Gm23287	predicted gene, 23287 [Source:MGI Symbol;Acc:MGI:5453064]	2.01
		-2.04
Insig1	insulin induced gene 1	-2.06
AI593442	expressed sequence AI593442	-2.17
Pcsk2	proprotein convertase subtilisin/kexin type 2	-2.18
B2m	beta-2 microglobulin	-2.37
Idh3g	isocitrate dehydrogenase 3 (NAD+), gamma	-2.4
Yipf5	Yip1 domain family, member 5	-2.45
Camk2n1	calcium/calmodulin-dependent protein kinase II inhibitor 1	-2.49
Gm25402	predicted gene, 25402 [Source:MGI Symbol;Acc:MGI:5455179]	-2.49
Ankrd12	ankyrin repeat domain 12	-2.5
Rab1a	RAB1A, member RAS oncogene family	-2.53
Ncbp3	nuclear cap binding subunit 3	-2.57
Adipor2	adiponectin receptor 2	-2.62
Cdc123	cell division cycle 123	-2.64
B3glct	beta-3-glucosyltransferase	-2.69
Crmp1	collapsin response mediator protein 1	-2.75
Bap1	Brca1 associated protein 1	-2.77
Lmbrd2	LMBR1 domain containing 2	-2.77
Gm5454	predicted gene 5454	-2.79
Gsk3b	glycogen synthase kinase 3 beta	-2.8
Mbd2	methyl-CpG binding domain protein 2	-2.8
Rbfox1	RNA binding protein, fox-1 homolog (C. elegans) 1	-2.82
Clk4	CDC like kinase 4	-2.86
Zfp799	zinc finger protein 799	-2.86
		-2.86
Dazap2	DAZ associated protein 2	-2.88
		-2.89
Hist1h2an	histone cluster 1, H2an	-2.94
Zfp131	zinc finger protein 131	-2.95

Psma1	proteasome (prosome, macropain) subunit, alpha type 1	-3
		-3.03
LOC100041599	protein FAM205A-like	-3.14
Gm25432	predicted gene, 25432	-3.16
Rnf4	ring finger protein 4	-3.17
Hnrnpa3	heterogeneous nuclear ribonucleoprotein A3	-3.19
LOC105243690; Gm14681	nucleophosmin-like; predicted gene 14681 [Source:MGI Symbol;Acc:MGI:3705734]	-3.22
Add1	adducin 1 (alpha)	-3.24
Aldoc	aldolase C, fructose-bisphosphate	-3.26
Sub1	SUB1 homolog (S. cerevisiae)	-3.28
Ddx6	DEAD (Asp-Glu-Ala-Asp) box polypeptide 6	-3.29
Sort1	sortilin 1	-3.3
Scn1b	sodium channel, voltage-gated, type I, beta	-3.3
Gm21887	predicted gene, 21887 [Source:MGI Symbol;Acc:MGI:5434051]	-3.3
Serbp1	serpine1 mRNA binding protein 1	-3.33
Ubap1	ubiquitin-associated protein 1	-3.38
Lamp1	lysosomal-associated membrane protein 1	-3.4
Uqcrc1	ubiquinol-cytochrome c reductase core protein 1	-3.41
Arpc2	actin related protein 2/3 complex, subunit 2	-3.42
Exoc3	exocyst complex component 3	-3.42
Nop10	NOP10 ribonucleoprotein	-3.54
Ate1	arginyltransferase 1	-3.55
Slc17a5	solute carrier family 17 (anion/sugar transporter), member 5	-3.56
Gm12238	predicted gene 12238	-3.59
Ntrk2	neurotrophic tyrosine kinase, receptor, type 2	-3.62
Gyg	glycogenin	-3.63
Usp9x	ubiquitin specific peptidase 9, X chromosome	-3.65
Cox7a2l	cytochrome c oxidase subunit VIIa polypeptide 2-like	-3.66
Med13l	mediator complex subunit 13-like	-3.67
Rhoa	ras homolog gene family, member A	-3.69
4833439L19Rik	RIKEN cDNA 4833439L19 gene	-3.7
Pfn1	profilin 1	-3.71
Btf3l4	basic transcription factor 3-like 4	-3.77
4933431E20Rik	RIKEN cDNA 4933431E20 gene	-3.79
Atp6ap2	ATPase, H+ transporting, lysosomal accessory protein 2	-3.79
Dpf2	D4, zinc and double PHD fingers family 2	-3.8

Ddx26b	DEAD/H (Asp-Glu-Ala-Asp/His) box polypeptide 26B	-3.81
Ldb2	LIM domain binding 2	-3.91
Sft2d1	SFT2 domain containing 1	-3.94
n-R5s8	nuclear encoded rRNA 5S 8 [Source:MGI Symbol;Acc:MGI:4421742]	-3.94
Gm24671	predicted gene, 24671 [Source:MGI Symbol;Acc:MGI:5454448]	-3.99
Bsg	basigin	-4
Gm14296; 2210418O10Rik	predicted gene 14296; predicted gene 2210418O10Rik	-4
2900055J20Rik	RIKEN cDNA 2900055J20 gene	-4.06
Katna1	katanin p60 (ATPase-containing) subunit A1	-4.07
Smg7	Smg-7 homolog, nonsense mediated mRNA decay factor (C. elegans)	-4.08
2310057M21Rik	RIKEN cDNA 2310057M21 gene	-4.08
Mllt11	myeloid/lymphoid or mixed-lineage leukemia (trithorax homolog, Drosophila); translocated to, 11	-4.14
Pdhb	pyruvate dehydrogenase (lipoamide) beta	-4.15
Slc24a2	solute carrier family 24 (sodium/potassium/calcium exchanger), member 2	-4.15
Slc25a44	solute carrier family 25, member 44	-4.18
Rpl4	ribosomal protein L4	-4.18
Polr1c	polymerase (RNA) I polypeptide C	-4.21
LOC102637606	protein FAM205A-like	-4.23
Skp1a	S-phase kinase-associated protein 1A	-4.26
Slc37a3	solute carrier family 37 (glycerol-3-phosphate transporter), member 3	-4.26
Ranbp9	RAN binding protein 9	-4.27
		-4.28
Rpl7a; Rpl7a-ps11	ribosomal protein L7A; ribosomal protein L7A, pseudogene 11	-4.3
Anks1b	ankyrin repeat and sterile alpha motif domain containing 1B	-4.36
Taf1b	TATA box binding protein (Tbp)-associated factor, RNA polymerase I, B	-4.36
Slc25a28	solute carrier family 25, member 28	-4.37
Snapc3	small nuclear RNA activating complex, polypeptide 3	-4.38
Rit2	Ras-like without CAAX 2	-4.41
Lin7b	lin-7 homolog B (C. elegans)	-4.46
Ccdc159	coiled-coil domain containing 159	-4.5

Cpeb2	cytoplasmic polyadenylation element binding protein 2	-4.53
Arl8b	ADP-ribosylation factor-like 8B	-4.54
Edem2	ER degradation enhancer, mannosidase alpha-like 2	-4.55
Clta	clathrin, light polypeptide (Lca)	-4.56
Zc4h2	zinc finger, C4H2 domain containing	-4.56
Sobp	sine oculis-binding protein homolog (Drosophila)	-4.6
S100a1	S100 calcium binding protein A1	-4.62
Slc25a5	solute carrier family 25 (mitochondrial carrier, adenine nucleotide translocator), member 5	-4.69
Adarb1	adenosine deaminase, RNA-specific, B1	-4.71
Gm11353	predicted gene 11353 [Source:MGI Symbol;Acc:MGI:3649794]	-4.74
Dph6	diphthamine biosynthesis 6	-4.81
Chst1	carbohydrate (keratan sulfate Gal-6) sulfotransferase 1	-4.82
Smn1	survival motor neuron 1	-4.84
Peak1	pseudopodium-enriched atypical kinase 1	-4.84
Churc1; Fntb	churchill domain containing 1; farnesyltransferase, CAAX box, beta	-4.94
		-4.96
Mdh2	malate dehydrogenase 2, NAD (mitochondrial)	-5.02
Gm16357	predicted gene 16357	-5.03
Shoc2	soc-2 (suppressor of clear) homolog (C. elegans)	-5.06
Gm14325; Gm14326	predicted gene 14325; predicted gene 14326	-5.08
Prpf18	PRP18 pre-mRNA processing factor 18 homolog (yeast)	-5.15
Lpcat4	lysophosphatidylcholine acyltransferase 4	-5.16
Slc35g2	solute carrier family 35, member G2	-5.16
Gm6710; Gm14391	predicted gene 6710; predicted gene 14391	-5.22
Pcdh7	protocadherin 7	-5.25
Dgka	diacylglycerol kinase, alpha	-5.38
Smim11	small integral membrane protein 11	-5.42
		-5.44
Lrfn5	leucine rich repeat and fibronectin type III domain containing 5	-5.45
Cops6	COP9 (constitutive photomorphogenic) homolog, subunit 6 (Arabidopsis thaliana)	-5.46
Gm23546	predicted gene, 23546 [Source:MGI Symbol;Acc:MGI:5453323]	-5.49
Ttyh1	tweety homolog 1 (Drosophila)	-5.53

Eif3j2; Eif3j1	eukaryotic translation initiation factor 3, subunit J2; eukaryotic translation initiation factor 3, subunit J1	-5.58
Ddx5	DEAD (Asp-Glu-Ala-Asp) box polypeptide 5	-5.6
Jkamp	JNK1/MAPK8-associated membrane protein	-5.67
Gars	glycyl-tRNA synthetase	-5.76
Selt	selenoprotein T	-5.77
Hist2h2aa1; Hist2h2aa2	histone cluster 2, H2aa1; histone cluster 2, H2aa2	-5.78
Hist2h2aa2; Hist2h2aa1	histone cluster 2, H2aa2; histone cluster 2, H2aa1	-5.78
Shank1	SH3/ankyrin domain gene 1	-5.8
Ccdc124	coiled-coil domain containing 124	-5.8
Syne1	spectrin repeat containing, nuclear envelope 1	-5.81
Gm22953	predicted gene, 22953 [Source:MGI Symbol;Acc:MGI:5452730]	-5.81
Ggnbp2	gametogenetin binding protein 2	-5.85
Gm1673	predicted gene 1673	-5.88
		-5.9
Ift22	intraflagellar transport 22	-5.91
Tmem59l	transmembrane protein 59-like	-5.92
		-5.92
Arl15	ADP-ribosylation factor-like 15	-6.21
Gm17167; Gm29964; Gm21075; Gm13301	predicted gene 17167; predicted gene, 29964; predicted gene, 21075; predicted gene 13301	-6.39
Gm15590; Gm22774	predicted gene 15590 [Source:MGI Symbol;Acc:MGI:3831433]; predicted gene, 22774 [Source:MGI Symbol;Acc:MGI:5452551]	-6.4
Mir692-3; Mir692-2; Ftl1; Ftl2-ps	microRNA 692-3; microRNA 692-2; ferritin light polypeptide 1; ferritin light polypeptide 2, pseudogene	-6.4
Mvb12b	multivesicular body subunit 12B	-6.43
Zmym2	zinc finger, MYM-type 2	-6.44
Sdf2	stromal cell derived factor 2	-6.51
Cdc26	cell division cycle 26	-6.53
Gm14308; Gm14430; Gm14434; Gm4724; Gm11007	predicted gene 14308; predicted gene 14430; predicted gene 14434; predicted gene 4724; predicted gene 11007	-6.55
Gm14308; Gm14430; Gm14434; Gm4724; Gm11007	predicted gene 14308; predicted gene 14430; predicted gene 14434; predicted gene 4724; predicted gene 11007	-6.55
Stx6	syntaxin 6	-6.56
		-6.57

LOC102640399; Gm22131	uncharacterized LOC102640399; predicted gene, 22131 [Source:MGI Symbol;Acc:MGI:5451908]	-6.57
Tctex1d2	Tctex1 domain containing 2	-6.6
Dnajc21	DnaJ (Hsp40) homolog, subfamily C, member 21	-6.62
Scrg1	scrapie responsive gene 1	-6.65
Tceb2	transcription elongation factor B (SIII), polypeptide 2	-6.68
Gria2	glutamate receptor, ionotropic, AMPA2 (alpha 2)	-6.75
Muc3a	mucin 3A, cell surface associated	-6.81
Hnrnph3	heterogeneous nuclear ribonucleoprotein H3	-6.83
Actr2	ARP2 actin-related protein 2	-6.88
Ccng1	cyclin G1	-6.93
Ccdc25	coiled-coil domain containing 25	-6.96
Atp6v1b2	ATPase, H+ transporting, lysosomal V1 subunit B2	-6.99
Rbm7	RNA binding motif protein 7	-7
Mir3075	microRNA 3075	-7.02
Siah1a	seven in absentia 1A	-7.02
Usp33	ubiquitin specific peptidase 33	-7.08
Otud6b	OTU domain containing 6B	-7.13
Cox8a	cytochrome c oxidase subunit VIIIa	-7.14
Gm6710; Gm14391	predicted gene 6710; predicted gene 14391	-7.2
Ttc9b	tetratricopeptide repeat domain 9B	-7.24
Itm2b	integral membrane protein 2B	-7.25
Gm26236	predicted gene, 26236 [Source:MGI Symbol;Acc:MGI:5456013]	-7.3
Ccnt2	cyclin T2	-7.34
Ube2d3	ubiquitin-conjugating enzyme E2D 3	-7.36
Gm23301	predicted gene, 23301 [Source:MGI Symbol;Acc:MGI:5453078]	-7.44
Gm14308; Gm14430; Gm14434; Gm4724; Gm11007	predicted gene 14308; predicted gene 14430; predicted gene 14434; predicted gene 4724; predicted gene 11007	-7.51
S100a6	S100 calcium binding protein A6 (calcyclin)	-7.66
Pdcd4	programmed cell death 4	-7.67
Mir668	microRNA 668	-7.85
Nmt1	N-myristoyltransferase 1	-7.86
Gm22188	predicted gene, 22188 [Source:MGI Symbol;Acc:MGI:5451965]	-7.89
Grpel1	GrpE-like 1, mitochondrial	-8.11
15-Sep	selenoprotein	-8.15

Gm22858	predicted gene, 22858 [Source:MGI Symbol;Acc:MGI:5452635]	-8.25
Syne1	spectrin repeat containing, nuclear envelope 1	-8.53
Ndufa4	NADH dehydrogenase (ubiquinone) 1 alpha subcomplex, 4	-8.83
Wdr82	WD repeat domain containing 82	-8.87
		-8.95
Pomp	proteasome maturation protein	-8.98
Nop56	NOP56 ribonucleoprotein	-9.15
Max	Max protein	-9.4
Slc7a5	solute carrier family 7 (cationic amino acid transporter, y+ system), member 5	-9.41
Fam160a1; Arfip1	family with sequence similarity 160, member A1; ADP-ribosylation factor interacting protein 1	-9.43
Psmb4	proteasome (prosome, macropain) subunit, beta type 4	-9.76
Gm6415	predicted pseudogene 6415 [Source:MGI Symbol;Acc:MGI:3648526]	-9.89
		-10.07
Lrrtm3	leucine rich repeat transmembrane neuronal 3	-10.15
Gm2506	predicted gene 2506	-10.15
Gm2506	predicted gene 2506	-10.15
Gm2506	predicted gene 2506	-10.15
Atxn10	ataxin 10	-10.18
Snhg14	small nucleolar RNA host gene 14	-10.47
Psma2	proteasome (prosome, macropain) subunit, alpha type 2	-10.57
Gm16089	predicted gene 16089 [Source:MGI Symbol;Acc:MGI:3801884]	-10.59
R3hdm1	R3H domain containing 1	-10.69
Syne1	spectrin repeat containing, nuclear envelope 1	-10.73
		-10.98
Gm21399	predicted gene, 21399 [Source:MGI Symbol;Acc:MGI:5434754]	-11.01
Mpc1; Mpc1-ps	mitochondrial pyruvate carrier 1; mitochondrial pyruvate carrier 1, pseudogene	-11.08
Cck	cholecystokinin	-11.22
Gm3579	predicted gene 3579	-11.25
Gm42035; Gm3579	predicted gene, 42035; predicted gene 3579	-11.25
Gm3579	predicted gene 3579	-11.25
Gm42035; Gm3579	predicted gene, 42035; predicted gene 3579	-11.25
Tmem196	transmembrane protein 196	-11.4
1700025G04Rik	RIKEN cDNA 1700025G04 gene	-11.62

Rnf7	ring finger protein 7	-11.88
Gm10053	predicted gene 10053 [Source:MGI Symbol;Acc:MGI:3704493]	-11.9
Cycs	cytochrome c, somatic	-11.9
Cdr1	cerebellar degeneration related antigen 1	-11.99
Gm26202	predicted gene, 26202 [Source:MGI Symbol;Acc:MGI:5455979]	-12.12
Emc7	ER membrane protein complex subunit 7	-12.39
Nell2; Gm30810	NEL-like 2; predicted gene, 30810	-12.8
Nutf2-ps1; Nutf2-ps2	nuclear transport factor 2, pseudogene 1; nuclear transport factor 2, pseudogene 2	-12.8
Sv2b	synaptic vesicle glycoprotein 2 b	-13.01
Mir690	microRNA 690	-13.56
		-13.88
Snord65	small nucleolar RNA, C/D box 65	-14.13
Ube2d2a	ubiquitin-conjugating enzyme E2D 2A	-15.02
Gm2260; Gm2274	predicted gene 2260; predicted gene 2274	-15.1
Gm2260; Gm2274	predicted gene 2260; predicted gene 2274	-15.1
		-15.72
		-16.2
Gm26265	predicted gene, 26265 [Source:MGI Symbol;Acc:MGI:5456042]	-16.39
		-16.88
DQ267100; Rian	snoRNA DQ267100; RNA imprinted and accumulated in nucleus	-17.77
Taf1d	TATA box binding protein (Tbp)-associated factor, RNA polymerase I, D	-17.99
Gm26347	predicted gene, 26347 [Source:MGI Symbol;Acc:MGI:5456124]	-18.68
Mir329	microRNA 329	-18.77
Gm25147	predicted gene, 25147 [Source:MGI Symbol;Acc:MGI:5454924]	-18.86
Tmem167b	transmembrane protein 167B	-19.21
Prrt2; Pagr1a	proline-rich transmembrane protein 2; PAXIP1 associated glutamate rich protein 1A	-19.34
Slc17a7	solute carrier family 17 (sodium-dependent inorganic phosphate cotransporter), member 7	-21.75
Vsnl1	visinin-like 1	-22.37
Mir485; Mirg	microRNA 485; miRNA containing gene	-26.3
Purb	purine rich element binding protein B	-28.05
Fabp5	fatty acid binding protein 5, epidermal	-28.82
Fabp5	fatty acid binding protein 5, epidermal	-29.84
Rabac1	Rab acceptor 1 (prenylated)	-29.98
Anapc13	anaphase promoting complex subunit 13	-31.67

Snord61	small nucleolar RNA, C/D box 61	-34.09
Mir376b	microRNA 376b	-35.97
Atxn7l1	ataxin 7-like 1	-54.63
Bmpr2	bone morphogenetic protein receptor, type II (serine/threonine kinase)	-148.74

Table S6 Transcription factors affected by Western diet and LDL-R -/- in hippocampal microvascular endothelium.

WT WD vs WT CD	
Name	***p-Value***
CREB1	4.940E-187
c-Myc	1.890E-119
ESR1 (nuclear)	3.700E-89
YY1	4.100E-74
RelA (p65 NF-kB subunit)	4.100E-74
Androgen receptor	4.080E-68
SP1	4.030E-65
STAT3	3.850E-59
p53	3.850E-59
HNF4-alpha	3.400E-50
c-Jun	3.400E-50
NANOG	2.800E-41
ZNF143	2.800E-41
Oct-3/4	2.580E-38
C/EBPbeta	2.580E-38
ETS1	2.580E-38
RUNX2	2.350E-35
SOX2	2.350E-35
TAL1	2.350E-35
HIF1A	2.350E-35
MYOD	2.350E-35
GATA-3	2.350E-35
SMAD3	2.120E-32
GATA-1	2.120E-32
MYOG	2.120E-32
HSF1	2.120E-32

LDL-R -/- CD vs WT CD	
Name	***p-Value***
CREB1	0.000E+00
c-Myc	0.000E+00
YY1	0.000E+00
SP1	4.050E-302
ESR1 (nuclear)	2.090E-272
Oct-3/4	1.940E-256
p53	2.530E-247
RelA (p65 NF-kB subunit)	9.070E-243
c-Jun	6.200E-211
NANOG	1.160E-208
Androgen receptor	2.570E-197
SOX2	4.760E-195
HIF1A	8.810E-193
E2F1	3.010E-188
NRSF	1.020E-183
STAT3	8.020E-179
GCR	3.930E-170
GATA-3	1.320E-165
SRF	1.320E-165
c-Fos	4.380E-161
LHX2	4.380E-161
C/EBPbeta	4.380E-161
SP3	4.380E-161
IRF4	7.990E-159
NF-kB1 (p50)	1.450E-156
HNF4-alpha	1.450E-156

LDL-R -/- WD vs WT WD	
Name	***p-Value***
CREB1	0.000E+00
c-Myc	9.110E-255
ESR1 (nuclear)	2.110E-139
SP1	1.100E-117
Androgen receptor	1.880E-101
p53	2.360E-93
YY1	1.170E-90
RelA (p65 NF-kB subunit)	2.870E-85
ZNF143	1.650E-74
Oct-3/4	8.020E-72
c-Jun	8.960E-64
SOX2	8.960E-64
HIF1A	4.280E-61
NANOG	4.280E-61
HNF4-alpha	2.040E-58
E2F1	4.560E-53
NF-kB1 (p50)	2.140E-50
C/EBPbeta	2.140E-50
PPAR-gamma	1.000E-47
STAT3	4.650E-45
EGR1	4.650E-45
SRF	9.880E-40
KLF4	9.880E-40
SMAD4	4.510E-37
NRF1	4.510E-37
FKHR	4.510E-37
